# Supplementary material for: Randomized single oral dose phase 1 study of safety, tolerability, and pharmacokinetics of Iminosugar UV-4 Hydrochloride (UV-4B) in healthy subjects
Source: PLoS Negl Trop Dis. 2022 Aug 8;16(8):e0010636. doi: 10.1371/journal.pntd.0010636 (PMC9387934; doi:10.1371/journal.pntd.0010636)
Supplement: S1 Table — (DOCX) [file pntd.0010636.s001.docx]

| SI 1:  Summary of Key UV-4 Pharmacokinetic Parameters | | | | | | | | | | | | |
| --- | --- | --- | --- | --- | --- | --- | --- | --- | --- | --- | --- | --- |
| **UV-4B (mg)/ Statistics** | **AUC_(0-inf)_ (ng·h/mL)** | **AUC_(0-8)_ (ng·h/mL)** | **C_max_ (ng/mL)** | **t_max_ (h)[a]** | **t_1/2_ (h)** | **CL/F (L/h)** | **V_z_/F (L)** | **DNAUC_(0-inf)_ (ng·h/mL/mg)** | **DNC_max_ (ng/mL/mg)** | **CL_r_ (L/h)** | **Ae_(0-last)_  (mg)** | **fe_(0-last)_ (%)** |
| **Cohort 1, 3 mg (n=6)** | | | | | | | | | | | | |
| Ar Mean | 95.6 | 56.5 | 22.4 | 0.50 | 10.7 | 32.1 | 491 | 31.9 | 7.45 | 14.5 | 1.19 | 39.8 |
| CV% | 16.3 | 15.4 | 15.9 | ND | 33.2 | 15.9 | 32.3 | 16.3 | 15.9 | 15.0 | 16.8 | 16.7 |
| Minimum | 79.8 | 43.6 | 18.4 | 0.50 | 7.75 | 25.5 | 306 | 26.6 | 6.13 | 12.2 | 0.861 | 28.7 |
| Maximum | 117 | 67.3 | 28.5 | 1.00 | 16.3 | 37.6 | 740 | 39.1 | 9.50 | 17.1 | 1.44 | 48.1 |
| Geo Mean | 94.6 | 55.9 | 22.1 | ND | 10.3 | 31.7 | 470 | 31.5 | 7.38 | 14.3 | ND | ND |
| **Cohort 2, 10 mg (n=6)** | | | | | | | | | | | | |
| Ar Mean | 431 | 271 | 84.6 | 1.00 | 12.0 | 24.3 | 424 | 43.1 | 8.46 | 11.7 | 4.64 | 46.4 |
| CV% | 26.7 | 24.8 | 23.4 | ND | 10.4 | 21.0 | 28.3 | 26.7 | 23.4 | 24.9 | 10.0 | 10.0 |
| Minimum | 339 | 199 | 60.3 | 0.50 | 10.6 | 15.4 | 253 | 33.9 | 6.03 | 7.54 | 3.78 | 37.8 |
| Maximum | 651 | 390 | 107 | 1.50 | 13.7 | 29.5 | 585 | 65.1 | 10.7 | 15.4 | 5.00 | 50.0 |
| Geo Mean | 420 | 265 | 82.6 | ND | 11.9 | 23.8 | 409 | 42.0 | 8.26 | 11.4 | ND | ND |
| **Cohort 3, 30 mg (n=6)** | | | | | | | | | | | | |
| Ar Mean | 1260 | 901 | 293 | 0.50 | 10.0 | 24.0 | 349 | 42.0 | 9.76 | 12.6 | 15.5 | 51.7 |
| CV% | 9.6 | 11.3 | 15.3 | ND | 7.6 | 11.2 | 17.2 | 9.7 | 15.4 | 10.4 | 6.3 | 6.3 |
| Minimum | 1030 | 731 | 205 | 0.50 | 8.75 | 21.7 | 274 | 34.2 | 6.83 | 11.4 | 14.2 | 47.4 |
| Maximum | 1380 | 1040 | 330 | 1.00 | 10.8 | 29.3 | 455 | 46.0 | 11.0 | 14.8 | 16.9 | 56.4 |
| Geo Mean | 1260 | 896 | 289 | ND | 9.98 | 23.9 | 344 | 41.8 | 9.64 | 12.5 | ND | ND |
| **Cohort 4, 90 mg (n=6)** | | | | | | | | | | | | |
| Ar Mean | 3730 | 2800 | 946 | 0.50 | 10.4 | 24.5 | 365 | 41.4 | 10.5 | 13.7 | 49.6 | 55.1 |
| CV% | 13.8 | 8.9 | 34.9 | ND | 27.1 | 12.8 | 24.3 | 13.9 | 35.0 | 13.8 | 7.2 | 7.2 |
| Minimum | 3220 | 2520 | 598 | 0.50 | 8.52 | 19.3 | 294 | 35.7 | 6.64 | 10.9 | 44.9 | 49.9 |
| Maximum | 4650 | 3190 | 1430 | 1.52 | 16.0 | 28.0 | 543 | 51.7 | 15.9 | 16.2 | 54.9 | 61.0 |
| Geo Mean | 3700 | 2790 | 900 | ND | 10.2 | 24.3 | 358 | 41.1 | 10.0 | 13.6 | ND | ND |
| **Cohort 5, 180 mg (n=6)** | | | | | | | | | | | | |
| Ar Mean | 7110 | 5910 | 2150 | 0.75 | 8.71 | 25.4 | 321 | 39.5 | 12.0 | 14.0 | 97.9 | 54.5 |
| CV% | 8.1 | 10.5 | 28.8 | ND | 11.2 | 8.2 | 16.8 | 8.1 | 28.8 | 12.2 | 5.0 | 5.1 |
| Minimum | 6360 | 5210 | 1170 | 0.50 | 7.65 | 22.8 | 252 | 35.4 | 6.50 | 11.9 | 89.1 | 49.5 |
| Maximum | 7880 | 6680 | 2770 | 1.00 | 10.3 | 28.3 | 382 | 43.8 | 15.4 | 16.1 | 102 | 56.6 |
| Geo Mean | 7090 | 5880 | 2060 | ND | 8.66 | 25.4 | 317 | 39.4 | 11.5 | 13.9 | ND | ND |
| **Cohort 6, 360 mg (n=6)** | | | | | | | | | | | | |
| Ar Mean | 13800 | 11900 | 4570 | 0.50 | 8.14 | 26.5 | 309 | 38.4 | 12.7 | 14.1 | 188 | 52.3 |
| CV% | 14.1 | 11.7 | 19.7 | ND | 9.7 | 15.1 | 11.5 | 14.1 | 19.7 | 24.6 | 9.2 | 9.2 |
| Minimum | 11100 | 9960 | 3360 | 0.50 | 7.06 | 22.8 | 251 | 30.7 | 9.33 | 10.9 | 171 | 47.5 |
| Maximum | 15800 | 13700 | 5980 | 1.00 | 9.08 | 32.6 | 356 | 43.8 | 16.6 | 19.7 | 217 | 60.3 |
| Geo Mean | 13700 | 11800 | 4490 | ND | 8.11 | 26.3 | 308 | 38.1 | 12.5 | 13.7 | ND | ND |
| **Cohort 7, 720 mg (n=6)** | | | | | | | | | | | | |
| Ar Mean | 32900 | 29100 | 9930 | 0.75 | 8.19 | 22.1 | 260 | 45.7 | 13.8 | 11.1 | 360 | 49.9 |
| CV% | 10.7 | 12.0 | 20.7 | ND | 6.9 | 11.0 | 10.0 | 10.7 | 20.6 | 18.0 | 9.7 | 9.7 |
| Minimum | 28100 | 25300 | 7980 | 0.50 | 7.66 | 19.5 | 218 | 39.1 | 11.1 | 8.77 | 320 | 44.5 |
| Maximum | 36800 | 33000 | 13200 | 1.00 | 9.23 | 25.6 | 291 | 51.2 | 18.3 | 14.5 | 407 | 56.6 |
| Geo Mean | 32800 | 28900 | 9760 | ND | 8.18 | 22.0 | 259 | 45.5 | 13.6 | 10.9 | ND | ND |
| **Cohort 8, 1000 mg (n=6)** | | | | | | | | | | | | |
| Ar Mean | 43100 | 38700 | 13100 | 1.00 | 8.83 | 23.6 | 299 | 43.1 | 13.1 | 11.9 | 504 | 50.4 |
| CV% | 13.5 | 12.1 | 17.1 | ND | 27.9 | 13.1 | 30.0 | 13.5 | 17.1 | 17.7 | 13.1 | 13.1 |
| Minimum | 35700 | 32700 | 11500 | 0.50 | 6.60 | 19.2 | 227 | 35.7 | 11.5 | 9.78 | 415 | 41.5 |
| Maximum | 52000 | 46500 | 17500 | 1.50 | 13.4 | 28.0 | 469 | 52.0 | 17.5 | 15.0 | 617 | 61.7 |
| Geo Mean | 42800 | 38500 | 13000 | ND | 8.58 | 23.4 | 290 | 42.8 | 13.0 | 11.7 | ND | ND |

Ar: Arithmetic; DN: dose normalized; Geo: geometric; Max: maximum; Min: minimum; ND: not determined.
[a] For t_max_ median and range are presented.

Source: DMID 13-0001 Phase 1 Clinical Study Report, Table 11.2; data summarized from Table 14.2.2, Table 14.2.4, Table 14.2.5
