# Supplementary material for: Randomized single oral dose phase 1 study of safety, tolerability, and pharmacokinetics of Iminosugar UV-4 Hydrochloride (UV-4B) in healthy subjects
Source: PLoS Negl Trop Dis. 2022 Aug 8;16(8):e0010636. doi: 10.1371/journal.pntd.0010636 (PMC9387934; doi:10.1371/journal.pntd.0010636)
Supplement: S2 Table — (DOCX) [file pntd.0010636.s002.docx]

| SI 2:  UV-4 Urinary Recovery | | | | | | | | | | | | | | |
| --- | --- | --- | --- | --- | --- | --- | --- | --- | --- | --- | --- | --- | --- | --- |
| **Recovery** | **By-Interval Amount** | | | | **Cumulative Amount** | | | **By-Interval Percent** | | | | **Cumulative Percent** | | |
| **UV-4B/ Statistics** | **Ae_(0-6)_  (mg)** | **Ae_(6-12)_  (mg)** | **Ae_(12-24)_  (mg)** | **Ae_(24-48)_  (mg)** | **Ae_(0-12)_  (mg)** | **Ae_(0-24)_  (mg)** | **Ae_(0-last)_  (mg)[a]** | **fe_(0-6)_  (%)** | **fe_(6-12)_  (%)** | **fe_(12-24)_  (%)** | **fe_(24-48)_  (%)** | **fe_(0-12)_  (%)** | **fe_(0-24)_  (%)** | **fe_(0-last)_ (%) [a]** |
| **Cohort 1, 3 mg (n=6)** | | | | | | | | | | | | | | |
| Ar Mean | 0.607 | 0.215 | 0.202 | 0.170 | 0.822 | 1.02 | 1.19 | 20.2 | 7.17 | 6.71 | 5.66 | 27.4 | 34.1 | 39.8 |
| CV% | 23.9 | 23.1 | 26.9 | 24.5 | 21.6 | 19.3 | 16.8 | 23.8 | 23.0 | 27.1 | 24.5 | 21.6 | 19.3 | 16.7 |
| Min | 0.434 | 0.141 | 0.127 | 0.109 | 0.575 | 0.712 | 0.861 | 14.5 | 4.71 | 4.22 | 3.64 | 19.2 | 23.7 | 28.7 |
| Max | 0.805 | 0.273 | 0.243 | 0.220 | 1.02 | 1.25 | 1.44 | 26.8 | 9.09 | 8.11 | 7.35 | 34.1 | 41.6 | 48.1 |
| Median | 0.624 | 0.216 | 0.230 | 0.173 | 0.892 | 1.08 | 1.23 | 20.8 | 7.19 | 7.65 | 5.75 | 29.7 | 36.0 | 40.9 |
| **Cohort 2, 10 mg (n=6)** | | | | | | | | | | | | | | |
| Ar Mean | 2.67 | 0.859 | 0.608 | 0.503 | 3.53 | 4.13 | 4.64 | 26.7 | 8.59 | 6.08 | 5.03 | 35.3 | 41.3 | 46.4 |
| CV% | 14.5 | 13.0 | 14.6 | 13.0 | 12.1 | 12.5 | 10.0 | 14.5 | 13.0 | 14.6 | 13.0 | 12.1 | 12.5 | 10.0 |
| Min | 1.99 | 0.705 | 0.440 | 0.411 | 2.75 | 3.19 | 3.78 | 19.9 | 7.05 | 4.40 | 4.11 | 27.5 | 31.9 | 37.8 |
| Max | 3.02 | 1.00 | 0.690 | 0.596 | 3.90 | 4.59 | 5.00 | 30.2 | 10.0 | 6.90 | 5.96 | 39.0 | 45.9 | 50.0 |
| Median | 2.77 | 0.883 | 0.635 | 0.503 | 3.71 | 4.34 | 4.82 | 27.7 | 8.83 | 6.35 | 5.03 | 37.1 | 43.4 | 48.2 |
| **Cohort 3, 30 mg (n=6)** | | | | | | | | | | | | | | |
| Ar Mean | 10.6 | 2.45 | 1.61 | 0.887 | 13.0 | 14.6 | 15.5 | 35.3 | 8.16 | 5.36 | 2.96 | 43.4 | 48.8 | 51.7 |
| CV% | 11.1 | 5.9 | 22.5 | 22.6 | 10.1 | 7.7 | 6.3 | 10.9 | 5.9 | 22.6 | 22.5 | 9.9 | 7.6 | 6.3 |
| Min | 9.44 | 2.29 | 1.14 | 0.566 | 11.7 | 13.2 | 14.2 | 31.5 | 7.62 | 3.80 | 1.89 | 39.1 | 44.1 | 47.4 |
| Max | 12.5 | 2.59 | 2.18 | 1.10 | 15.1 | 16.2 | 16.9 | 41.5 | 8.64 | 7.28 | 3.66 | 50.2 | 54.0 | 56.4 |
| Median | 10.5 | 2.47 | 1.55 | 0.96 | 12.9 | 14.6 | 15.5 | 34.9 | 8.24 | 5.14 | 3.20 | 43.0 | 48.7 | 51.7 |
| **Cohort 4, 90 mg (n=6)** | | | | | | | | | | | | | | |
| Ar Mean | 38.1 | 6.05 | 3.89 | 1.61 | 44.1 | 48.0 | 49.6 | 42.2 | 6.72 | 4.32 | 1.79 | 49.0 | 53.3 | 55.1 |
| CV% | 10.1 | 42.1 | 20.6 | 29.4 | 8.7 | 7.8 | 7.2 | 10.1 | 42.1 | 20.5 | 29.5 | 8.7 | 7.8 | 7.2 |
| Min | 31.2 | 1.46 | 2.93 | 1.02 | 39.5 | 43.6 | 44.9 | 34.6 | 1.62 | 3.26 | 1.13 | 43.9 | 48.5 | 49.9 |
| Max | 41.5 | 8.33 | 4.91 | 2.24 | 49.8 | 53.5 | 54.9 | 46.1 | 9.25 | 5.45 | 2.49 | 55.4 | 59.4 | 61.0 |
| Median | 39.3 | 6.36 | 4.01 | 1.50 | 44.2 | 47.8 | 49.7 | 43.6 | 7.07 | 4.46 | 1.66 | 49.1 | 53.1 | 55.3 |
| **Cohort 5, 180 mg (n=6)** | | | | | | | | | | | | | | |
| Ar Mean | 77.5 | 12.1 | 6.06 | 2.43 | 89.6 | 95.7 | 97.9 | 43.0 | 6.74 | 3.37 | 1.35 | 49.8 | 53.1 | 54.5 |
| CV% | 4.5 | 12.2 | 39.6 | 42.2 | 4.2 | 4.5 | 5.0 | 4.6 | 12.2 | 39.7 | 42.3 | 4.2 | 4.5 | 5.1 |
| Min | 73.1 | 10.3 | 3.07 | 1.26 | 84.8 | 87.9 | 89.1 | 40.6 | 5.75 | 1.70 | 0.700 | 47.1 | 48.8 | 49.5 |
| Max | 82.5 | 14.1 | 10.0 | 4.26 | 94.2 | 99.3 | 102 | 45.9 | 7.84 | 5.57 | 2.37 | 52.3 | 55.2 | 56.6 |
| Median | 77.7 | 12.1 | 5.76 | 2.20 | 89.5 | 96.8 | 99.8 | 43.2 | 6.72 | 3.20 | 1.22 | 49.7 | 53.8 | 55.6 |
| **Cohort 6, 360 mg (n=6)** | | | | | | | | | | | | | | |
| Ar Mean | 159 | 19.1 | 7.09 | 2.83 | 178 | 185 | 188 | 44.2 | 5.32 | 1.97 | 0.787 | 49.5 | 51.5 | 52.3 |
| CV% | 15.3 | 36.6 | 26.8 | 23.7 | 10.5 | 9.7 | 9.2 | 15.1 | 36.5 | 27.0 | 23.7 | 10.5 | 9.7 | 9.2 |
| Min | 136 | 8.53 | 5.49 | 1.98 | 161 | 168 | 171 | 37.8 | 2.37 | 1.53 | 0.550 | 44.7 | 46.6 | 47.5 |
| Max | 193 | 27.0 | 10.8 | 3.46 | 209 | 215 | 217 | 53.5 | 7.50 | 3.01 | 0.962 | 58.0 | 59.7 | 60.3 |
| Median | 153 | 20.0 | 6.73 | 2.89 | 172 | 178 | 182 | 42.4 | 5.55 | 1.87 | 0.803 | 47.8 | 49.5 | 50.4 |
| **Cohort 7, 720 mg (n=6)** | | | | | | | | | | | | | | |
| Ar Mean | 301 | 42.5 | 11.7 | 4.09 | 344 | 355 | 360 | 41.8 | 5.90 | 1.63 | 0.569 | 47.7 | 49.4 | 49.9 |
| CV% | 12.1 | 14.1 | 13.7 | 30.4 | 10.2 | 9.7 | 9.7 | 12.1 | 14.1 | 13.6 | 30.3 | 10.2 | 9.7 | 9.7 |
| Min | 271 | 34.5 | 9.71 | 1.94 | 309 | 318 | 320 | 37.6 | 4.79 | 1.35 | 0.270 | 42.9 | 44.2 | 44.5 |
| Max | 359 | 47.4 | 13.8 | 5.31 | 394 | 404 | 407 | 49.8 | 6.59 | 1.91 | 0.738 | 54.7 | 56.1 | 56.6 |
| Median | 285 | 45.7 | 12.1 | 4.53 | 332 | 345 | 350 | 39.6 | 6.34 | 1.68 | 0.630 | 46.1 | 47.9 | 48.5 |
| **Cohort 8, 1000 mg (n=6)** | | | | | | | | | | | | | | |
| Ar Mean | 428 | 53.7 | 16.4 | 5.21 | 482 | 498 | 504 | 42.8 | 5.37 | 1.64 | 0.521 | 48.2 | 49.8 | 50.4 |
| CV% | 15.1 | 28.4 | 32.4 | 27.1 | 13.8 | 13.2 | 13.1 | 15.1 | 28.4 | 32.4 | 27.1 | 13.8 | 13.2 | 13.1 |
| Min | 344 | 26.9 | 9.97 | 3.66 | 400 | 411 | 415 | 34.4 | 2.69 | 0.997 | 0.366 | 40.0 | 41.1 | 41.5 |
| Max | 538 | 70.7 | 24.6 | 7.67 | 602 | 612 | 617 | 53.8 | 7.07 | 2.46 | 0.767 | 60.2 | 61.2 | 61.7 |
| Median | 421 | 56.1 | 16.6 | 5.03 | 475 | 496 | 501 | 42.1 | 5.61 | 1.66 | 0.503 | 47.5 | 49.6 | 50.1 |

Ar: Arithmetic; DN: dose normalized; Geo: geometric; Max: maximum; Min: minimum.
[a] Ae_(0-last)_ and fe_(0-last)_ correspond to Ae_(0-48)_.and fe_(0-48)_, respectively_._

Source: DMID 13-0001 Phase 1 Clinical Study Report, Table 11.3; data summarized from Table 14.2.4, Table 14.2.5
