# Supplementary material for: Randomized single oral dose phase 1 study of safety, tolerability, and pharmacokinetics of Iminosugar UV-4 Hydrochloride (UV-4B) in healthy subjects
Source: PLoS Negl Trop Dis. 2022 Aug 8;16(8):e0010636. doi: 10.1371/journal.pntd.0010636 (PMC9387934; doi:10.1371/journal.pntd.0010636)
Supplement: S3 Table — (DOC) [file pntd.0010636.s003.doc]

Concentrations of UV-4 in Human Plasma Samples

(Callahan, UV4 FIH, Supplemental Information)

| SUBJECT  ALIAS ID | SUBJECT  GROUP | NOMINAL DAY | NOMINAL  HOUR | CONCENTRATION UV4 (ng/mL) |
| --- | --- | --- | --- | --- |
| 11290 | Cohort 1 | 1 | 0 | <LLOQ (0.500) |
| 11290 | Cohort 1 | 1 | 0.5 | 22.8 |
| 11290 | Cohort 1 | 1 | 1 | 19.1 |
| 11290 | Cohort 1 | 1 | 1.5 | 13.4 |
| 11290 | Cohort 1 | 1 | 2 | 8.85 |
| 11290 | Cohort 1 | 1 | 2.5 | 7.30 |
| 11290 | Cohort 1 | 1 | 3 | 6.79 |
| 11290 | Cohort 1 | 1 | 4 | 5.48 |
| 11290 | Cohort 1 | 1 | 6 | 4.39 |
| 11290 | Cohort 1 | 1 | 9 | 2.90 |
| 11290 | Cohort 1 | 1 | 12 | 2.19 |
| 11290 | Cohort 1 | 1 | 18 | 1.09 |
| 11290 | Cohort 1 | 2 | 24 | 0.961 |
| 11290 | Cohort 1 | 2 | 36 | <LLOQ (0.500) |
| 11290 | Cohort 1 | 3 | 48 | <LLOQ (0.500) |
| 17603 | Cohort 1 | 1 | 0 | <LLOQ (0.500) |
| 17603 | Cohort 1 | 1 | 0.5 | <LLOQ (0.500) |
| 17603 | Cohort 1 | 1 | 1 | <LLOQ (0.500) |
| 17603 | Cohort 1 | 1 | 1.5 | <LLOQ (0.500) |
| 17603 | Cohort 1 | 1 | 2 | <LLOQ (0.500) |
| 17603 | Cohort 1 | 1 | 2.5 | <LLOQ (0.500) |
| 17603 | Cohort 1 | 1 | 3 | <LLOQ (0.500) |
| 17603 | Cohort 1 | 1 | 4 | <LLOQ (0.500) |
| 17603 | Cohort 1 | 1 | 6 | <LLOQ (0.500) |
| 17603 | Cohort 1 | 1 | 9 | <LLOQ (0.500) |
| 17603 | Cohort 1 | 1 | 12 | <LLOQ (0.500) |
| 17603 | Cohort 1 | 1 | 18 | <LLOQ (0.500) |
| 17603 | Cohort 1 | 2 | 24 | <LLOQ (0.500) |
| 17603 | Cohort 1 | 2 | 36 | <LLOQ (0.500) |
| 17603 | Cohort 1 | 3 | 48 | <LLOQ (0.500) |
| 12126 | Cohort 1 | 1 | 0 | <LLOQ (0.500) |
| 12126 | Cohort 1 | 1 | 0.5 | 12.2 |
| 12126 | Cohort 1 | 1 | 1 | 18.4 |
| 12126 | Cohort 1 | 1 | 1.5 | 12.5 |
| 12126 | Cohort 1 | 1 | 2 | 8.26 |
| 12126 | Cohort 1 | 1 | 2.5 | 6.59 |
| 12126 | Cohort 1 | 1 | 3 | 5.78 |
| 12126 | Cohort 1 | 1 | 4 | 4.57 |
| 12126 | Cohort 1 | 1 | 6 | 3.73 |
| 12126 | Cohort 1 | 1 | 9 | 2.36 |
| 12126 | Cohort 1 | 1 | 12 | 1.69 |
| 12126 | Cohort 1 | 1 | 18 | 0.989 |
| 12126 | Cohort 1 | 2 | 24 | 0.676 |
| 12126 | Cohort 1 | 2 | 36 | <LLOQ (0.500) |
| 12126 | Cohort 1 | 3 | 48 | <LLOQ (0.500) |
| 10888 | Cohort 1 | 1 | 0 | <LLOQ (0.500) |
| 10888 | Cohort 1 | 1 | 0.5 | 20.3 |
| 10888 | Cohort 1 | 1 | 1 | 14.1 |
| 10888 | Cohort 1 | 1 | 1.5 | 10.2 |
| 10888 | Cohort 1 | 1 | 2 | 9.15 |
| 10888 | Cohort 1 | 1 | 2.5 | 10.2 |
| 10888 | Cohort 1 | 1 | 3 | 9.62 |
| 10888 | Cohort 1 | 1 | 4 | 7.93 |
| 10888 | Cohort 1 | 1 | 6 | 6.46 |
| 10888 | Cohort 1 | 1 | 9 | 3.60 |
| 10888 | Cohort 1 | 1 | 12 | 2.74 |
| 10888 | Cohort 1 | 1 | 18 | 1.33 |
| 10888 | Cohort 1 | 2 | 24 | 0.973 |
| 10888 | Cohort 1 | 2 | 36 | <LLOQ (0.500) |
| 10888 | Cohort 1 | 3 | 48 | <LLOQ (0.500) |
| 12604 | Cohort 1 | 1 | 0 | <LLOQ (0.500) |
| 12604 | Cohort 1 | 1 | 0.5 | 28.5 |
| 12604 | Cohort 1 | 1 | 1 | 16.6 |
| 12604 | Cohort 1 | 1 | 1.5 | 11.3 |
| 12604 | Cohort 1 | 1 | 2 | 8.92 |
| 12604 | Cohort 1 | 1 | 2.5 | 7.28 |
| 12604 | Cohort 1 | 1 | 3 | 6.94 |
| 12604 | Cohort 1 | 1 | 4 | 5.57 |
| 12604 | Cohort 1 | 1 | 6 | 4.69 |
| 12604 | Cohort 1 | 1 | 9 | 3.11 |
| 12604 | Cohort 1 | 1 | 12 | 2.42 |
| 12604 | Cohort 1 | 1 | 18 | 1.42 |
| 12604 | Cohort 1 | 2 | 24 | 1.05 |
| 12604 | Cohort 1 | 2 | 36 | 0.655 |
| 12604 | Cohort 1 | 3 | 48 | <LLOQ (0.500) |
| 19993 | Cohort 1 | 1 | 0 | <LLOQ (0.500) |
| 19993 | Cohort 1 | 1 | 0.5 | 23.7 |
| 19993 | Cohort 1 | 1 | 1 | 14.2 |
| 19993 | Cohort 1 | 1 | 1.5 | 7.73 |
| 19993 | Cohort 1 | 1 | 2 | 5.89 |
| 19993 | Cohort 1 | 1 | 2.5 | 4.77 |
| 19993 | Cohort 1 | 1 | 3 | 4.04 |
| 19993 | Cohort 1 | 1 | 4 | 3.14 |
| 19993 | Cohort 1 | 1 | 6 | 3.12 |
| 19993 | Cohort 1 | 1 | 9 | 1.86 |
| 19993 | Cohort 1 | 1 | 12 | 1.53 |
| 19993 | Cohort 1 | 1 | 18 | 1.02 |
| 19993 | Cohort 1 | 2 | 24 | 0.894 |
| 19993 | Cohort 1 | 2 | 36 | <LLOQ (0.500) |
| 19993 | Cohort 1 | 3 | 48 | <LLOQ (0.500) |
| 15202 | Cohort 1 | 1 | 0 | <LLOQ (0.500) |
| 15202 | Cohort 1 | 1 | 0.5 | 20.4 |
| 15202 | Cohort 1 | 1 | 1 | 19.3 |
| 15202 | Cohort 1 | 1 | 1.5 | 11.5 |
| 15202 | Cohort 1 | 1 | 2 | 8.61 |
| 15202 | Cohort 1 | 1 | 2.5 | 7.11 |
| 15202 | Cohort 1 | 1 | 3 | 5.92 |
| 15202 | Cohort 1 | 1 | 4 | 4.68 |
| 15202 | Cohort 1 | 1 | 6 | 4.07 |
| 15202 | Cohort 1 | 1 | 9 | 2.41 |
| 15202 | Cohort 1 | 1 | 12 | 1.86 |
| 15202 | Cohort 1 | 1 | 18 | 0.911 |
| 15202 | Cohort 1 | 2 | 24 | 0.697 |
| 15202 | Cohort 1 | 2 | 36 | <LLOQ (0.500) |
| 15202 | Cohort 1 | 3 | 48 | <LLOQ (0.500) |
| 16861 | Cohort 1 | 1 | 0 | <LLOQ (0.500) |
| 16861 | Cohort 1 | 1 | 0.5 | <LLOQ (0.500) |
| 16861 | Cohort 1 | 1 | 1 | <LLOQ (0.500) |
| 16861 | Cohort 1 | 1 | 1.5 | <LLOQ (0.500) |
| 16861 | Cohort 1 | 1 | 2 | <LLOQ (0.500) |
| 16861 | Cohort 1 | 1 | 2.5 | <LLOQ (0.500) |
| 16861 | Cohort 1 | 1 | 3 | <LLOQ (0.500) |
| 16861 | Cohort 1 | 1 | 4 | <LLOQ (0.500) |
| 16861 | Cohort 1 | 1 | 6 | <LLOQ (0.500) |
| 16861 | Cohort 1 | 1 | 9 | <LLOQ (0.500) |
| 16861 | Cohort 1 | 1 | 12 | <LLOQ (0.500) |
| 16861 | Cohort 1 | 1 | 18 | <LLOQ (0.500) |
| 16861 | Cohort 1 | 2 | 24 | <LLOQ (0.500) |
| 16861 | Cohort 1 | 2 | 36 | <LLOQ (0.500) |
| 16861 | Cohort 1 | 3 | 48 | <LLOQ (0.500) |
| 14981 | Cohort 2 | 1 | 0 | <LLOQ (0.500) |
| 14981 | Cohort 2 | 1 | 0.5 | <LLOQ (0.500) |
| 14981 | Cohort 2 | 1 | 1 | <LLOQ (0.500) |
| 14981 | Cohort 2 | 1 | 1.5 | <LLOQ (0.500) |
| 14981 | Cohort 2 | 1 | 2 | <LLOQ (0.500) |
| 14981 | Cohort 2 | 1 | 2.5 | <LLOQ (0.500) |
| 14981 | Cohort 2 | 1 | 3 | <LLOQ (0.500) |
| 14981 | Cohort 2 | 1 | 4 | <LLOQ (0.500) |
| 14981 | Cohort 2 | 1 | 6 | <LLOQ (0.500) |
| 14981 | Cohort 2 | 1 | 9 | <LLOQ (0.500) |
| 14981 | Cohort 2 | 1 | 12 | <LLOQ (0.500) |
| 14981 | Cohort 2 | 1 | 18 | <LLOQ (0.500) |
| 14981 | Cohort 2 | 2 | 24 | <LLOQ (0.500) |
| 14981 | Cohort 2 | 2 | 36 | <LLOQ (0.500) |
| 14981 | Cohort 2 | 3 | 48 | <LLOQ (0.500) |
| 15473 | Cohort 2 | 1 | 0 | <LLOQ (0.500) |
| 15473 | Cohort 2 | 1 | 0.5 | 60.3 |
| 15473 | Cohort 2 | 1 | 1 | 57.9 |
| 15473 | Cohort 2 | 1 | 1.5 | 40.8 |
| 15473 | Cohort 2 | 1 | 2 | 34.3 |
| 15473 | Cohort 2 | 1 | 2.5 | 28.5 |
| 15473 | Cohort 2 | 1 | 3 | 25.7 |
| 15473 | Cohort 2 | 1 | 4 | 19.3 |
| 15473 | Cohort 2 | 1 | 6 | 14.6 |
| 15473 | Cohort 2 | 1 | 9 | 10.0 |
| 15473 | Cohort 2 | 1 | 12 | 7.36 |
| 15473 | Cohort 2 | 1 | 18 | 3.53 |
| 15473 | Cohort 2 | 2 | 24 | 2.89 |
| 15473 | Cohort 2 | 2 | 36 | 1.27 |
| 15473 | Cohort 2 | 3 | 48 | 0.839 |
| 17509 | Cohort 2 | 1 | 0 | <LLOQ (0.500) |
| 17509 | Cohort 2 | 1 | 0.5 | <LLOQ (0.500) |
| 17509 | Cohort 2 | 1 | 1 | <LLOQ (0.500) |
| 17509 | Cohort 2 | 1 | 1.5 | <LLOQ (0.500) |
| 17509 | Cohort 2 | 1 | 2 | <LLOQ (0.500) |
| 17509 | Cohort 2 | 1 | 2.5 | <LLOQ (0.500) |
| 17509 | Cohort 2 | 1 | 3 | <LLOQ (0.500) |
| 17509 | Cohort 2 | 1 | 4 | <LLOQ (0.500) |
| 17509 | Cohort 2 | 1 | 6 | <LLOQ (0.500) |
| 17509 | Cohort 2 | 1 | 9 | <LLOQ (0.500) |
| 17509 | Cohort 2 | 1 | 12 | <LLOQ (0.500) |
| 17509 | Cohort 2 | 1 | 18 | <LLOQ (0.500) |
| 17509 | Cohort 2 | 2 | 24 | <LLOQ (0.500) |
| 17509 | Cohort 2 | 2 | 36 | <LLOQ (0.500) |
| 17509 | Cohort 2 | 3 | 48 | <LLOQ (0.500) |
| 18798 | Cohort 2 | 1 | 0 | <LLOQ (0.500) |
| 18798 | Cohort 2 | 1 | 0.5 | 80.9 |
| 18798 | Cohort 2 | 1 | 1 | 91.3 |
| 18798 | Cohort 2 | 1 | 1.5 | 53.4 |
| 18798 | Cohort 2 | 1 | 2 | 37.9 |
| 18798 | Cohort 2 | 1 | 2.5 | 29.2 |
| 18798 | Cohort 2 | 1 | 3 | 24.9 |
| 18798 | Cohort 2 | 1 | 4 | 19.5 |
| 18798 | Cohort 2 | 1 | 6 | 13.4 |
| 18798 | Cohort 2 | 1 | 9 | 9.06 |
| 18798 | Cohort 2 | 1 | 12 | 6.50 |
| 18798 | Cohort 2 | 1 | 18 | 3.63 |
| 18798 | Cohort 2 | 2 | 24 | 2.51 |
| 18798 | Cohort 2 | 2 | 36 | 1.10 |
| 18798 | Cohort 2 | 3 | 48 | 0.784 |
| 16510 | Cohort 2 | 1 | 0 | <LLOQ (0.500) |
| 16510 | Cohort 2 | 1 | 0.5 | 50.6 |
| 16510 | Cohort 2 | 1 | 1 | 74.6 |
| 16510 | Cohort 2 | 1 | 1.5 | 60.0 |
| 16510 | Cohort 2 | 1 | 2 | 51.4 |
| 16510 | Cohort 2 | 1 | 2.5 | 35.4 |
| 16510 | Cohort 2 | 1 | 3 | 30.3 |
| 16510 | Cohort 2 | 1 | 4 | 22.8 |
| 16510 | Cohort 2 | 1 | 6 | 18.1 |
| 16510 | Cohort 2 | 1 | 9 | 11.1 |
| 16510 | Cohort 2 | 1 | 12 | 7.22 |
| 16510 | Cohort 2 | 1 | 18 | 4.07 |
| 16510 | Cohort 2 | 2 | 24 | 2.72 |
| 16510 | Cohort 2 | 2 | 36 | 1.06 |
| 16510 | Cohort 2 | 3 | 48 | 0.648 |
| 12612 | Cohort 2 | 1 | 0 | <LLOQ (0.500) |
| 12612 | Cohort 2 | 1 | 0.5 | 107 |
| 12612 | Cohort 2 | 1 | 1 | 99.9 |
| 12612 | Cohort 2 | 1 | 1.5 | 60.5 |
| 12612 | Cohort 2 | 1 | 2 | 51.2 |
| 12612 | Cohort 2 | 1 | 2.5 | 40.9 |
| 12612 | Cohort 2 | 1 | 3 | 35.3 |
| 12612 | Cohort 2 | 1 | 4 | 27.4 |
| 12612 | Cohort 2 | 1 | 6 | 19.0 |
| 12612 | Cohort 2 | 1 | 9 | 12.5 |
| 12612 | Cohort 2 | 1 | 12 | 7.98 |
| 12612 | Cohort 2 | 1 | 18 | 4.05 |
| 12612 | Cohort 2 | 2 | 24 | 3.74 |
| 12612 | Cohort 2 | 2 | 36 | 1.39 |
| 12612 | Cohort 2 | 3 | 48 | 0.787 |
| 15239 | Cohort 2 | 1 | 0 | <LLOQ (0.500) |
| 15239 | Cohort 2 | 1 | 0.5 | 21.2 |
| 15239 | Cohort 2 | 1 | 1 | 68.2 |
| 15239 | Cohort 2 | 1 | 1.5 | 51.4 |
| 15239 | Cohort 2 | 1 | 2 | 58.4 |
| 15239 | Cohort 2 | 1 | 2.5 | 52.2 |
| 15239 | Cohort 2 | 1 | 3 | 41.6 |
| 15239 | Cohort 2 | 1 | 4 | 33.8 |
| 15239 | Cohort 2 | 1 | 6 | 23.6 |
| 15239 | Cohort 2 | 1 | 9 | 14.1 |
| 15239 | Cohort 2 | 1 | 12 | 8.85 |
| 15239 | Cohort 2 | 1 | 18 | 3.69 |
| 15239 | Cohort 2 | 2 | 24 | 2.56 |
| 15239 | Cohort 2 | 2 | 36 | 0.959 |
| 15239 | Cohort 2 | 3 | 48 | 0.553 |
| 15090 | Cohort 2 | 1 | 0 | <LLOQ (0.500) |
| 15090 | Cohort 2 | 1 | 0.5 | 65.5 |
| 15090 | Cohort 2 | 1 | 1 | 97.6 |
| 15090 | Cohort 2 | 1 | 1.5 | 106 |
| 15090 | Cohort 2 | 1 | 2 | 62.5 |
| 15090 | Cohort 2 | 1 | 2.5 | 53.3 |
| 15090 | Cohort 2 | 1 | 3 | 50.2 |
| 15090 | Cohort 2 | 1 | 4 | 43.2 |
| 15090 | Cohort 2 | 1 | 6 | 34.9 |
| 15090 | Cohort 2 | 1 | 9 | 23.7 |
| 15090 | Cohort 2 | 1 | 12 | 15.3 |
| 15090 | Cohort 2 | 1 | 18 | 7.08 |
| 15090 | Cohort 2 | 2 | 24 | 4.80 |
| 15090 | Cohort 2 | 2 | 36 | 2.22 |
| 15090 | Cohort 2 | 3 | 48 | 1.15 |
| 19949 | Cohort 3 | 1 | 0 | <LLOQ (0.500) |
| 19949 | Cohort 3 | 1 | 0.5 | 295 |
| 19949 | Cohort 3 | 1 | 1 | 305 |
| 19949 | Cohort 3 | 1 | 1.5 | 214 |
| 19949 | Cohort 3 | 1 | 2 | 168 |
| 19949 | Cohort 3 | 1 | 2.5 | 128 |
| 19949 | Cohort 3 | 1 | 3 | 118 |
| 19949 | Cohort 3 | 1 | 4 | 85.8 |
| 19949 | Cohort 3 | 1 | 6 | 61.0 |
| 19949 | Cohort 3 | 1 | 9 | 34.9 |
| 19949 | Cohort 3 | 1 | 12 | 24.7 |
| 19949 | Cohort 3 | 1 | 18 | 11.6 |
| 19949 | Cohort 3 | 2 | 24 | 7.87 |
| 19949 | Cohort 3 | 2 | 36 | 2.83 |
| 19949 | Cohort 3 | 3 | 48 | 1.45 |
| 17096 | Cohort 3 | 1 | 0 | <LLOQ (0.500) |
| 17096 | Cohort 3 | 1 | 0.5 | <LLOQ (0.500) |
| 17096 | Cohort 3 | 1 | 1 | <LLOQ (0.500) |
| 17096 | Cohort 3 | 1 | 1.5 | <LLOQ (0.500) |
| 17096 | Cohort 3 | 1 | 2 | <LLOQ (0.500) |
| 17096 | Cohort 3 | 1 | 2.5 | <LLOQ (0.500) |
| 17096 | Cohort 3 | 1 | 3 | <LLOQ (0.500) |
| 17096 | Cohort 3 | 1 | 4 | <LLOQ (0.500) |
| 17096 | Cohort 3 | 1 | 6 | <LLOQ (0.500) |
| 17096 | Cohort 3 | 1 | 9 | <LLOQ (0.500) |
| 17096 | Cohort 3 | 1 | 12 | <LLOQ (0.500) |
| 17096 | Cohort 3 | 1 | 18 | <LLOQ (0.500) |
| 17096 | Cohort 3 | 2 | 24 | <LLOQ (0.500) |
| 17096 | Cohort 3 | 2 | 36 | <LLOQ (0.500) |
| 17096 | Cohort 3 | 3 | 48 | <LLOQ (0.500) |
| 18776 | Cohort 3 | 1 | 0 | <LLOQ (0.500) |
| 18776 | Cohort 3 | 1 | 0.5 | 307 |
| 18776 | Cohort 3 | 1 | 1 | 244 |
| 18776 | Cohort 3 | 1 | 1.5 | 227 |
| 18776 | Cohort 3 | 1 | 2 | 212 |
| 18776 | Cohort 3 | 1 | 2.5 | 171 |
| 18776 | Cohort 3 | 1 | 3 | 135 |
| 18776 | Cohort 3 | 1 | 4 | 115 |
| 18776 | Cohort 3 | 1 | 6 | 73.6 |
| 18776 | Cohort 3 | 1 | 9 | 37.1 |
| 18776 | Cohort 3 | 1 | 12 | 23.4 |
| 18776 | Cohort 3 | 1 | 18 | 9.38 |
| 18776 | Cohort 3 | 2 | 24 | 5.74 |
| 18776 | Cohort 3 | 2 | 36 | 1.82 |
| 18776 | Cohort 3 | 3 | 48 | 0.929 |
| 14041 | Cohort 3 | 1 | 0 | <LLOQ (0.500) |
| 14041 | Cohort 3 | 1 | 0.5 | 205 |
| 14041 | Cohort 3 | 1 | 1 | 174 |
| 14041 | Cohort 3 | 1 | 1.5 | 178 |
| 14041 | Cohort 3 | 1 | 2 | 188 |
| 14041 | Cohort 3 | 1 | 2.5 | 176 |
| 14041 | Cohort 3 | 1 | 3 | 161 |
| 14041 | Cohort 3 | 1 | 4 | 123 |
| 14041 | Cohort 3 | 1 | 6 | 70.1 |
| 14041 | Cohort 3 | 1 | 9 | 34.5 |
| 14041 | Cohort 3 | 1 | 12 | 21.2 |
| 14041 | Cohort 3 | 1 | 18 | 8.52 |
| 14041 | Cohort 3 | 2 | 24 | 5.53 |
| 14041 | Cohort 3 | 2 | 36 | 1.86 |
| 14041 | Cohort 3 | 3 | 48 | 1.13 |
| 17172 | Cohort 3 | 1 | 0 | <LLOQ (0.500) |
| 17172 | Cohort 3 | 1 | 0.5 | <LLOQ (0.500) |
| 17172 | Cohort 3 | 1 | 1 | <LLOQ (0.500) |
| 17172 | Cohort 3 | 1 | 1.5 | <LLOQ (0.500) |
| 17172 | Cohort 3 | 1 | 2 | <LLOQ (0.500) |
| 17172 | Cohort 3 | 1 | 2.5 | <LLOQ (0.500) |
| 17172 | Cohort 3 | 1 | 3 | <LLOQ (0.500) |
| 17172 | Cohort 3 | 1 | 4 | <LLOQ (0.500) |
| 17172 | Cohort 3 | 1 | 6 | <LLOQ (0.500) |
| 17172 | Cohort 3 | 1 | 9 | <LLOQ (0.500) |
| 17172 | Cohort 3 | 1 | 12 | <LLOQ (0.500) |
| 17172 | Cohort 3 | 1 | 18 | <LLOQ (0.500) |
| 17172 | Cohort 3 | 2 | 24 | <LLOQ (0.500) |
| 17172 | Cohort 3 | 2 | 36 | <LLOQ (0.500) |
| 17172 | Cohort 3 | 3 | 48 | <LLOQ (0.500) |
| 14265 | Cohort 3 | 1 | 0 | <LLOQ (0.500) |
| 14265 | Cohort 3 | 1 | 0.5 | 317 |
| 14265 | Cohort 3 | 1 | 1 | 275 |
| 14265 | Cohort 3 | 1 | 1.5 | 179 |
| 14265 | Cohort 3 | 1 | 2 | 168 |
| 14265 | Cohort 3 | 1 | 2.5 | 131 |
| 14265 | Cohort 3 | 1 | 3 | 111 |
| 14265 | Cohort 3 | 1 | 4 | 75.4 |
| 14265 | Cohort 3 | 1 | 6 | 58.3 |
| 14265 | Cohort 3 | 1 | 9 | 38.3 |
| 14265 | Cohort 3 | 1 | 12 | 22.0 |
| 14265 | Cohort 3 | 1 | 18 | 10.7 |
| 14265 | Cohort 3 | 2 | 24 | 7.50 |
| 14265 | Cohort 3 | 2 | 36 | 2.77 |
| 14265 | Cohort 3 | 3 | 48 | 1.42 |
| 11117 | Cohort 3 | 1 | 0 | <LLOQ (0.500) |
| 11117 | Cohort 3 | 1 | 0.5 | 330 |
| 11117 | Cohort 3 | 1 | 1 | 303 |
| 11117 | Cohort 3 | 1 | 1.5 | 160 |
| 11117 | Cohort 3 | 1 | 2 | 111 |
| 11117 | Cohort 3 | 1 | 2.5 | 85.2 |
| 11117 | Cohort 3 | 1 | 3 | 70.0 |
| 11117 | Cohort 3 | 1 | 4 | 51.8 |
| 11117 | Cohort 3 | 1 | 6 | 41.6 |
| 11117 | Cohort 3 | 1 | 9 | 25.1 |
| 11117 | Cohort 3 | 1 | 12 | 16.9 |
| 11117 | Cohort 3 | 1 | 18 | 8.41 |
| 11117 | Cohort 3 | 2 | 24 | 6.14 |
| 11117 | Cohort 3 | 2 | 36 | 2.30 |
| 11117 | Cohort 3 | 3 | 48 | 1.32 |
| 16824 | Cohort 3 | 1 | 0 | <LLOQ (0.500) |
| 16824 | Cohort 3 | 1 | 0.5 | 291 |
| 16824 | Cohort 3 | 1 | 1 | 240 |
| 16824 | Cohort 3 | 1 | 1.5 | 153 |
| 16824 | Cohort 3 | 1 | 2 | 147 |
| 16824 | Cohort 3 | 1 | 2.5 | 125 |
| 16824 | Cohort 3 | 1 | 3 | 116 |
| 16824 | Cohort 3 | 1 | 4 | 98.6 |
| 16824 | Cohort 3 | 1 | 6 | 61.8 |
| 16824 | Cohort 3 | 1 | 9 | 34.8 |
| 16824 | Cohort 3 | 1 | 12 | 26.7 |
| 16824 | Cohort 3 | 1 | 18 | 11.7 |
| 16824 | Cohort 3 | 2 | 24 | 8.26 |
| 16824 | Cohort 3 | 2 | 36 | 3.40 |
| 16824 | Cohort 3 | 3 | 48 | 1.77 |
| 14776 | Cohort 4 | 1 | 0 | <LLOQ (0.500) |
| 14776 | Cohort 4 | 1 | 0.5 | <LLOQ (0.500) |
| 14776 | Cohort 4 | 1 | 1 | <LLOQ (0.500) |
| 14776 | Cohort 4 | 1 | 1.5 | <LLOQ (0.500) |
| 14776 | Cohort 4 | 1 | 2 | <LLOQ (0.500) |
| 14776 | Cohort 4 | 1 | 2.5 | <LLOQ (0.500) |
| 14776 | Cohort 4 | 1 | 3 | <LLOQ (0.500) |
| 14776 | Cohort 4 | 1 | 4 | <LLOQ (0.500) |
| 14776 | Cohort 4 | 1 | 6 | <LLOQ (0.500) |
| 14776 | Cohort 4 | 1 | 9 | <LLOQ (0.500) |
| 14776 | Cohort 4 | 1 | 12 | <LLOQ (0.500) |
| 14776 | Cohort 4 | 1 | 18 | <LLOQ (0.500) |
| 14776 | Cohort 4 | 2 | 24 | <LLOQ (0.500) |
| 14776 | Cohort 4 | 2 | 36 | <LLOQ (0.500) |
| 14776 | Cohort 4 | 3 | 48 | <LLOQ (0.500) |
| 13523 | Cohort 4 | 1 | 0 | <LLOQ (0.500) |
| 13523 | Cohort 4 | 1 | 0.5 | 439 |
| 13523 | Cohort 4 | 1 | 1 | 589 |
| 13523 | Cohort 4 | 1 | 1.5 | 754 |
| 13523 | Cohort 4 | 1 | 2 | 583 |
| 13523 | Cohort 4 | 1 | 2.5 | 516 |
| 13523 | Cohort 4 | 1 | 3 | 495 |
| 13523 | Cohort 4 | 1 | 4 | 368 |
| 13523 | Cohort 4 | 1 | 6 | 184 |
| 13523 | Cohort 4 | 1 | 9 | 101 |
| 13523 | Cohort 4 | 1 | 12 | 54.3 |
| 13523 | Cohort 4 | 1 | 18 | 18.1 |
| 13523 | Cohort 4 | 2 | 24 | 12.5 |
| 13523 | Cohort 4 | 2 | 36 | 4.31 |
| 13523 | Cohort 4 | 3 | 48 | 2.04 |
| 12146 | Cohort 4 | 1 | 0 | 0.569 |
| 12146 | Cohort 4 | 1 | 0.5 | 598 |
| 12146 | Cohort 4 | 1 | 1 | 571 |
| 12146 | Cohort 4 | 1 | 1.5 | 487 |
| 12146 | Cohort 4 | 1 | 2 | 406 |
| 12146 | Cohort 4 | 1 | 2.5 | 439 |
| 12146 | Cohort 4 | 1 | 3 | 428 |
| 12146 | Cohort 4 | 1 | 4 | 298 |
| 12146 | Cohort 4 | 1 | 6 | 187 |
| 12146 | Cohort 4 | 1 | 9 | 118 |
| 12146 | Cohort 4 | 1 | 12 | 66.4 |
| 12146 | Cohort 4 | 1 | 18 | 34.0 |
| 12146 | Cohort 4 | 2 | 24 | 26.6 |
| 12146 | Cohort 4 | 2 | 36 | 11.5 |
| 12146 | Cohort 4 | 3 | 48 | 4.82 |
| 11798 | Cohort 4 | 1 | 0 | 0.657 |
| 11798 | Cohort 4 | 1 | 0.5 | <LLOQ (0.500) |
| 11798 | Cohort 4 | 1 | 1 | <LLOQ (0.500) |
| 11798 | Cohort 4 | 1 | 1.5 | <LLOQ (0.500) |
| 11798 | Cohort 4 | 1 | 2 | <LLOQ (0.500) |
| 11798 | Cohort 4 | 1 | 2.5 | <LLOQ (0.500) |
| 11798 | Cohort 4 | 1 | 3 | <LLOQ (0.500) |
| 11798 | Cohort 4 | 1 | 4 | <LLOQ (0.500) |
| 11798 | Cohort 4 | 1 | 6 | <LLOQ (0.500) |
| 11798 | Cohort 4 | 1 | 9 | <LLOQ (0.500) |
| 11798 | Cohort 4 | 1 | 12 | <LLOQ (0.500) |
| 11798 | Cohort 4 | 1 | 18 | <LLOQ (0.500) |
| 11798 | Cohort 4 | 2 | 24 | <LLOQ (0.500) |
| 11798 | Cohort 4 | 2 | 36 | <LLOQ (0.500) |
| 11798 | Cohort 4 | 3 | 48 | <LLOQ (0.500) |
| 10291 | Cohort 4 | 1 | 0 | 1.02 |
| 10291 | Cohort 4 | 1 | 0.5 | 942 |
| 10291 | Cohort 4 | 1 | 1 | 858 |
| 10291 | Cohort 4 | 1 | 1.5 | 860 |
| 10291 | Cohort 4 | 1 | 2 | 636 |
| 10291 | Cohort 4 | 1 | 2.5 | 447 |
| 10291 | Cohort 4 | 1 | 3 | 393 |
| 10291 | Cohort 4 | 1 | 4 | 302 |
| 10291 | Cohort 4 | 1 | 6 | 216 |
| 10291 | Cohort 4 | 1 | 9 | 128 |
| 10291 | Cohort 4 | 1 | 12 | 79.7 |
| 10291 | Cohort 4 | 1 | 18 | 43.3 |
| 10291 | Cohort 4 | 2 | 24 | 30.8 |
| 10291 | Cohort 4 | 2 | 36 | 12.0 |
| 10291 | Cohort 4 | 3 | 48 | 6.34 |
| 19130 | Cohort 4 | 1 | 0 | <LLOQ (0.500) |
| 19130 | Cohort 4 | 1 | 0.5 | 1250 |
| 19130 | Cohort 4 | 1 | 1 | 762 |
| 19130 | Cohort 4 | 1 | 1.5 | 621 |
| 19130 | Cohort 4 | 1 | 2 | 479 |
| 19130 | Cohort 4 | 1 | 2.5 | 365 |
| 19130 | Cohort 4 | 1 | 3 | 327 |
| 19130 | Cohort 4 | 1 | 4 | 236 |
| 19130 | Cohort 4 | 1 | 6 | 141 |
| 19130 | Cohort 4 | 1 | 9 | 70.4 |
| 19130 | Cohort 4 | 1 | 12 | 42.1 |
| 19130 | Cohort 4 | 1 | 18 | 16.6 |
| 19130 | Cohort 4 | 2 | 24 | 10.2 |
| 19130 | Cohort 4 | 2 | 36 | 3.22 |
| 19130 | Cohort 4 | 3 | 48 | 1.52 |
| 14697 | Cohort 4 | 1 | 0 | <LLOQ (0.500) |
| 14697 | Cohort 4 | 1 | 0.5 | 160 |
| 14697 | Cohort 4 | 1 | 1 | 642 |
| 14697 | Cohort 4 | 1 | 1.5 | 699 |
| 14697 | Cohort 4 | 1 | 2 | 610 |
| 14697 | Cohort 4 | 1 | 2.5 | 519 |
| 14697 | Cohort 4 | 1 | 3 | 437 |
| 14697 | Cohort 4 | 1 | 4 | 338 |
| 14697 | Cohort 4 | 1 | 6 | 189 |
| 14697 | Cohort 4 | 1 | 9 | 77.3 |
| 14697 | Cohort 4 | 1 | 12 | 37.6 |
| 14697 | Cohort 4 | 1 | 18 | 16.5 |
| 14697 | Cohort 4 | 2 | 24 | 9.14 |
| 14697 | Cohort 4 | 2 | 36 | 3.03 |
| 14697 | Cohort 4 | 3 | 48 | 1.46 |
| 10007 | Cohort 4 | 1 | 0 | <LLOQ (0.500) |
| 10007 | Cohort 4 | 1 | 0.5 | 1430 |
| 10007 | Cohort 4 | 1 | 1 | 930 |
| 10007 | Cohort 4 | 1 | 1.5 | 670 |
| 10007 | Cohort 4 | 1 | 2 | 496 |
| 10007 | Cohort 4 | 1 | 2.5 | 401 |
| 10007 | Cohort 4 | 1 | 3 | 333 |
| 10007 | Cohort 4 | 1 | 4 | 250 |
| 10007 | Cohort 4 | 1 | 6 | 157 |
| 10007 | Cohort 4 | 1 | 9 | 75.9 |
| 10007 | Cohort 4 | 1 | 12 | 47.0 |
| 10007 | Cohort 4 | 1 | 18 | 20.8 |
| 10007 | Cohort 4 | 2 | 24 | 12.4 |
| 10007 | Cohort 4 | 2 | 36 | 5.41 |
| 10007 | Cohort 4 | 3 | 48 | 5.90 |
| 11471 | Cohort 5 | 1 | 0 | <LLOQ (0.500) |
| 11471 | Cohort 5 | 1 | 0.5 | <LLOQ (0.500) |
| 11471 | Cohort 5 | 1 | 1 | <LLOQ (0.500) |
| 11471 | Cohort 5 | 1 | 1.5 | <LLOQ (0.500) |
| 11471 | Cohort 5 | 1 | 2 | <LLOQ (0.500) |
| 11471 | Cohort 5 | 1 | 2.5 | <LLOQ (0.500) |
| 11471 | Cohort 5 | 1 | 3 | <LLOQ (0.500) |
| 11471 | Cohort 5 | 1 | 4 | <LLOQ (0.500) |
| 11471 | Cohort 5 | 1 | 6 | <LLOQ (0.500) |
| 11471 | Cohort 5 | 1 | 9 | <LLOQ (0.500) |
| 11471 | Cohort 5 | 1 | 12 | <LLOQ (0.500) |
| 11471 | Cohort 5 | 1 | 18 | <LLOQ (0.500) |
| 11471 | Cohort 5 | 2 | 24 | <LLOQ (0.500) |
| 11471 | Cohort 5 | 2 | 36 | <LLOQ (0.500) |
| 11471 | Cohort 5 | 3 | 48 | <LLOQ (0.500) |
| 10742 | Cohort 5 | 1 | 0 | <LLOQ (0.500) |
| 10742 | Cohort 5 | 1 | 0.5 | 2730 |
| 10742 | Cohort 5 | 1 | 1 | 1900 |
| 10742 | Cohort 5 | 1 | 1.5 | 1380 |
| 10742 | Cohort 5 | 1 | 2 | 1170 |
| 10742 | Cohort 5 | 1 | 2.5 | 832 |
| 10742 | Cohort 5 | 1 | 3 | 691 |
| 10742 | Cohort 5 | 1 | 4 | 429 |
| 10742 | Cohort 5 | 1 | 6 | 221 |
| 10742 | Cohort 5 | 1 | 9 | 116 |
| 10742 | Cohort 5 | 1 | 12 | 56.5 |
| 10742 | Cohort 5 | 1 | 18 | 23.6 |
| 10742 | Cohort 5 | 2 | 24 | 13.8 |
| 10742 | Cohort 5 | 2 | 36 | 4.18 |
| 10742 | Cohort 5 | 3 | 48 | 2.15 |
| 12717 | Cohort 5 | 1 | 0 | <LLOQ (0.500) |
| 12717 | Cohort 5 | 1 | 0.5 | 1260 |
| 12717 | Cohort 5 | 1 | 1 | 1920 |
| 12717 | Cohort 5 | 1 | 1.5 | 1480 |
| 12717 | Cohort 5 | 1 | 2 | 1150 |
| 12717 | Cohort 5 | 1 | 2.5 | 1020 |
| 12717 | Cohort 5 | 1 | 3 | 1090 |
| 12717 | Cohort 5 | 1 | 4 | 828 |
| 12717 | Cohort 5 | 1 | 6 | 463 |
| 12717 | Cohort 5 | 1 | 9 | 153 |
| 12717 | Cohort 5 | 1 | 12 | 78.1 |
| 12717 | Cohort 5 | 1 | 18 | 19.0 |
| 12717 | Cohort 5 | 2 | 24 | 12.2 |
| 12717 | Cohort 5 | 2 | 36 | 3.72 |
| 12717 | Cohort 5 | 3 | 48 | 1.43 |
| 19239 | Cohort 5 | 1 | 0 | <LLOQ (0.500) |
| 19239 | Cohort 5 | 1 | 0.5 | 1140 |
| 19239 | Cohort 5 | 1 | 1 | 2770 |
| 19239 | Cohort 5 | 1 | 1.5 | 1470 |
| 19239 | Cohort 5 | 1 | 2 | 1160 |
| 19239 | Cohort 5 | 1 | 2.5 | 957 |
| 19239 | Cohort 5 | 1 | 3 | 918 |
| 19239 | Cohort 5 | 1 | 4 | 569 |
| 19239 | Cohort 5 | 1 | 6 | 320 |
| 19239 | Cohort 5 | 1 | 9 | 151 |
| 19239 | Cohort 5 | 1 | 12 | 83.0 |
| 19239 | Cohort 5 | 1 | 18 | 32.4 |
| 19239 | Cohort 5 | 2 | 24 | 18.7 |
| 19239 | Cohort 5 | 2 | 36 | 6.52 |
| 19239 | Cohort 5 | 3 | 48 | 2.85 |
| 14482 | Cohort 5 | 1 | 0 | <LLOQ (0.500) |
| 14482 | Cohort 5 | 1 | 0.5 | 929 |
| 14482 | Cohort 5 | 1 | 1 | 2450 |
| 14482 | Cohort 5 | 1 | 1.5 | 1820 |
| 14482 | Cohort 5 | 1 | 2 | 1480 |
| 14482 | Cohort 5 | 1 | 2.5 | 1180 |
| 14482 | Cohort 5 | 1 | 3 | 1160 |
| 14482 | Cohort 5 | 1 | 4 | 673 |
| 14482 | Cohort 5 | 1 | 6 | 373 |
| 14482 | Cohort 5 | 1 | 9 | 155 |
| 14482 | Cohort 5 | 1 | 12 | 77.3 |
| 14482 | Cohort 5 | 1 | 18 | 36.7 |
| 14482 | Cohort 5 | 2 | 24 | 18.3 |
| 14482 | Cohort 5 | 2 | 36 | 5.39 |
| 14482 | Cohort 5 | 3 | 48 | 2.45 |
| 19884 | Cohort 5 | 1 | 0 | <LLOQ (0.500) |
| 19884 | Cohort 5 | 1 | 0.5 | <LLOQ (0.500) |
| 19884 | Cohort 5 | 1 | 1 | <LLOQ (0.500) |
| 19884 | Cohort 5 | 1 | 1.5 | <LLOQ (0.500) |
| 19884 | Cohort 5 | 1 | 2 | <LLOQ (0.500) |
| 19884 | Cohort 5 | 1 | 2.5 | <LLOQ (0.500) |
| 19884 | Cohort 5 | 1 | 3 | <LLOQ (0.500) |
| 19884 | Cohort 5 | 1 | 4 | <LLOQ (0.500) |
| 19884 | Cohort 5 | 1 | 6 | <LLOQ (0.500) |
| 19884 | Cohort 5 | 1 | 9 | <LLOQ (0.500) |
| 19884 | Cohort 5 | 1 | 12 | <LLOQ (0.500) |
| 19884 | Cohort 5 | 1 | 18 | <LLOQ (0.500) |
| 19884 | Cohort 5 | 2 | 24 | <LLOQ (0.500) |
| 19884 | Cohort 5 | 2 | 36 | <LLOQ (0.500) |
| 19884 | Cohort 5 | 3 | 48 | <LLOQ (0.500) |
| 10558 | Cohort 5 | 1 | 0 | <LLOQ (0.500) |
| 10558 | Cohort 5 | 1 | 0.5 | 1170 |
| 10558 | Cohort 5 | 1 | 1 | 836 |
| 10558 | Cohort 5 | 1 | 1.5 | 909 |
| 10558 | Cohort 5 | 1 | 2 | 1020 |
| 10558 | Cohort 5 | 1 | 2.5 | 1080 |
| 10558 | Cohort 5 | 1 | 3 | 825 |
| 10558 | Cohort 5 | 1 | 4 | 679 |
| 10558 | Cohort 5 | 1 | 6 | 408 |
| 10558 | Cohort 5 | 1 | 9 | 231 |
| 10558 | Cohort 5 | 1 | 12 | 119 |
| 10558 | Cohort 5 | 1 | 18 | 43.9 |
| 10558 | Cohort 5 | 2 | 24 | 31.3 |
| 10558 | Cohort 5 | 2 | 36 | 11.9 |
| 10558 | Cohort 5 | 3 | 48 | 6.14 |
| 12639 | Cohort 5 | 1 | 0 | <LLOQ (0.500) |
| 12639 | Cohort 5 | 1 | 0.5 | 1850 |
| 12639 | Cohort 5 | 1 | 1 | 1730 |
| 12639 | Cohort 5 | 1 | 1.5 | 1230 |
| 12639 | Cohort 5 | 1 | 2 | 887 |
| 12639 | Cohort 5 | 1 | 2.5 | 750 |
| 12639 | Cohort 5 | 1 | 3 | 638 |
| 12639 | Cohort 5 | 1 | 4 | 637 |
| 12639 | Cohort 5 | 1 | 6 | 277 |
| 12639 | Cohort 5 | 1 | 9 | 121 |
| 12639 | Cohort 5 | 1 | 12 | 72.0 |
| 12639 | Cohort 5 | 1 | 18 | 31.1 |
| 12639 | Cohort 5 | 2 | 24 | 16.1 |
| 12639 | Cohort 5 | 2 | 36 | 5.78 |
| 12639 | Cohort 5 | 3 | 48 | 2.83 |
| 13778 | Cohort 6 | 1 | 0 | <LLOQ (0.500) |
| 13778 | Cohort 6 | 1 | 0.5 | <LLOQ (0.500) |
| 13778 | Cohort 6 | 1 | 1 | <LLOQ (0.500) |
| 13778 | Cohort 6 | 1 | 1.5 | <LLOQ (0.500) |
| 13778 | Cohort 6 | 1 | 2 | <LLOQ (0.500) |
| 13778 | Cohort 6 | 1 | 2.5 | <LLOQ (0.500) |
| 13778 | Cohort 6 | 1 | 3 | <LLOQ (0.500) |
| 13778 | Cohort 6 | 1 | 4 | <LLOQ (0.500) |
| 13778 | Cohort 6 | 1 | 6 | <LLOQ (0.500) |
| 13778 | Cohort 6 | 1 | 9 | <LLOQ (0.500) |
| 13778 | Cohort 6 | 1 | 12 | <LLOQ (0.500) |
| 13778 | Cohort 6 | 1 | 18 | <LLOQ (0.500) |
| 13778 | Cohort 6 | 2 | 24 | <LLOQ (0.500) |
| 13778 | Cohort 6 | 2 | 36 | <LLOQ (0.500) |
| 13778 | Cohort 6 | 3 | 48 | <LLOQ (0.500) |
| 15328 | Cohort 6 | 1 | 0 | <LLOQ (0.500) |
| 15328 | Cohort 6 | 1 | 0.5 | 3900 |
| 15328 | Cohort 6 | 1 | 1 | 3610 |
| 15328 | Cohort 6 | 1 | 1.5 | 3100 |
| 15328 | Cohort 6 | 1 | 2 | 2750 |
| 15328 | Cohort 6 | 1 | 2.5 | 2060 |
| 15328 | Cohort 6 | 1 | 3 | 1680 |
| 15328 | Cohort 6 | 1 | 4 | 1160 |
| 15328 | Cohort 6 | 1 | 6 | 586 |
| 15328 | Cohort 6 | 1 | 9 | 218 |
| 15328 | Cohort 6 | 1 | 12 | 91.4 |
| 15328 | Cohort 6 | 1 | 18 | 38.9 |
| 15328 | Cohort 6 | 2 | 24 | 22.0 |
| 15328 | Cohort 6 | 2 | 36 | 9.14 |
| 15328 | Cohort 6 | 3 | 48 | 2.75 |
| 19876 | Cohort 6 | 1 | 0 | <LLOQ (0.500) |
| 19876 | Cohort 6 | 1 | 0.5 | 4820 |
| 19876 | Cohort 6 | 1 | 1 | 3080 |
| 19876 | Cohort 6 | 1 | 1.5 | 2310 |
| 19876 | Cohort 6 | 1 | 2 | 1920 |
| 19876 | Cohort 6 | 1 | 2.5 | 1520 |
| 19876 | Cohort 6 | 1 | 3 | 1460 |
| 19876 | Cohort 6 | 1 | 4 | 1110 |
| 19876 | Cohort 6 | 1 | 6 | 449 |
| 19876 | Cohort 6 | 1 | 9 | 202 |
| 19876 | Cohort 6 | 1 | 12 | 122 |
| 19876 | Cohort 6 | 1 | 18 | 32.8 |
| 19876 | Cohort 6 | 2 | 24 | 16.6 |
| 19876 | Cohort 6 | 2 | 36 | 5.97 |
| 19876 | Cohort 6 | 3 | 48 | 2.21 |
| 12062 | Cohort 6 | 1 | 0 | <LLOQ (0.500) |
| 12062 | Cohort 6 | 1 | 0.5 | 5980 |
| 12062 | Cohort 6 | 1 | 1 | 4260 |
| 12062 | Cohort 6 | 1 | 1.5 | 3440 |
| 12062 | Cohort 6 | 1 | 2 | 2730 |
| 12062 | Cohort 6 | 1 | 2.5 | 2040 |
| 12062 | Cohort 6 | 1 | 3 | 1780 |
| 12062 | Cohort 6 | 1 | 4 | 1190 |
| 12062 | Cohort 6 | 1 | 6 | 584 |
| 12062 | Cohort 6 | 1 | 9 | 262 |
| 12062 | Cohort 6 | 1 | 12 | 143 |
| 12062 | Cohort 6 | 1 | 18 | 48.4 |
| 12062 | Cohort 6 | 2 | 24 | 34.9 |
| 12062 | Cohort 6 | 2 | 36 | 10.9 |
| 12062 | Cohort 6 | 3 | 48 | 5.17 |
| 18453 | Cohort 6 | 1 | 0 | <LLOQ (0.500) |
| 18453 | Cohort 6 | 1 | 0.5 | 4840 |
| 18453 | Cohort 6 | 1 | 1 | 3980 |
| 18453 | Cohort 6 | 1 | 1.5 | 2570 |
| 18453 | Cohort 6 | 1 | 2 | 2330 |
| 18453 | Cohort 6 | 1 | 2.5 | 2020 |
| 18453 | Cohort 6 | 1 | 3 | 1620 |
| 18453 | Cohort 6 | 1 | 4 | 1420 |
| 18453 | Cohort 6 | 1 | 6 | 682 |
| 18453 | Cohort 6 | 1 | 9 | 289 |
| 18453 | Cohort 6 | 1 | 12 | 129 |
| 18453 | Cohort 6 | 1 | 18 | 53.5 |
| 18453 | Cohort 6 | 2 | 24 | 38.5 |
| 18453 | Cohort 6 | 2 | 36 | 11.8 |
| 18453 | Cohort 6 | 3 | 48 | 5.95 |
| 19374 | Cohort 6 | 1 | 0 | <LLOQ (0.500) |
| 19374 | Cohort 6 | 1 | 0.5 | 4510 |
| 19374 | Cohort 6 | 1 | 1 | 3810 |
| 19374 | Cohort 6 | 1 | 1.5 | 2500 |
| 19374 | Cohort 6 | 1 | 2 | 1850 |
| 19374 | Cohort 6 | 1 | 2.5 | 1400 |
| 19374 | Cohort 6 | 1 | 3 | 1090 |
| 19374 | Cohort 6 | 1 | 4 | 811 |
| 19374 | Cohort 6 | 1 | 6 | 397 |
| 19374 | Cohort 6 | 1 | 9 | 161 |
| 19374 | Cohort 6 | 1 | 12 | 84.1 |
| 19374 | Cohort 6 | 1 | 18 | 22.1 |
| 19374 | Cohort 6 | 2 | 24 | 15.7 |
| 19374 | Cohort 6 | 2 | 36 | 4.82 |
| 19374 | Cohort 6 | 3 | 48 | 1.49 |
| 14832 | Cohort 6 | 1 | 0 | <LLOQ (0.500) |
| 14832 | Cohort 6 | 1 | 0.5 | 2570 |
| 14832 | Cohort 6 | 1 | 1 | 3360 |
| 14832 | Cohort 6 | 1 | 1.5 | 2710 |
| 14832 | Cohort 6 | 1 | 2 | 2390 |
| 14832 | Cohort 6 | 1 | 2.5 | 2080 |
| 14832 | Cohort 6 | 1 | 3 | 2010 |
| 14832 | Cohort 6 | 1 | 4 | 1550 |
| 14832 | Cohort 6 | 1 | 6 | 780 |
| 14832 | Cohort 6 | 1 | 9 | 395 |
| 14832 | Cohort 6 | 1 | 12 | 198 |
| 14832 | Cohort 6 | 1 | 18 | 101 |
| 14832 | Cohort 6 | 2 | 24 | 53.2 |
| 14832 | Cohort 6 | 2 | 36 | 14.5 |
| 14832 | Cohort 6 | 3 | 48 | 6.57 |
| 12125 | Cohort 6 | 1 | 0 | <LLOQ (0.500) |
| 12125 | Cohort 6 | 1 | 0.5 | <LLOQ (0.500) |
| 12125 | Cohort 6 | 1 | 1 | <LLOQ (0.500) |
| 12125 | Cohort 6 | 1 | 1.5 | <LLOQ (0.500) |
| 12125 | Cohort 6 | 1 | 2 | <LLOQ (0.500) |
| 12125 | Cohort 6 | 1 | 2.5 | <LLOQ (0.500) |
| 12125 | Cohort 6 | 1 | 3 | <LLOQ (0.500) |
| 12125 | Cohort 6 | 1 | 4 | <LLOQ (0.500) |
| 12125 | Cohort 6 | 1 | 6 | <LLOQ (0.500) |
| 12125 | Cohort 6 | 1 | 9 | <LLOQ (0.500) |
| 12125 | Cohort 6 | 1 | 12 | <LLOQ (0.500) |
| 12125 | Cohort 6 | 1 | 18 | <LLOQ (0.500) |
| 12125 | Cohort 6 | 2 | 24 | <LLOQ (0.500) |
| 12125 | Cohort 6 | 2 | 36 | <LLOQ (0.500) |
| 12125 | Cohort 6 | 3 | 48 | <LLOQ (0.500) |
| 16003 | Cohort 7 | 1 | 0 | <LLOQ (0.500) |
| 16003 | Cohort 7 | 1 | 0.5 | <LLOQ (0.500) |
| 16003 | Cohort 7 | 1 | 1 | <LLOQ (0.500) |
| 16003 | Cohort 7 | 1 | 1.5 | <LLOQ (0.500) |
| 16003 | Cohort 7 | 1 | 2 | <LLOQ (0.500) |
| 16003 | Cohort 7 | 1 | 2.5 | <LLOQ (0.500) |
| 16003 | Cohort 7 | 1 | 3 | <LLOQ (0.500) |
| 16003 | Cohort 7 | 1 | 4 | <LLOQ (0.500) |
| 16003 | Cohort 7 | 1 | 6 | <LLOQ (0.500) |
| 16003 | Cohort 7 | 1 | 9 | <LLOQ (0.500) |
| 16003 | Cohort 7 | 1 | 12 | <LLOQ (0.500) |
| 16003 | Cohort 7 | 1 | 18 | <LLOQ (0.500) |
| 16003 | Cohort 7 | 2 | 24 | <LLOQ (0.500) |
| 16003 | Cohort 7 | 2 | 36 | <LLOQ (0.500) |
| 16003 | Cohort 7 | 3 | 48 | <LLOQ (0.500) |
| 17839 | Cohort 7 | 1 | 0 | <LLOQ (0.500) |
| 17839 | Cohort 7 | 1 | 0.5 | 7310 |
| 17839 | Cohort 7 | 1 | 1 | 7980 |
| 17839 | Cohort 7 | 1 | 1.5 | 6910 |
| 17839 | Cohort 7 | 1 | 2 | 5860 |
| 17839 | Cohort 7 | 1 | 2.5 | 4890 |
| 17839 | Cohort 7 | 1 | 3 | 3990 |
| 17839 | Cohort 7 | 1 | 4 | 2760 |
| 17839 | Cohort 7 | 1 | 6 | 1580 |
| 17839 | Cohort 7 | 1 | 9 | 726 |
| 17839 | Cohort 7 | 1 | 12 | 335 |
| 17839 | Cohort 7 | 1 | 18 | 110 |
| 17839 | Cohort 7 | 2 | 24 | 58.7 |
| 17839 | Cohort 7 | 2 | 36 | 17.4 |
| 17839 | Cohort 7 | 3 | 48 | 7.94 |
| 18344 | Cohort 7 | 1 | 0 | <LLOQ (0.500) |
| 18344 | Cohort 7 | 1 | 0.5 | <LLOQ (0.500) |
| 18344 | Cohort 7 | 1 | 1 | <LLOQ (0.500) |
| 18344 | Cohort 7 | 1 | 1.5 | <LLOQ (0.500) |
| 18344 | Cohort 7 | 1 | 2 | <LLOQ (0.500) |
| 18344 | Cohort 7 | 1 | 2.5 | <LLOQ (0.500) |
| 18344 | Cohort 7 | 1 | 3 | <LLOQ (0.500) |
| 18344 | Cohort 7 | 1 | 4 | <LLOQ (0.500) |
| 18344 | Cohort 7 | 1 | 6 | <LLOQ (0.500) |
| 18344 | Cohort 7 | 1 | 9 | <LLOQ (0.500) |
| 18344 | Cohort 7 | 1 | 12 | <LLOQ (0.500) |
| 18344 | Cohort 7 | 1 | 18 | <LLOQ (0.500) |
| 18344 | Cohort 7 | 2 | 24 | <LLOQ (0.500) |
| 18344 | Cohort 7 | 2 | 36 | <LLOQ (0.500) |
| 18344 | Cohort 7 | 3 | 48 | <LLOQ (0.500) |
| 12147 | Cohort 7 | 1 | 0 | <LLOQ (0.500) |
| 12147 | Cohort 7 | 1 | 0.5 | 8420 |
| 12147 | Cohort 7 | 1 | 1 | 8390 |
| 12147 | Cohort 7 | 1 | 1.5 | 7250 |
| 12147 | Cohort 7 | 1 | 2 | 5560 |
| 12147 | Cohort 7 | 1 | 2.5 | 4270 |
| 12147 | Cohort 7 | 1 | 3 | 3650 |
| 12147 | Cohort 7 | 1 | 4 | 2230 |
| 12147 | Cohort 7 | 1 | 6 | 1120 |
| 12147 | Cohort 7 | 1 | 9 | 429 |
| 12147 | Cohort 7 | 1 | 12 | 184 |
| 12147 | Cohort 7 | 1 | 18 | 47.6 |
| 12147 | Cohort 7 | 2 | 24 | 27.8 |
| 12147 | Cohort 7 | 2 | 36 | 8.03 |
| 12147 | Cohort 7 | 3 | 48 | 3.58 |
| 17477 | Cohort 7 | 1 | 0 | <LLOQ (0.500) |
| 17477 | Cohort 7 | 1 | 0.5 | 5960 |
| 17477 | Cohort 7 | 1 | 1 | 11600 |
| 17477 | Cohort 7 | 1 | 1.5 | 7490 |
| 17477 | Cohort 7 | 1 | 2 | 6260 |
| 17477 | Cohort 7 | 1 | 2.5 | 6030 |
| 17477 | Cohort 7 | 1 | 3 | 5660 |
| 17477 | Cohort 7 | 1 | 4 | 3760 |
| 17477 | Cohort 7 | 1 | 6 | 1930 |
| 17477 | Cohort 7 | 1 | 9 | 642 |
| 17477 | Cohort 7 | 1 | 12 | 254 |
| 17477 | Cohort 7 | 1 | 18 | 69.6 |
| 17477 | Cohort 7 | 2 | 24 | 38.9 |
| 17477 | Cohort 7 | 2 | 36 | 10.9 |
| 17477 | Cohort 7 | 3 | 48 | 4.75 |
| 12292 | Cohort 7 | 1 | 0 | <LLOQ (0.500) |
| 12292 | Cohort 7 | 1 | 0.5 | 5510 |
| 12292 | Cohort 7 | 1 | 1 | 8700 |
| 12292 | Cohort 7 | 1 | 1.5 | 5760 |
| 12292 | Cohort 7 | 1 | 2 | 4660 |
| 12292 | Cohort 7 | 1 | 2.5 | 3950 |
| 12292 | Cohort 7 | 1 | 3 | 3610 |
| 12292 | Cohort 7 | 1 | 4 | 2630 |
| 12292 | Cohort 7 | 1 | 6 | 1770 |
| 12292 | Cohort 7 | 1 | 9 | 790 |
| 12292 | Cohort 7 | 1 | 12 | 352 |
| 12292 | Cohort 7 | 1 | 18 | 82.9 |
| 12292 | Cohort 7 | 2 | 24 | 46.4 |
| 12292 | Cohort 7 | 2 | 36 | 14.8 |
| 12292 | Cohort 7 | 3 | 48 | 6.57 |
| 14300 | Cohort 7 | 1 | 0 | <LLOQ (0.500) |
| 14300 | Cohort 7 | 1 | 0.5 | 13200 |
| 14300 | Cohort 7 | 1 | 1 | 11700 |
| 14300 | Cohort 7 | 1 | 1.5 | 7510 |
| 14300 | Cohort 7 | 1 | 2 | 6160 |
| 14300 | Cohort 7 | 1 | 2.5 | 4710 |
| 14300 | Cohort 7 | 1 | 3 | 4080 |
| 14300 | Cohort 7 | 1 | 4 | 3120 |
| 14300 | Cohort 7 | 1 | 6 | 1610 |
| 14300 | Cohort 7 | 1 | 9 | 588 |
| 14300 | Cohort 7 | 1 | 12 | 266 |
| 14300 | Cohort 7 | 1 | 18 | 80.3 |
| 14300 | Cohort 7 | 2 | 24 | 45.6 |
| 14300 | Cohort 7 | 2 | 36 | 14.8 |
| 14300 | Cohort 7 | 3 | 48 | 8.76 |
| 11920 | Cohort 7 | 1 | 0 | <LLOQ (0.500) |
| 11920 | Cohort 7 | 1 | 0.5 | 9650 |
| 11920 | Cohort 7 | 1 | 1 | 8870 |
| 11920 | Cohort 7 | 1 | 1.5 | 6190 |
| 11920 | Cohort 7 | 1 | 2 | 5490 |
| 11920 | Cohort 7 | 1 | 2.5 | 4800 |
| 11920 | Cohort 7 | 1 | 3 | 4540 |
| 11920 | Cohort 7 | 1 | 4 | 3370 |
| 11920 | Cohort 7 | 1 | 6 | 1730 |
| 11920 | Cohort 7 | 1 | 9 | 656 |
| 11920 | Cohort 7 | 1 | 12 | 301 |
| 11920 | Cohort 7 | 1 | 18 | 78.0 |
| 11920 | Cohort 7 | 2 | 24 | 40.8 |
| 11920 | Cohort 7 | 2 | 36 | 12.7 |
| 11920 | Cohort 7 | 3 | 48 | 6.60 |
| 15405 | Cohort 8 | 1 | 0 | <LLOQ (0.500) |
| 15405 | Cohort 8 | 1 | 0.5 | <LLOQ (0.500) |
| 15405 | Cohort 8 | 1 | 1 | <LLOQ (0.500) |
| 15405 | Cohort 8 | 1 | 1.5 | <LLOQ (0.500) |
| 15405 | Cohort 8 | 1 | 2 | <LLOQ (0.500) |
| 15405 | Cohort 8 | 1 | 2.5 | <LLOQ (0.500) |
| 15405 | Cohort 8 | 1 | 3 | <LLOQ (0.500) |
| 15405 | Cohort 8 | 1 | 4 | <LLOQ (0.500) |
| 15405 | Cohort 8 | 1 | 6 | <LLOQ (0.500) |
| 15405 | Cohort 8 | 1 | 9 | <LLOQ (0.500) |
| 15405 | Cohort 8 | 1 | 12 | <LLOQ (0.500) |
| 15405 | Cohort 8 | 1 | 18 | <LLOQ (0.500) |
| 15405 | Cohort 8 | 2 | 24 | <LLOQ (0.500) |
| 15405 | Cohort 8 | 2 | 36 | <LLOQ (0.500) |
| 15405 | Cohort 8 | 3 | 48 | <LLOQ (0.500) |
| 18449 | Cohort 8 | 1 | 0 | <LLOQ (0.500) |
| 18449 | Cohort 8 | 1 | 0.5 | 11200 |
| 18449 | Cohort 8 | 1 | 1 | 13400 |
| 18449 | Cohort 8 | 1 | 1.5 | 9360 |
| 18449 | Cohort 8 | 1 | 2 | 7490 |
| 18449 | Cohort 8 | 1 | 2.5 | 6200 |
| 18449 | Cohort 8 | 1 | 3 | 4860 |
| 18449 | Cohort 8 | 1 | 4 | 3470 |
| 18449 | Cohort 8 | 1 | 6 | 1670 |
| 18449 | Cohort 8 | 1 | 9 | 700 |
| 18449 | Cohort 8 | 1 | 12 | 295 |
| 18449 | Cohort 8 | 1 | 18 | 81.5 |
| 18449 | Cohort 8 | 2 | 24 | 40.1 |
| 18449 | Cohort 8 | 2 | 36 | 10.8 |
| 18449 | Cohort 8 | 3 | 48 | 4.95 |
| 17701 | Cohort 8 | 1 | 0 | <LLOQ (0.500) |
| 17701 | Cohort 8 | 1 | 0.5 | 11500 |
| 17701 | Cohort 8 | 1 | 1 | 12200 |
| 17701 | Cohort 8 | 1 | 1.5 | 10500 |
| 17701 | Cohort 8 | 1 | 2 | 8550 |
| 17701 | Cohort 8 | 1 | 2.5 | 7230 |
| 17701 | Cohort 8 | 1 | 3 | 6530 |
| 17701 | Cohort 8 | 1 | 4 | 3670 |
| 17701 | Cohort 8 | 1 | 6 | 2340 |
| 17701 | Cohort 8 | 1 | 9 | 1020 |
| 17701 | Cohort 8 | 1 | 12 | 459 |
| 17701 | Cohort 8 | 1 | 18 | 150 |
| 17701 | Cohort 8 | 2 | 24 | 79.3 |
| 17701 | Cohort 8 | 2 | 36 | 27.8 |
| 17701 | Cohort 8 | 3 | 48 | 14.3 |
| 16974 | Cohort 8 | 1 | 0 | <LLOQ (0.500) |
| 16974 | Cohort 8 | 1 | 0.5 | <LLOQ (0.500) |
| 16974 | Cohort 8 | 1 | 1 | <LLOQ (0.500) |
| 16974 | Cohort 8 | 1 | 1.5 | <LLOQ (0.500) |
| 16974 | Cohort 8 | 1 | 2 | <LLOQ (0.500) |
| 16974 | Cohort 8 | 1 | 2.5 | <LLOQ (0.500) |
| 16974 | Cohort 8 | 1 | 3 | <LLOQ (0.500) |
| 16974 | Cohort 8 | 1 | 4 | <LLOQ (0.500) |
| 16974 | Cohort 8 | 1 | 6 | <LLOQ (0.500) |
| 16974 | Cohort 8 | 1 | 9 | <LLOQ (0.500) |
| 16974 | Cohort 8 | 1 | 12 | <LLOQ (0.500) |
| 16974 | Cohort 8 | 1 | 18 | <LLOQ (0.500) |
| 16974 | Cohort 8 | 2 | 24 | <LLOQ (0.500) |
| 16974 | Cohort 8 | 2 | 36 | <LLOQ (0.500) |
| 16974 | Cohort 8 | 3 | 48 | <LLOQ (0.500) |
| 15959 | Cohort 8 | 1 | 0 | <LLOQ (0.500) |
| 15959 | Cohort 8 | 1 | 0.5 | 7720 |
| 15959 | Cohort 8 | 1 | 1 | 7950 |
| 15959 | Cohort 8 | 1 | 1.5 | 11700 |
| 15959 | Cohort 8 | 1 | 2 | 10100 |
| 15959 | Cohort 8 | 1 | 2.5 | 7620 |
| 15959 | Cohort 8 | 1 | 3 | 7090 |
| 15959 | Cohort 8 | 1 | 4 | 4190 |
| 15959 | Cohort 8 | 1 | 6 | 1840 |
| 15959 | Cohort 8 | 1 | 9 | 537 |
| 15959 | Cohort 8 | 1 | 12 | 226 |
| 15959 | Cohort 8 | 1 | 18 | 54.9 |
| 15959 | Cohort 8 | 2 | 24 | 31.4 |
| 15959 | Cohort 8 | 2 | 36 | 17.2 |
| 15959 | Cohort 8 | 3 | 48 | 9.11 |
| 12217 | Cohort 8 | 1 | 0 | <LLOQ (0.500) |
| 12217 | Cohort 8 | 1 | 0.5 | 12500 |
| 12217 | Cohort 8 | 1 | 1 | 9140 |
| 12217 | Cohort 8 | 1 | 1.5 | 8930 |
| 12217 | Cohort 8 | 1 | 2 | 6940 |
| 12217 | Cohort 8 | 1 | 2.5 | 5150 |
| 12217 | Cohort 8 | 1 | 3 | 4480 |
| 12217 | Cohort 8 | 1 | 4 | 3120 |
| 12217 | Cohort 8 | 1 | 6 | 1530 |
| 12217 | Cohort 8 | 1 | 9 | 509 |
| 12217 | Cohort 8 | 1 | 12 | 217 |
| 12217 | Cohort 8 | 1 | 18 | 64.0 |
| 12217 | Cohort 8 | 2 | 24 | 30.4 |
| 12217 | Cohort 8 | 2 | 36 | 9.14 |
| 12217 | Cohort 8 | 3 | 48 | 3.54 |
| 13625 | Cohort 8 | 1 | 0 | <LLOQ (0.500) |
| 13625 | Cohort 8 | 1 | 0.5 | 17500 |
| 13625 | Cohort 8 | 1 | 1 | 15500 |
| 13625 | Cohort 8 | 1 | 1.5 | 10500 |
| 13625 | Cohort 8 | 1 | 2 | 9260 |
| 13625 | Cohort 8 | 1 | 2.5 | 6750 |
| 13625 | Cohort 8 | 1 | 3 | 6240 |
| 13625 | Cohort 8 | 1 | 4 | 4650 |
| 13625 | Cohort 8 | 1 | 6 | 2180 |
| 13625 | Cohort 8 | 1 | 9 | 938 |
| 13625 | Cohort 8 | 1 | 12 | 373 |
| 13625 | Cohort 8 | 1 | 18 | 119 |
| 13625 | Cohort 8 | 2 | 24 | 62.3 |
| 13625 | Cohort 8 | 2 | 36 | 19.1 |
| 13625 | Cohort 8 | 3 | 48 | 9.50 |
| 10101 | Cohort 8 | 1 | 0 | <LLOQ (0.500) |
| 10101 | Cohort 8 | 1 | 0.5 | 6560 |
| 10101 | Cohort 8 | 1 | 1 | 11500 |
| 10101 | Cohort 8 | 1 | 1.5 | 9030 |
| 10101 | Cohort 8 | 1 | 2 | 6970 |
| 10101 | Cohort 8 | 1 | 2.5 | 7260 |
| 10101 | Cohort 8 | 1 | 3 | 6800 |
| 10101 | Cohort 8 | 1 | 4 | 4750 |
| 10101 | Cohort 8 | 1 | 6 | 2080 |
| 10101 | Cohort 8 | 1 | 9 | 707 |
| 10101 | Cohort 8 | 1 | 12 | 272 |
| 10101 | Cohort 8 | 1 | 18 | 74.2 |
| 10101 | Cohort 8 | 2 | 24 | 38.8 |
| 10101 | Cohort 8 | 2 | 36 | 9.17 |
| 10101 | Cohort 8 | 3 | 48 | 3.32 |

<LLOQ: Less than the Lower Limit of Quantitation, listed in parentheses.

Concentrations of UV-4 in Human Urine Samples

(Callahan, UV4 FIH, Supplemental Information)

| SUBJECT  ALIAS ID | SUBJECT  GROUP | NOMINAL DAY | NOMINAL  HOUR | CONCENTRATION UV4 (ng/mL) |
| --- | --- | --- | --- | --- |
| 11290 | Cohort 1 | 1 | 0 | <LLOQ (0.500) |
| 11290 | Cohort 1 | 1 | 6 | 427 |
| 11290 | Cohort 1 | 1 | 12 | 247 |
| 11290 | Cohort 1 | 2 | 24 | 157 |
| 11290 | Cohort 1 | 2 | 48 | 40.6 |
| 17603 | Cohort 1 | 1 | 0 | <LLOQ (0.500) |
| 17603 | Cohort 1 | 1 | 6 | <LLOQ (0.500) |
| 17603 | Cohort 1 | 1 | 12 | <LLOQ (0.500) |
| 17603 | Cohort 1 | 2 | 24 | <LLOQ (0.500) |
| 17603 | Cohort 1 | 2 | 48 | <LLOQ (0.500) |
| 12126 | Cohort 1 | 1 | 0 | <LLOQ (0.500) |
| 12126 | Cohort 1 | 1 | 6 | 650 |
| 12126 | Cohort 1 | 1 | 12 | 414 |
| 12126 | Cohort 1 | 2 | 24 | 99.1 |
| 12126 | Cohort 1 | 2 | 48 | 74.6 |
| 10888 | Cohort 1 | 1 | 0 | <LLOQ (0.500) |
| 10888 | Cohort 1 | 1 | 6 | 868 |
| 10888 | Cohort 1 | 1 | 12 | 455 |
| 10888 | Cohort 1 | 2 | 24 | 451 |
| 10888 | Cohort 1 | 2 | 48 | 166 |
| 12604 | Cohort 1 | 1 | 0 | <LLOQ (0.500) |
| 12604 | Cohort 1 | 1 | 6 | 531 |
| 12604 | Cohort 1 | 1 | 12 | 396 |
| 12604 | Cohort 1 | 2 | 24 | 272 |
| 12604 | Cohort 1 | 2 | 48 | 97.2 |
| 19993 | Cohort 1 | 1 | 0 | <LLOQ (0.500) |
| 19993 | Cohort 1 | 1 | 6 | 376 |
| 19993 | Cohort 1 | 1 | 12 | 552 |
| 19993 | Cohort 1 | 2 | 24 | 167 |
| 19993 | Cohort 1 | 2 | 48 | 110 |
| 15202 | Cohort 1 | 1 | 0 | <LLOQ (0.500) |
| 15202 | Cohort 1 | 1 | 6 | 2510 |
| 15202 | Cohort 1 | 1 | 12 | 892 |
| 15202 | Cohort 1 | 2 | 24 | 568 |
| 15202 | Cohort 1 | 2 | 48 | 228 |
| 16861 | Cohort 1 | 1 | 0 | <LLOQ (0.500) |
| 16861 | Cohort 1 | 1 | 6 | <LLOQ (0.500) |
| 16861 | Cohort 1 | 1 | 12 | <LLOQ (0.500) |
| 16861 | Cohort 1 | 2 | 24 | <LLOQ (0.500) |
| 16861 | Cohort 1 | 2 | 48 | <LLOQ (0.500) |
| 14981 | Cohort 2 | 1 | 0 | <LLOQ (0.500) |
| 14981 | Cohort 2 | 1 | 6 | <LLOQ (0.500) |
| 14981 | Cohort 2 | 1 | 12 | <LLOQ (0.500) |
| 14981 | Cohort 2 | 2 | 24 | <LLOQ (0.500) |
| 14981 | Cohort 2 | 2 | 48 | <LLOQ (0.500) |
| 15473 | Cohort 2 | 1 | 0 | <LLOQ (0.500) |
| 15473 | Cohort 2 | 1 | 6 | 3060 |
| 15473 | Cohort 2 | 1 | 12 | 1970 |
| 15473 | Cohort 2 | 2 | 24 | 627 |
| 15473 | Cohort 2 | 2 | 48 | 270 |
| 17509 | Cohort 2 | 1 | 0 | <LLOQ (0.500) |
| 17509 | Cohort 2 | 1 | 6 | <LLOQ (0.500) |
| 17509 | Cohort 2 | 1 | 12 | <LLOQ (0.500) |
| 17509 | Cohort 2 | 2 | 24 | <LLOQ (0.500) |
| 17509 | Cohort 2 | 2 | 48 | <LLOQ (0.500) |
| 18798 | Cohort 2 | 1 | 0 | <LLOQ (0.500) |
| 18798 | Cohort 2 | 1 | 6 | 3440 |
| 18798 | Cohort 2 | 1 | 12 | 2760 |
| 18798 | Cohort 2 | 2 | 24 | 606 |
| 18798 | Cohort 2 | 2 | 48 | 226 |
| 16510 | Cohort 2 | 1 | 0 | <LLOQ (0.500) |
| 16510 | Cohort 2 | 1 | 6 | 1560 |
| 16510 | Cohort 2 | 1 | 12 | 473 |
| 16510 | Cohort 2 | 2 | 24 | 331 |
| 16510 | Cohort 2 | 2 | 48 | 125 |
| 12612 | Cohort 2 | 1 | 0 | <LLOQ (0.500) |
| 12612 | Cohort 2 | 1 | 6 | 2360 |
| 12612 | Cohort 2 | 1 | 12 | 1110 |
| 12612 | Cohort 2 | 2 | 24 | 1240 |
| 12612 | Cohort 2 | 2 | 48 | 215 |
| 15239 | Cohort 2 | 1 | 0 | <LLOQ (0.500) |
| 15239 | Cohort 2 | 1 | 6 | 3250 |
| 15239 | Cohort 2 | 1 | 12 | 4010 |
| 15239 | Cohort 2 | 2 | 24 | 1170 |
| 15239 | Cohort 2 | 2 | 48 | 387 |
| 15090 | Cohort 2 | 1 | 0 | <LLOQ (0.500) |
| 15090 | Cohort 2 | 1 | 6 | 3650 |
| 15090 | Cohort 2 | 1 | 12 | 1650 |
| 15090 | Cohort 2 | 2 | 24 | 1170 |
| 15090 | Cohort 2 | 2 | 48 | 304 |
| 19949 | Cohort 3 | 1 | 0 | <LLOQ (0.500) |
| 19949 | Cohort 3 | 1 | 6 | 16500 |
| 19949 | Cohort 3 | 1 | 12 | 8670 |
| 19949 | Cohort 3 | 2 | 24 | 2700 |
| 19949 | Cohort 3 | 2 | 48 | 685 |
| 17096 | Cohort 3 | 1 | 0 | <LLOQ (0.500) |
| 17096 | Cohort 3 | 1 | 6 | <LLOQ (0.500) |
| 17096 | Cohort 3 | 1 | 12 | <LLOQ (0.500) |
| 17096 | Cohort 3 | 2 | 24 | <LLOQ (0.500) |
| 17096 | Cohort 3 | 2 | 48 | <LLOQ (0.500) |
| 18776 | Cohort 3 | 1 | 0 | <LLOQ (0.500) |
| 18776 | Cohort 3 | 1 | 6 | 16100 |
| 18776 | Cohort 3 | 1 | 12 | 7570 |
| 18776 | Cohort 3 | 2 | 24 | 1020 |
| 18776 | Cohort 3 | 2 | 48 | 237 |
| 14041 | Cohort 3 | 1 | 0 | <LLOQ (0.500) |
| 14041 | Cohort 3 | 1 | 6 | 19700 |
| 14041 | Cohort 3 | 1 | 12 | 8820 |
| 14041 | Cohort 3 | 2 | 24 | 3300 |
| 14041 | Cohort 3 | 2 | 48 | 721 |
| 17172 | Cohort 3 | 1 | 0 | <LLOQ (0.500) |
| 17172 | Cohort 3 | 1 | 6 | <LLOQ (0.500) |
| 17172 | Cohort 3 | 1 | 12 | <LLOQ (0.500) |
| 17172 | Cohort 3 | 2 | 24 | <LLOQ (0.500) |
| 17172 | Cohort 3 | 2 | 48 | <LLOQ (0.500) |
| 14265 | Cohort 3 | 1 | 0 | <LLOQ (0.500) |
| 14265 | Cohort 3 | 1 | 6 | 6240 |
| 14265 | Cohort 3 | 1 | 12 | 2090 |
| 14265 | Cohort 3 | 2 | 24 | 754 |
| 14265 | Cohort 3 | 2 | 48 | 248 |
| 11117 | Cohort 3 | 1 | 0 | <LLOQ (0.500) |
| 11117 | Cohort 3 | 1 | 6 | 8650 |
| 11117 | Cohort 3 | 1 | 12 | 1200 |
| 11117 | Cohort 3 | 2 | 24 | 614 |
| 11117 | Cohort 3 | 2 | 48 | 147 |
| 16824 | Cohort 3 | 1 | 0 | <LLOQ (0.500) |
| 16824 | Cohort 3 | 1 | 6 | 11700 |
| 16824 | Cohort 3 | 1 | 12 | 11200 |
| 16824 | Cohort 3 | 2 | 24 | 3690 |
| 16824 | Cohort 3 | 2 | 48 | 900 |
| 14776 | Cohort 4 | 1 | 0 | <LLOQ (0.500) |
| 14776 | Cohort 4 | 1 | 6 | <LLOQ (0.500) |
| 14776 | Cohort 4 | 1 | 12 | <LLOQ (0.500) |
| 14776 | Cohort 4 | 2 | 24 | <LLOQ (0.500) |
| 14776 | Cohort 4 | 2 | 48 | <LLOQ (0.500) |
| 13523 | Cohort 4 | 1 | 0 | <LLOQ (0.500) |
| 13523 | Cohort 4 | 1 | 6 | 46400 |
| 13523 | Cohort 4 | 1 | 12 | 3970 |
| 13523 | Cohort 4 | 2 | 24 | 1230 |
| 13523 | Cohort 4 | 2 | 48 | 367 |
| 12146 | Cohort 4 | 1 | 0 | <LLOQ (0.500) |
| 12146 | Cohort 4 | 1 | 6 | 48400 |
| 12146 | Cohort 4 | 1 | 12 | 24500 |
| 12146 | Cohort 4 | 2 | 24 | 7560 |
| 12146 | Cohort 4 | 2 | 48 | 2090 |
| 11798 | Cohort 4 | 1 | 0 | <LLOQ (0.500) |
| 11798 | Cohort 4 | 1 | 6 | <LLOQ (0.500) |
| 11798 | Cohort 4 | 1 | 12 | <LLOQ (0.500) |
| 11798 | Cohort 4 | 2 | 24 | <LLOQ (0.500) |
| 11798 | Cohort 4 | 2 | 48 | <LLOQ (0.500) |
| 10291 | Cohort 4 | 1 | 0 | <LLOQ (0.500) |
| 10291 | Cohort 4 | 1 | 6 | 30900 |
| 10291 | Cohort 4 | 1 | 12 | 26000 |
| 10291 | Cohort 4 | 2 | 24 | 8120 |
| 10291 | Cohort 4 | 2 | 48 | 1260 |
| 19130 | Cohort 4 | 1 | 0 | 1.11 |
| 19130 | Cohort 4 | 1 | 6 | 140000 |
| 19130 | Cohort 4 | 1 | 12 | 15000 |
| 19130 | Cohort 4 | 2 | 24 | 8370 |
| 19130 | Cohort 4 | 2 | 48 | 1810 |
| 14697 | Cohort 4 | 1 | 0 | <LLOQ (0.500) |
| 14697 | Cohort 4 | 1 | 6 | 54900 |
| 14697 | Cohort 4 | 1 | 12 | 33100 |
| 14697 | Cohort 4 | 2 | 24 | 4550 |
| 14697 | Cohort 4 | 2 | 48 | 473 |
| 10007 | Cohort 4 | 1 | 0 | <LLOQ (0.500) |
| 10007 | Cohort 4 | 1 | 6 | 36000 |
| 10007 | Cohort 4 | 1 | 12 | 11200 |
| 10007 | Cohort 4 | 2 | 24 | 3920 |
| 10007 | Cohort 4 | 2 | 48 | 648 |
| 11471 | Cohort 5 | 1 | 0 | <LLOQ (0.500) |
| 11471 | Cohort 5 | 1 | 6 | <LLOQ (0.500) |
| 11471 | Cohort 5 | 1 | 12 | <LLOQ (0.500) |
| 11471 | Cohort 5 | 2 | 24 | <LLOQ (0.500) |
| 11471 | Cohort 5 | 2 | 48 | <LLOQ (0.500) |
| 10742 | Cohort 5 | 1 | 0 | <LLOQ (0.500) |
| 10742 | Cohort 5 | 1 | 6 | 122000 |
| 10742 | Cohort 5 | 1 | 12 | 17100 |
| 10742 | Cohort 5 | 2 | 24 | 5200 |
| 10742 | Cohort 5 | 2 | 48 | 706 |
| 12717 | Cohort 5 | 1 | 0 | <LLOQ (0.500) |
| 12717 | Cohort 5 | 1 | 6 | 104000 |
| 12717 | Cohort 5 | 1 | 12 | 26900 |
| 12717 | Cohort 5 | 2 | 24 | 6760 |
| 12717 | Cohort 5 | 2 | 48 | 961 |
| 19239 | Cohort 5 | 1 | 0 | <LLOQ (0.500) |
| 19239 | Cohort 5 | 1 | 6 | 75800 |
| 19239 | Cohort 5 | 1 | 12 | 16200 |
| 19239 | Cohort 5 | 2 | 24 | 4560 |
| 19239 | Cohort 5 | 2 | 48 | 775 |
| 14482 | Cohort 5 | 1 | 0 | <LLOQ (0.500) |
| 14482 | Cohort 5 | 1 | 6 | 62800 |
| 14482 | Cohort 5 | 1 | 12 | 22000 |
| 14482 | Cohort 5 | 2 | 24 | 7550 |
| 14482 | Cohort 5 | 2 | 48 | 2920 |
| 19884 | Cohort 5 | 1 | 0 | <LLOQ (0.500) |
| 19884 | Cohort 5 | 1 | 6 | <LLOQ (0.500) |
| 19884 | Cohort 5 | 1 | 12 | <LLOQ (0.500) |
| 19884 | Cohort 5 | 2 | 24 | <LLOQ (0.500) |
| 19884 | Cohort 5 | 2 | 48 | <LLOQ (0.500) |
| 10558 | Cohort 5 | 1 | 0 | <LLOQ (0.500) |
| 10558 | Cohort 5 | 1 | 6 | 65800 |
| 10558 | Cohort 5 | 1 | 12 | 21200 |
| 10558 | Cohort 5 | 2 | 24 | 11900 |
| 10558 | Cohort 5 | 2 | 48 | 2030 |
| 12639 | Cohort 5 | 1 | 0 | <LLOQ (0.500) |
| 12639 | Cohort 5 | 1 | 6 | 68800 |
| 12639 | Cohort 5 | 1 | 12 | 18200 |
| 12639 | Cohort 5 | 2 | 24 | 7200 |
| 12639 | Cohort 5 | 2 | 48 | 1860 |
| 13778 | Cohort 6 | 1 | 0 | <LLOQ (0.500) |
| 13778 | Cohort 6 | 1 | 6 | <LLOQ (0.500) |
| 13778 | Cohort 6 | 1 | 12 | <LLOQ (0.500) |
| 13778 | Cohort 6 | 2 | 24 | <LLOQ (0.500) |
| 13778 | Cohort 6 | 2 | 48 | <LLOQ (0.500) |
| 15328 | Cohort 6 | 1 | 0 | <LLOQ (0.500) |
| 15328 | Cohort 6 | 1 | 6 | 357000 |
| 15328 | Cohort 6 | 1 | 12 | 35000 |
| 15328 | Cohort 6 | 2 | 24 | 6830 |
| 15328 | Cohort 6 | 2 | 48 | 1470 |
| 19876 | Cohort 6 | 1 | 0 | <LLOQ (0.500) |
| 19876 | Cohort 6 | 1 | 6 | 97600 |
| 19876 | Cohort 6 | 1 | 12 | 12100 |
| 19876 | Cohort 6 | 2 | 24 | 2550 |
| 19876 | Cohort 6 | 2 | 48 | 442 |
| 12062 | Cohort 6 | 1 | 0 | <LLOQ (0.500) |
| 12062 | Cohort 6 | 1 | 6 | 326000 |
| 12062 | Cohort 6 | 1 | 12 | 104000 |
| 12062 | Cohort 6 | 2 | 24 | 4060 |
| 12062 | Cohort 6 | 2 | 48 | 1480 |
| 18453 | Cohort 6 | 1 | 0 | <LLOQ (0.500) |
| 18453 | Cohort 6 | 1 | 6 | 75900 |
| 18453 | Cohort 6 | 1 | 12 | 38000 |
| 18453 | Cohort 6 | 2 | 24 | 7760 |
| 18453 | Cohort 6 | 2 | 48 | 1390 |
| 19374 | Cohort 6 | 1 | 0 | <LLOQ (0.500) |
| 19374 | Cohort 6 | 1 | 6 | 182000 |
| 19374 | Cohort 6 | 1 | 12 | 15200 |
| 19374 | Cohort 6 | 2 | 24 | 6220 |
| 19374 | Cohort 6 | 2 | 48 | 830 |
| 14832 | Cohort 6 | 1 | 0 | <LLOQ (0.500) |
| 14832 | Cohort 6 | 1 | 6 | 126000 |
| 14832 | Cohort 6 | 1 | 12 | 19200 |
| 14832 | Cohort 6 | 2 | 24 | 12700 |
| 14832 | Cohort 6 | 2 | 48 | 1210 |
| 12125 | Cohort 6 | 1 | 0 | <LLOQ (0.500) |
| 12125 | Cohort 6 | 1 | 6 | <LLOQ (0.500) |
| 12125 | Cohort 6 | 1 | 12 | <LLOQ (0.500) |
| 12125 | Cohort 6 | 2 | 24 | <LLOQ (0.500) |
| 12125 | Cohort 6 | 2 | 48 | <LLOQ (0.500) |
| 16003 | Cohort 7 | 1 | 0 | <LLOQ (0.500) |
| 16003 | Cohort 7 | 1 | 6 | <LLOQ (0.500) |
| 16003 | Cohort 7 | 1 | 12 | <LLOQ (0.500) |
| 16003 | Cohort 7 | 2 | 24 | <LLOQ (0.500) |
| 16003 | Cohort 7 | 2 | 48 | <LLOQ (0.500) |
| 17839 | Cohort 7 | 1 | 0 | <LLOQ (0.500) |
| 17839 | Cohort 7 | 1 | 6 | 599000 |
| 17839 | Cohort 7 | 1 | 12 | 54800 |
| 17839 | Cohort 7 | 2 | 24 | 9730 |
| 17839 | Cohort 7 | 2 | 48 | 3490 |
| 18344 | Cohort 7 | 1 | 0 | <LLOQ (0.500) |
| 18344 | Cohort 7 | 1 | 6 | <LLOQ (0.500) |
| 18344 | Cohort 7 | 1 | 12 | <LLOQ (0.500) |
| 18344 | Cohort 7 | 2 | 24 | <LLOQ (0.500) |
| 18344 | Cohort 7 | 2 | 48 | <LLOQ (0.500) |
| 12147 | Cohort 7 | 1 | 0 | <LLOQ (0.500) |
| 12147 | Cohort 7 | 1 | 6 | 416000 |
| 12147 | Cohort 7 | 1 | 12 | 44700 |
| 12147 | Cohort 7 | 2 | 24 | 10700 |
| 12147 | Cohort 7 | 2 | 48 | 2450 |
| 17477 | Cohort 7 | 1 | 0 | <LLOQ (0.500) |
| 17477 | Cohort 7 | 1 | 6 | 268000 |
| 17477 | Cohort 7 | 1 | 12 | 120000 |
| 17477 | Cohort 7 | 2 | 24 | 23800 |
| 17477 | Cohort 7 | 2 | 48 | 3180 |
| 12292 | Cohort 7 | 1 | 0 | <LLOQ (0.500) |
| 12292 | Cohort 7 | 1 | 6 | 353000 |
| 12292 | Cohort 7 | 1 | 12 | 116000 |
| 12292 | Cohort 7 | 2 | 24 | 22700 |
| 12292 | Cohort 7 | 2 | 48 | 2230 |
| 14300 | Cohort 7 | 1 | 0 | <LLOQ (0.500) |
| 14300 | Cohort 7 | 1 | 6 | 330000 |
| 14300 | Cohort 7 | 1 | 12 | 118000 |
| 14300 | Cohort 7 | 2 | 24 | 12700 |
| 14300 | Cohort 7 | 2 | 48 | 2730 |
| 11920 | Cohort 7 | 1 | 0 | <LLOQ (0.500) |
| 11920 | Cohort 7 | 1 | 6 | 435000 |
| 11920 | Cohort 7 | 1 | 12 | 167000 |
| 11920 | Cohort 7 | 2 | 24 | 26100 |
| 11920 | Cohort 7 | 2 | 48 | 6020 |
| 15405 | Cohort 8 | 1 | 0 | <LLOQ (0.500) |
| 15405 | Cohort 8 | 1 | 6 | <LLOQ (0.500) |
| 15405 | Cohort 8 | 1 | 12 | <LLOQ (0.500) |
| 15405 | Cohort 8 | 2 | 24 | <LLOQ (0.500) |
| 15405 | Cohort 8 | 2 | 48 | <LLOQ (0.500) |
| 18449 | Cohort 8 | 1 | 0 | <LLOQ (0.500) |
| 18449 | Cohort 8 | 1 | 6 | 643000 |
| 18449 | Cohort 8 | 1 | 12 | 69800 |
| 18449 | Cohort 8 | 2 | 24 | 10500 |
| 18449 | Cohort 8 | 2 | 48 | 1690 |
| 17701 | Cohort 8 | 1 | 0 | <LLOQ (0.500) |
| 17701 | Cohort 8 | 1 | 6 | 961000 |
| 17701 | Cohort 8 | 1 | 12 | 143000 |
| 17701 | Cohort 8 | 2 | 24 | 39600 |
| 17701 | Cohort 8 | 2 | 48 | 10400 |
| 16974 | Cohort 8 | 1 | 0 | <LLOQ (0.500) |
| 16974 | Cohort 8 | 1 | 6 | <LLOQ (0.500) |
| 16974 | Cohort 8 | 1 | 12 | <LLOQ (0.500) |
| 16974 | Cohort 8 | 2 | 24 | <LLOQ (0.500) |
| 16974 | Cohort 8 | 2 | 48 | <LLOQ (0.500) |
| 15959 | Cohort 8 | 1 | 0 | <LLOQ (0.500) |
| 15959 | Cohort 8 | 1 | 6 | 391000 |
| 15959 | Cohort 8 | 1 | 12 | 94400 |
| 15959 | Cohort 8 | 2 | 24 | 35500 |
| 15959 | Cohort 8 | 2 | 48 | 4270 |
| 12217 | Cohort 8 | 1 | 0 | <LLOQ (0.500) |
| 12217 | Cohort 8 | 1 | 6 | 289000 |
| 12217 | Cohort 8 | 1 | 12 | 37800 |
| 12217 | Cohort 8 | 2 | 24 | 7550 |
| 12217 | Cohort 8 | 2 | 48 | 1390 |
| 13625 | Cohort 8 | 1 | 0 | <LLOQ (0.500) |
| 13625 | Cohort 8 | 1 | 6 | 432000 |
| 13625 | Cohort 8 | 1 | 12 | 311000 |
| 13625 | Cohort 8 | 2 | 24 | 38800 |
| 13625 | Cohort 8 | 2 | 48 | 11000 |
| 10101 | Cohort 8 | 1 | 0 | <LLOQ (0.500) |
| 10101 | Cohort 8 | 1 | 6 | 396000 |
| 10101 | Cohort 8 | 1 | 12 | 113000 |
| 10101 | Cohort 8 | 2 | 24 | 21900 |
| 10101 | Cohort 8 | 2 | 48 | 3330 |

<LLOQ: Less than the Lower Limit of Quantitation, listed in parentheses.
