# Supplementary material for: Randomized single oral dose phase 1 study of safety, tolerability, and pharmacokinetics of Iminosugar UV-4 Hydrochloride (UV-4B) in healthy subjects
Source: PLoS Negl Trop Dis. 2022 Aug 8;16(8):e0010636. doi: 10.1371/journal.pntd.0010636 (PMC9387934; doi:10.1371/journal.pntd.0010636)
Supplement: S1 File — (PDF) [file pntd.0010636.s004.pdf]

RANDOMIZED, DOUBLE-BLIND, PLACEBO-CONTROLLED, PARALLEL GROUP, SINGLE-ASCENDING DOSE STUDY TO DETERMINE THE SAFETY, TOLERABILITY AND PHARMACOKINETICS OF UV-4B SOLUTION ADMINISTERED ORALLY IN HEALTHY SUBJECTS

DMID Protocol Number: DMID 13-0001

DMID Funding Mechanism: HHSN272201100030C

Contract Research Organization: Quintiles, Inc.

Other Identifying Numbers: UV-DEN-0001

Quintiles Study Number: 4991

IND Sponsor:
Unither Virology, LLC
1040 Spring Street
Silver Spring, MD 20910

Principal Investigator:
Thomas Murtaugh, MD, CPI
Quintiles Phase One Services, LLC

Unither Virology Medical Monitor:
Michael Callahan, MD, DTM&H, MSPH

DMID Medical Monitor:
Mohamed Elsafy, MD, MSc

Version Number: 7.0

25 June 2015

CONFIDENTIAL
This material is the property of the Unither Virology. Do not disclose or use except as authorized in writing by the study sponsor.

STATEMENT OF COMPLIANCE

The study will be carried out in accordance with ethical principles that have their origin in The Declaration of Helsinki and that are consistent with Good Clinical Practice (GCP) as required by the following:

- United States (US) Code of Federal Regulations (CFR) applicable to clinical studies (45 CFR Part 46, 21 CFR Part 50, 21 CFR Part 56, 21 CFR Part 11, and 21 CFR Part 312)
- International Conference on Harmonization (ICH) E6; 62 Federal Register 25691 (1997)
- National Institutes of Health (NIH) Clinical Terms of Award

All key personnel (all individuals responsible for the design and conduct of this study) have completed Human Subject's Protection Training.

CONFIDENTIAL
This material is the property of the Unither Virology. Do not disclose or use except as authorized in writing by the study sponsor.

UNITHER VIROLOGY SIGNATURE PAGE

The signature below constitutes the review and approval of this protocol.

Medical Monitor:

Signed: ______________________________

Name: Michael Callahan, MD, DTM&H, MSPH

Title: President and Chief Medical Officer

CONFIDENTIAL
This material is the property of the Unither Virology. Do not disclose or use except as authorized in writing by the study sponsor.

INVESTIGATOR SIGNATURE PAGE

The signature below constitutes the approval of this protocol and the attachments, and provides the necessary assurances that this study will be conducted according to all stipulations of the protocol, including all statements regarding confidentiality, and according to local legal and regulatory requirements and applicable US federal regulations and ICH guidelines.

Site Investigator:

Signed: [signature] Date: 25/Jun 2015

Name: Thomas Murtaugh, MD, CPI
Title: Principal Investigator

CONFIDENTIAL
This material is the property of the Unither Virology. Do not disclose or use except as authorized in writing by the study sponsor.

TABLE OF CONTENTS

Page
Statement of Compliance ii
Unither Virology Signature Page iii
Table of Contents v
List of Abbreviations x
Protocol Summary xiii
1 Key Roles 17
2 Background Information and Scientific Rationale 20
2.1 Background Information 20
2.1.1 Pharmacology 20
2.1.2 Nonclinical Pharmacokinetics 21
2.1.3 Safety Pharmacology and Toxicology 22
2.2 Rationale for Dose Selection 24
2.3 Potential Risks and Benefits 28
2.3.1 Potential Risks 28
2.3.2 Known Potential Benefits 32
3 Objectives 33
3.1 Study Objectives 33
3.1.1 Primary Objective 33
3.1.2 Secondary Objective 33
3.2 Study Outcome Measures 33
3.2.1 Primary Outcome Measures 33
3.2.2 Secondary Outcome Measures 33
4 Study Design 34
5 Study Enrollment and Withdrawal 35
5.1 Subject Inclusion Criteria 35
5.2 Subject Exclusion Criteria 36
5.3 Treatment Assignment Procedures 39
5.3.1 Randomization Procedures 39
5.3.2 Masking Procedures 39
5.3.3 Reasons for Withdrawal 40
5.3.4 Blinding Procedures 40
5.3.5 Emergency Unblinding 41
5.3.6 Termination of Study 42
6 Study Investigational Product 43
6.1 Study Product Description 43
6.1.1 Acquisition 43
6.1.2 Formulation, Packaging, and Labeling 43
6.1.3 Product Storage and Stability 43

CONFIDENTIAL
This material is the property of the Unither Virology. Do not disclose or use except as authorized in writing by the study sponsor.

6.2 Dosage, Preparation, and Administration of Study Investigational Product 44
6.3 Accountability Procedures for the Study Investigational Product 44
6.4 Assessment of Subject Compliance with Study Investigational Product 45
6.5 Concomitant Medications/Treatments 45
7 Study Schedule 46
7.1 Day -28 to Day -2 (Screening) 46
7.2 Enrollment/Baseline 46
7.2.1 Day -1 (Admission to Clinic; 24 hours prior to dosing) 46
7.3 Treatment Period 47
7.3.1 Day 1 (Dosing) 48
7.3.2 Day 2 In-Clinic Follow-up 49
7.3.3 Day 3 Discharge from Clinic 50
7.3.4 Day 4 (Outpatient Visit) 51
7.4 Day 9 ±1 Follow-up and Final Study Visit 51
7.5 Early Termination Visit 52
7.6 Unscheduled Visit 52
8 Study Procedures/Evaluations 53
8.1 Clinical Evaluations 53
8.2 Laboratory Evaluations 53
8.2.1 Clinical Laboratory Evaluations 53
8.2.2 Special Assays or Procedures 56
8.2.3 Specimen Preparation, Handling, and Shipping 57
8.3 Diet, Exercise, and Study Restrictions 57
9 Assessment of Safety 59
9.1 Specification of Safety Parameters 59
9.1.1 Adverse Events 59
9.1.2 Vital Signs 59
9.1.3 Electrocardiograms 59
9.1.4 Telemetry 61
9.1.5 Physical Examination 61
9.1.6 Clinical Laboratory Testing 62
9.1.7 Evaluation of Hypersensitivity Reactions 62
9.2 Methods and Timing for Assessing, Recording, and Analyzing Safety Parameters 62
9.2.1 Adverse Events 62
9.2.2 Serious Adverse Events 64
9.2.3 Procedures to be followed in the Event of Abnormal Laboratory Test Values or Abnormal Clinical Findings 65
9.3 Reporting Procedures 65
9.3.1 Serious Adverse Events 66
9.3.2 Reporting of Pregnancy 67

CONFIDENTIAL
This material is the property of the Unither Virology. Do not disclose or use except as authorized in writing by the study sponsor.

9.4 Type and Duration of Follow-up of Subjects after Adverse Events 67
9.5 Dose Escalation and Halting Rules 68
9.6 Safety Oversight (Safety Review Committee, Independent Safety Monitor plus Safety Monitoring Committee) 69
9.6.1 Independent Safety Monitor (ISM) 69
9.6.2 Safety Review Committee (SRC) 70
9.6.3 Safety Monitoring Committee (SMC) 70
9.6.4 Food and Drug Administration 71
10 Clinical Monitoring 72
10.1 Site Monitoring Plan 72
11 Statistical Considerations 73
11.1 Study Hypotheses 73
11.2 Sample Size Considerations 73
11.3 Planned Interim Analyses 73
11.3.1 Safety Review 73
11.3.2 Efficacy Review 73
11.3.3 Pharmacokinetic Review 73
11.4 Final Analysis Plan 73
11.4.1 Populations for Analysis 73
11.4.2 Randomization and Stratification 74
11.4.3 Procedures for Handling Missing, Unused, and Spurious Data 74
11.4.4 Statistical Plan 74
11.4.5 Safety 74
11.4.6 Pharmacokinetics 76
11.4.7 Deviation from Original Analysis Plan 77
12 Source Documents and Access to Source Data/Documents 78
13 Quality Control and Quality Assurance 79
14 Ethics/Protection of Human Subjects 80
14.1 Ethical Standard 80
14.2 Institutional Review Board 80
14.3 Informed Consent Process 80
14.4 Exclusion of Women, Minorities, and Children (Special Populations) 81
14.5 Subject Confidentiality 81
14.6 Study Discontinuation 82
14.7 Future Use of Stored Specimens 82
15 Data Handling and Record Keeping 83
15.1 Data Management Responsibilities 83
15.2 Data Capture Methods 83
15.3 Types of Data 83
15.4 Timing/Reports 83
15.5 Study Records Retention 84

15.6 Protocol Deviations 84
16 Publication Policy 85
17 Literature References 86
SupplementS/Appendices 87

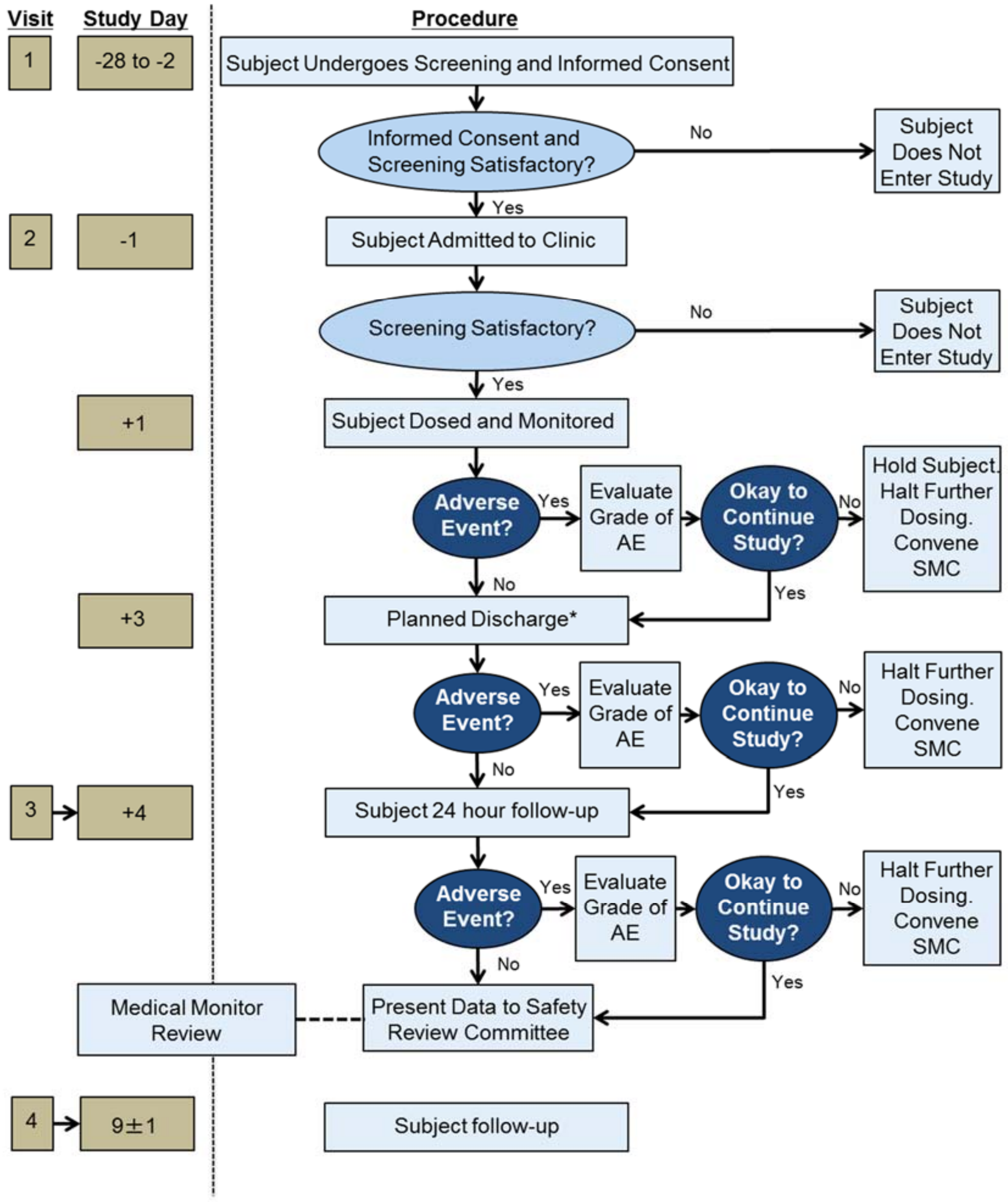
Visit
Study Day
Procedure
1
-28 to -2
Subject Undergoes Screening and Informed Consent
Informed Consent and Screening Satisfactory?
No
Subject Does Not Enter Study
Yes
2
-1
Subject Admitted to Clinic
Screening Satisfactory?
No
Subject Does Not Enter Study
Yes
+1
Subject Dosed and Monitored
Adverse Event?
Yes
Evaluate Grade of AE
Okay to Continue Study?
No
Hold Subject. Halt Further Dosing. Convene SMC
No
Yes
+3
Planned Discharge*
Adverse Event?
Yes
Evaluate Grade of AE
Okay to Continue Study?
No
Halt Further Dosing. Convene SMC
No
Yes
3
+4
Subject 24 hour follow-up
Adverse Event?
Yes
Evaluate Grade of AE
Okay to Continue Study?
No
Halt Further Dosing. Convene SMC
No
Yes
Medical Monitor Review
Present Data to Safety Review Committee
4
9±1
Subject follow-up

LIST OF ABBREVIATIONS

AE	Adverse event/adverse experience
A_e	Amount of analyte excreted
ALT	Alanine aminotransferase
aPTT	Activated partial thromboplastin time
AST	Aspartate aminotransferase
$AUC_{(0-inf)}$	Area under plasma concentration-time curve from time zero extrapolated to infinity
$AUC_{(0-last)}$	Area under plasma concentration-time curve from time zero to time of last quantifiable analyte concentration
$AUC_{(0-8)}$	Area under plasma concentration-time curve from time zero to 8 hours post-dose
$AUC_{(0-24)}$	Area under plasma concentration-time curve from time zero to 24 hours post-dose
BID	Twice daily
BL	Baseline
BMI	Body mass index
BSA	Body surface area
CFR	Code of Federal Regulations
CK	Creatine phosphokinase
CL/F	Apparent systemic clearance
CL_r	Renal clearance
C_{max}	Observed maximum plasma concentration
CNS	Central nervous system
CQMP	Clinical Quality Management Plan
CRA	Clinical Research Associate
CRO	Contract Research Organization
CROMS	Clinical Research Operations and Management Support
CYP	Cytochrome P450
DENV	Dengue virus
DHHS	Department of Health and Human Services
DMID	Division of Microbiology and Infectious Diseases
DNJ	I-deoxynojirimycin
DSP	Diastolic blood pressure
eCRF	Electronic Case Report Form
ECG	Electrocardiogram
EDC	Electronic data capture
ER	Endoplasmic reticulum
FDA	Food and Drug Administration
FOS	Cytosolic free oligosaccharides

CONFIDENTIAL
This material is the property of the Unither Virology. Do not disclose or use except as authorized in writing by the study sponsor.

f_e	Fraction of analyte excreted
FSH	Follicle-stimulating hormone
GCP	Good Clinical Practice
GI	Gastrointestinal
GLP	Good Laboratory Practice
HCl	Hydrochloride
HCT	Hematocrit
HDPE	High-density polyethylene
HED	Human-equivalent dose
HGB	Hemoglobin
HIV	Human immunodeficiency virus
IB	Investigator’s Brochure
IC_{50}	50% inhibitory concentration
ICF	Informed Consent Form
ICH	International Conference on Harmonization
IND	Investigational New Drug Application
INR	International normalized ratio
IRB	Institutional Review Board
LDH	Lactate dehydrogenase
MedDRA®	Medical Dictionary for Regulatory Activities
MOA	Mechanism of action
MTTD	Mean time to death
N	Number (typically refers to subjects)
NIAID	National Institute of Allergy and Infectious Diseases
NIH	National Institutes of Health
NOAEL	No observed adverse effect level
NOEL	No observed effect level
NPC	Niemann-Pick disease
PI	Principal Investigator
PK	Pharmacokinetics
PLT	Platelet
PT	Prothrombin time
SAE	Serious Adverse Event/Serious Adverse Experience
SBP	Systolic blood pressure
SD	Standard deviation
SMC	Safety Monitoring Committee
SOP	Standard operating procedure
SRC	Safety Review Committee
TID	Three times a day, every 8 hours
$t_{1/2}$	Terminal half life
λ_z	Terminal rate constant

t_{max}	Time to reach maximum plasma concentration
US	United States
V_z/F	Apparent volume of distribution during the terminal phase (extravascular)

CONFIDENTIAL
This material is the property of the Unither Virology. Do not disclose or use except as authorized in writing by the study sponsor.

PROTOCOL SUMMARY

**Title:**	RANDOMIZED, DOUBLE-BLIND, PLACEBO-CONTROLLED, PARALLEL GROUP, SINGLE-ASCENDING DOSE STUDY TO DETERMINE THE SAFETY, TOLERABILITY AND PHARMACOKINETICS OF UV-4B SOLUTION ADMINISTERED ORALLY IN HEALTHY SUBJECTS
**Protocol Number:**	DMID 13-0001
**Phase:**	1A
**Population:**	Healthy male and female subjects of non-childbearing potential between the ages of 18 and 45 years inclusive, with a body mass index (BMI) between 18 and 30 kg/m^2 inclusive, and a minimum body weight of 60 kg, will be enrolled (up to 8 cohorts)
**Number of Sites:**	One Phase 1 study site
**Study Duration:**	Approximately 6 months
**Number of Subjects:**	Approximately 64
**Subject Participation Duration:**	Approximately 5 to 6 weeks: There will be an up to 27-day screening period, followed by 1 residential period from the day before dosing (Day -1) until discharge 48 hours post-dose (Day 3), 1 outpatient visit on Day 4, and one follow-up visit on Day 9 ±1.
**Description of Product:**	UV-4B (hydrochloride [HCl]) salt of UV-4), oral solution, anticipated dose range 3 mg to 1000 mg (of free base) (Appropriate volume of the solubilized drug substance solution will be transferred into an oral dosing cup. Ten mL of the masking agent will be added, and the total volume made up to 30 mL with potable water).
**Placebo:**	Taste masking agent, OraSweet-SF, (10 mL) diluted with potable water (20 mL).
**Objectives:**	Primary: to evaluate the safety and tolerability of a single-ascending oral dose of UV-4B in healthy subjects

CONFIDENTIAL
This material is the property of the Unither Virology. Do not disclose or use except as authorized in writing by the study sponsor.

	Secondary: to determine pharmacokinetic parameters describing absorption and elimination following a single dose of UV-4B in healthy subjects.
**Description of Study Design:**	This is a single-ascending dose study with up to eight cohorts of healthy subjects planned. Each cohort will consist of 8 subjects (6 active, 2 placebo). Within each cohort, subjects will be randomized to receive a single oral dose of UV-4B or placebo under fasted conditions. Two subjects within each cohort (i.e. sentinel group: 1 active/1 placebo) will be dosed at least 48 hours prior to the remainder of the cohort. Safety and available exposure data from all subjects in each cohort will be reviewed by the Safety Review Committee (SRC) after all subjects in a given cohort have completed evaluations on Day 4. If specific safety criteria are met, then subjects may be enrolled into the next higher dose cohort (see Section 9.5).The Safety Monitoring Committee (SMC) will review all safety and exposure data after completion of the third cohort. Additionally, the SMC will be consulted for any specific safety signals at any point during the study, to include dose-related trends within the normal range. All safety and exposure data will be submitted to the Food and Drug Administration (FDA) for review after completion of the fourth cohort (dose limit ≤ 90mg x 1).<br><br>The study will be paused during FDA review of the data for cohorts 1 through 4. Upon approval by the FDA the remaining cohorts will be dosed according to the dosing schedule outlined in the protocol.<br><br>Safety assessments will include telemetry, 12-lead electrocardiogram (ECG) measurements, vital signs, physical examinations, clinical laboratory assessments (hematology, serum chemistry, coagulation, urinalysis, and fecal occult blood), and adverse events (AEs). Pooled blood samples and urine will be collected for PK for 48 hours post-dose (until discharge on Day 3). Plasma and urine samples will be tested to determine the following parameters for UV-4B in subjects:<br><br>• UV-4 plasma and urine concentrations and PK parameters (maximum plasma concentration [C_{max}],

- Time to reach maximum plasma concentration [t_{max}],
- Area under the plasma concentration curve from time zero extrapolated to last quantifiable concentration [$AUC_{(0\text{-}last)}$],
- AUC from time zero extrapolated to infinity [$AUC_{(0\text{-}inf)}$],
- Systemic clearance [CL/F]
- Volume of distribution [V_z/F],
- Terminal half-life [$t_{1/2}$],
- Amount of UV-4 excreted in urine unchanged [A_e],
- Fraction excreted in urine unchanged [f_e],
- Renal clearance [CL_r])

The results of these preliminary safety and PK results will be listed and summarized in a table to assist in future dose calculations. Subjects will return to the clinic on Day 4 for additional safety assessments and laboratory assessments. Subjects will return on Day 9 ± 1 for a final safety assessment follow-up visit. UV-4 plasma and urine concentrations and PK parameters (maximum plasma concentration [C_{max}], time to reach maximum plasma concentration [t_{max}], area under the plasma concentration curve from time zero extrapolated to last quantifiable concentration [$AUC_{(0\text{-}last)}$], AUC from time zero extrapolated to infinity [$AUC_{(0\text{-}inf)}$], systemic clearance [CL/F], volume of distribution [V_z/F], terminal half-life [$t_{1/2}$], amount of UV-4 excreted in urine unchanged [A_e], fraction excreted in urine unchanged [f_e], and renal clearance [CL_r]) and safety results will be listed and summarized as appropriate.

Estimated Time to Complete Enrollment: Approximately 5 months

Schematic of Study Design

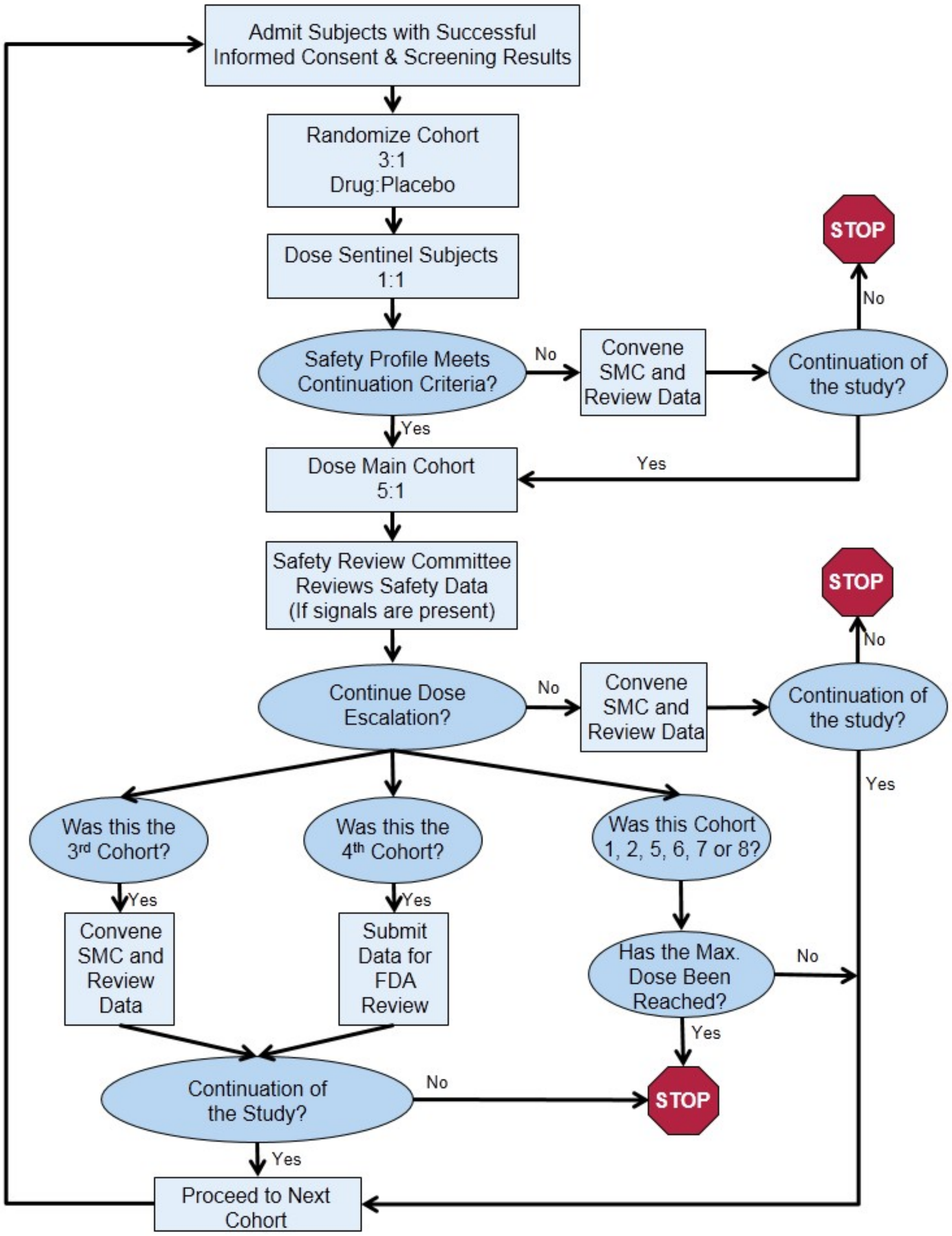

Max = maximum; SMC = safety monitoring committee

1 KEY ROLES

Individuals:

Principal Investigator: Thomas Murtaugh, MD, CPI
Quintiles Phase One Services, LLC
6700 W. 115th Street
Overland Park, KS 66211
Phone: 913-708-6220
Fax: 913-708-7607
Email: Thomas.murtaugh@quintiles.com

Unither Virology Medical Monitor: Michael Callahan, MD, DTM&H, MSPH
President and Chief Medical Officer
Unither Virology; A United Therapeutics Company
1040 Spring Street
Silver Spring, MD 20910
Phone: 202-779-3976
Emergency MGH pager: 617-724-5800 Beeper- 30676
Email: Mcallahan@unither.com

Division of Microbiology and Infectious Diseases (DMID)
DMID Medical Officer: Gregory Deye, MD, CAPT, USPHS
Medical Officer,
Division of Microbiology and Infectious Diseases
National Institutes of Allergy and Infectious Diseases
National Institutes of Health
6610 Rockledge Drive, Room 4506
Bethesda, MD 20892
Phone: 301-443-9383
Email: gregory.deye@nih.gov

DMID Medical Monitor: Mohamed Elsafy, MD, MSc
Medical Monitor/Pharmacovigilance Physician
Office of Clinical Research Affairs
Division Of Microbiology and Infectious Diseases
National Institutes of Allergy and Infectious Diseases
National Institutes of Health
6610 Rockledge Drive, Room 4409
Bethesda, MD 20892
Phone: 301-451-3026
Cell: 301-204-3735

Email: mo.elsafy@nih.gov

Clinical Project Manager: Marianne Baker, RN, MSN
Clinical Project Manager
Office of Biodefense Research Resources and Translational Research
Division of Microbiology and Infectious Diseases
NIAID, NIH
6610 Rockledge Drive, Room 3502
Bethesda, Maryland 20892-6604
Phone: 301-451-6756
Fax: 301-480-1263
Email: bakermr@niaid.nih.gov

DMID Pharmacovigilance Group:
Clinical Research Operations and Management Support (CROMS)
6500 Rock Spring Dr. Suite 650
Bethesda, MD 20817, USA
SAE Hot Line: 1-800-537-9979 (US) or 1-301-897-1709 (outside US)
SAE Fax Line: 1-800-275-7619 (US) or 1-301-897-1710 (outside US)
SAE Email Address: PVG@dmidcroms.com

Data Management: Quintiles, Inc.,
Louretha van Staden, Associate Director
Global Data and Safety Monitoring
196 Nelson Mandela Drive
Brandwag
Bloemfontein 9301, South Africa
Phone: +27 51 401 5370
Fax: +27 51 401 4999
Email: Louretha.Van.Staden@quintiles.com

Clinical Laboratory: Physicians Reference Laboratory
Kimberly Bowers, Project Manager
7800 W. 110th Street
Overland Park, KS 66210
Phone: 913-339-0338
Fax: 913-339-6839
Email: Kimberly.Bowers@prlnet.com

Bioanalytical Laboratory: Quintiles BioServices, Inc.
John Perkins, Director, BioServices, Project Management
19 Brown Road
Ithaca, New York 14950

CONFIDENTIAL
This material is the property of the Unither Virology. Do not disclose or use except as authorized in writing by the study sponsor.

Phone: 607-330-9876
Fax: 607-266-0749
Email: john.perkins@quintiles.com

Institutions: **Quintiles Phase One Services, LLC**
6700 West 115th Street
Overland Park, KS 66211
Phone: 913-708-7555
Fax: 913-708-7607

CONFIDENTIAL
This material is the property of the Unither Virology. Do not disclose or use except as authorized in writing by the study sponsor.

2 BACKGROUND INFORMATION AND SCIENTIFIC RATIONALE

2.1 Background Information

The causative agent of dengue fever is Dengue Virus (DENV), a member of the flavivirus genus. There are four DENV serotypes. Infection with one serotype results in lifelong immunity against that serotype, but only limited short-term cross-protection from infection with the other serotypes. Immunity to one serotype has a downside as subsequent infections by other serotypes increase the risk of developing more severe forms of dengue, which includes the most lethal form of the disease, dengue hemorrhagic fever. Traditional epidemiologic and serologic-based estimates suggest a range of 50 to 100 million DENV infections per year distributed over 100 countries (Guzman et al; 2010, Simmons et al; 2012). Recent cartographic-based modeling studies suggest that up to 390 million (95% credible interval of 284 to 528 million) dengue infections per year, of which 96 million are associated with clinical symptoms (Bhatt et al; 2013). UV-4B is an iminosugar compound salt that is being developed as a broad-spectrum antiviral agent. The initial target indication is for the treatment of DENV infection. The active component of the UV-4B HCl salt is the free base UV-4. UV-4 has a chemical structure consisting of a 1-deoxynojirimycin (DNJ) head group and a 9-carbon methoxy alkyl side chain attached to the nitrogen of the DNJ ring. In the studies summarized below concentrations and doses of UV-4B are expressed as the active free base form, UV-4.

A high-level summary of pharmacology, PK, and safety/toxicology is provided below. For further information, see the Investigator’s Brochure (IB).

2.1.1 Pharmacology

The proposed antiviral mechanism of action (MOA) for DNJ iminosugar analogs is through inhibition of endoplasmic reticulum (ER) α-glucosidases, leading to misfolding of viral glycoproteins on the viral envelope. Misfolded glycoproteins impair virion maturation and translocation to the cell membrane and likely interfere with virus-cell interactions when released. Since the MOA is via disruption of host glycosylation pathways that are exploited by the virus, it is anticipated that development of viral resistance to UV-4B is unlikely (at least through currently known mechanisms of acquired antiviral resistance).

The inhibition of α-glucosidases results in increased levels of misfolded proteins exported from the ER and increased levels of cytosolic free oligosaccharides (FOS). UV-4B was evaluated against purified rat liver ER α-glucosidases I and II. The *in vitro* 50% inhibitory concentrations (IC_{50}) values were 0.16±0.03 μM (51.1±9.6 ng/mL), 0.97±0.20 μM (310±64 ng/mL), and 1.40 μM±0.14 (447±45 ng/mL) for inhibition of purified rat ER α-glucosidase I (Glc3 substrate), rat

ER α-glucosidase II (Glc2 substrate), and rat ER α-glucosidase II (Glc1 substrate), respectively. UV-4B inhibition of α-glucosidase I and II and mouse RAW264.7 cells, rat NRK-49 Fcells, Madin-Darby Canine Kidney cells, and human HL60 cells followed similar kinetics of FOS production and sensitivity to inhibition of ER α-glucosidases.

In vitro IC_{50} values against multiple DENV isolates, including clinical isolates across four serotypes, were determined using a yield reduction assay and ranged from 2.1 μM (671 ng/mL) against DENV-1 SH29177 to 22.3 μM (7124 ng/mL) against DENV-2 SL 5- 17-04.

Studies in human and mouse primary cells showed that cytokine release, including interferons which are a leading mechanism of innate antiviral response in dengue, did not play a role in the antiviral activity of UV-4.

A series of studies were conducted to determine the minimal effective dose and therapeutic window of oral UV-4B in an antibody-dependent enhancement model of DENV infection (with DENV-2 S221). When the first oral dose of UV-4B was administered 1 hour before infection and dosing continued 3 times a day(TID), every 8 hours for 7 days, the minimal effective dose (90% survival rate) was determined to be 10 mg/kg TID and the 50% effective dose was 5 mg/kg TID. Using multiple, step-wise minimal effective dose and therapeutic window studies in this mouse model, the 50% effective dose was determined to be 20 mg/kg TID, which provides significant levels of protection when administered starting 24 or 48 hours after infection.

UV-4B treatment did not alter the course or magnitude of DENV-specific immunoglobulin M or immunoglobulin G antibody responses after infection, as compared to vehicle-treated controls, indicating that treatment with UV-4B is unlikely to affect the development of acquired immunity in dengue-infected patients.

2.1.2 Nonclinical Pharmacokinetics

Following oral administration in mouse, rat, dog, and ferret, UV-4 was rapidly absorbed with t_{max} generally ranging from 0.25 to 0.5 hours. UV-4 was highly bioavailable in mouse (77% to 84%), rat (average ≥99%), and dog (46% to 91%). UV-4 did not accumulate following twice-daily (BID) or TID dose administration after 14 days in either mouse or dog.

Protein binding is moderate with a tendency towards higher binding at the lowest concentration tested (0.5 or 0.9 μg/mL). Following a 6-hour equilibrium dialysis, protein binding ranged from approximately 43% to 54% bound in mouse, 53% to 79% bound in rat, 37% to 79% bound in the dog, and 50% to 75% bound in human plasma. Blood-to-plasma partitioning of UV-4B appeared to be concentration-independent, but may be species

CONFIDENTIAL
This material is the property of the Unither Virology. Do not disclose or use except as authorized in writing by the study sponsor.

dependent from lowest to highest partitioning in mouse, rat, human, and dog. Mean blood cell partitioning ranged from 29.7% to 37.2% in mouse, 32.1% to 35.1% in rat, 32.7% to 53.3% in human, and 58.6% to 63.8% in dog.

UV-4 appears to be metabolically stable in human, mouse, rat, and dog liver microsomes and hepatocytes. Metabolism was minimal and appears to involve O-demethylation, oxidation, and direct glucuronidation of UV-4. Three metabolites were tentatively identified in mouse and dog hepatocytes, whereas only the O-demethylation and oxidation metabolites, but not the direct glucuronidation product of UV-4, were found in human hepatocytes.

Following a single oral dose, UV-4 appears to be rapidly excreted with $t_{1/2}$ values ranging from 2 hours in ferrets, 3 to 5 hours in mice and rats, and 4 to 9 hours in dogs. In rats, urinary excretion of UV-4 after an oral dose of UV-4B was rapid, with the highest percent dose excreted in the initial 0 to 4 hour period after dosing. Approximately 45% of the administered dose was recovered unchanged in urine over a period of 48 hours.

No clinically relevant direct inhibition of cytochrome P450 (CYP) isoenzymes CYP1A2, CYP2A6, CYP2C8, CYP2C9, CYP2C19, CYP2D6, CYP2E1, CYP3A4/5 [midazolam 1'-hydroxylase] was observed *in vitro*. There was weak direct inhibition of CYP2B6 and CYP3A4/5 [testosterone 6β-hydroxylase]. Estimated IC_{50} values for these two CYPs were greater than 600 µM (191 µg/mL), which exceed UV-4 concentrations expected in humans at the planned ceiling dose. There is no significant potential for metabolism-dependent inhibition of all ten CYPs at the tested conditions.

Toxicokinetic data obtained in mice (25 to 250 mg/kg TID) and dogs (2 to 150 mg/kg TID) indicate that UV-4 is rapidly absorbed, exhibits generally dose-proportional toxicokinetics, and does not accumulate to any meaningful extent over the dose range studied. UV-4 toxicokinetics did not exhibit any significant differences between the genders in either species.

2.1.3 Safety Pharmacology and Toxicology

UV-4B did not inhibit the human ether-a-go-go-related gene potassium channel at UV-4 concentration of more than 20-fold higher than the expected concentration at the planned ceiling dose in humans. There were no adverse treatment-related effects in mice in central nervous system (CNS) assessments up to 250 mg/kg TID (750 mg/kg/day) or respiratory function at 1000 mg/kg given as a single dose. In the cardiovascular assessment in dogs, at 10 mg/kg there were no clinically relevant effects and after 50 or 200 mg/kg there was unformed or liquid feces, decreased arterial pulse pressure as the only change outside the normal range, and an initial increase in diastolic blood pressure (DBP) and decrease in systolic blood pressure (SBP) at 50 and 200 mg/kg followed by an increase in SBP at 200 mg/kg only.

CONFIDENTIAL
This material is the property of the Unither Virology. Do not disclose or use except as authorized in writing by the study sponsor.

The seven-day repeat dose study in rats was conducted at 10 mg/kg/dose TID (30 mg/kg/day), 25 mg/kg/dose TID (75 mg/kg/day), and 60 mg/kg/dose TID (180 mg/kg/day). At 10 mg/kg/dose, mild to moderate clinical hematology and clinical chemistry changes and histopathological changes in the gastrointestinal tract (GI) similar to dogs and mice were observed; therefore this dose exceeded the no observed effect level (NOEL). At 25 mg/kg/dose (TID) there were dose-limiting erosions/ulcerations in the stomach. At 60 mg/kg/dose there were similar findings to mice and dogs but a unique finding of possible histamine-like in-life clinical signs were swollen paws/legs and perioral appearance, and red skin, paws and/or feet. These findings appeared within 120 minutes of treatment with the first or second daily dose 60 mg/kg/dose (TID) on Day 1 or Day 2. This response was transient and self-limiting, and was not observed after Day 2 in either the single or repeated doses.

Fourteen-day studies were conducted in mice and dogs. In dogs at 2 mg/kg/dose TID (6 mg/kg/day), there were mild decreases in platelets (PLT), increases in aspartate aminotransferase (AST) (up to 2.8 x baseline [BL]); and a decrease in thymus weight with lymphocyte depletion. Therefore, this dose exceeded the NOEL. At the no observed adverse event level (NOAEL) of 10 mg/kg/dose TID (30 mg/kg/day), there was an increase in the occurrence of poorly formed and liquid feces; decrease in PLT count and increase in activated partial thromboplastin time (aPTT) (2.2 x BL); increase in AST (up to 9.7 x BL) and alanine aminotransferase (ALT) (up to 2.9 x BL) with an inverted AST/ALT ratio; and a decrease in thymus weight with histologic evidence from hematoxylin and eosin stain suggesting lymphocyte depletion (no cellular debris was noted, however). The inverted AST/ALT ratio and lymphocyte depletion were reversible. Thymus weight decrease in females was reversible but thymus weight decreases in males were not. However, because the apparent thymus weight reductions were not dose-dependent and males showed recovery of microscopic thymus findings, thymus weight differences at the recovery necropsy were considered not toxicologically important. In mice at 25 mg/kg/dose (75 mg/kg/day) there were decreased thymus weights (males), follicular epithelium vacuolation in the thyroid, and lymphoid depletion of the thymus (males). All findings were reversible but a NOEL was not present at this dose level. At the no NOAEL of 300 mg/kg/day, there were clinical hematology, chemistry, and anatomic pathology findings similar to those observed in the dogs. All findings were reversible.

Based on these observations, the NOAEL for UV-4B following 14 days of TID administration in mice is considered to be 300 mg/kg/day. This dose level corresponded to Day 14 mean C_{max} and area under the 8 hour dosing interval [$AUC_{(0-8)}$] values of 32,617 ng/mL and 54,435 ng•h/mL, respectively. Daily exposure at NOAEL [calculated as 3 x $AUC_{(0-8)}$] is estimated at 163,305 ng•h/mL. In dogs, the NOAEL following 14 days of TID administration is considered to be 30 mg/kg/day. At the end of the dosing phase, this dose level corresponded to average (male and female) C_{max} and $AUC_{(0-8)}$ values of 5,133 μg/mL and 10,940 ng•h/mL, respectively. Daily exposure [calculated as 3 x $AUC_{(0-8)}$] is estimated at 32,820 ng•h/mL.

CONFIDENTIAL
This material is the property of the Unither Virology. Do not disclose or use except as authorized in writing by the study sponsor.

UV-4B was evaluated in four *in vitro* genetic toxicology assays (two Ames bacterial reverse mutation, mouse lymphoma assay, and chromosomal aberration in cultured human lymphocytes), and in an *in vivo* (mouse bone marrow micronucleus). UV-4B was not found to be mutagenic or clastogenic in any of these assays

2.2 Rationale for Dose Selection

This is a single-ascending dose study in which UV-4B will be administered for the first time in humans. The study will evaluate the safety, tolerability, and PK of increasing oral doses of UV-4B.

The results from this study will form the basis for the selection of doses of UV-4B to be used in future studies in healthy subjects and in patients diagnosed with uncomplicated DENV infection.

In the studies summarized below, the dose stated refers to the free-base used *in vitro* or administered during *in vivo* experiments.

The proposed doses for Cohorts 1 through 8 will range from 3 mg to 1000 mg of UV-4B (dose stated refers to the free base; see Table 2). A comprehensive review of all safety data was conducted at the completion of Cohort 3 by the SMC. In compliance with FDA restriction, no subjects were dosed above 100mg (or above a mean exposure of 3,000 ng•h/mL) without FDA review and approval of safety data at the completion of Cohort 3.

UV-4B, administered orally TID (intragastric via oral gavage) for a total number of 7 days (first dose administered 1 hour prior to infection) was effective in promoting survival of AG129 mice challenged with DENV. Of the doses evaluated (2.5, 5, 10, 20, and 40 mg/kg TID), the minimal effective dose (survival of 90% to 100%) was determined to be 10 mg/kg TID (UV4-LIA-0002). At 5 and 2.5 mg/kg TID, AG129 mice had a survival rate of 50% and 40%. In subsequent studies to define the therapeutic time window, dosing of UV-4B was initiated at increasing time points before (1 hour) or after (24 and 48 hours) infection. In Study UV4-LIA-0004, 20 mg/kg TID showed a 30%, 60%, and 40% survival rate at 11 days post-infection when administered 1 hour before or 24 and 48 hours after infection, respectively, with a mean time to death (MTTD) of 9.1, 10.5, and 6 days. In a previous higher dose study (Study UV4-LIA-0003b), the treatment group receiving the same dose (20 mg/kg TID) showed survival rates of 100%, 80% and 70% and a MTTD of more than 11 (all surviving at end of study), 7 and 9.3 days, respectively. Based on the results in AG129, mice following administration of 10 and 20 mg/kg TID, UV-4B may have relevant pharmacological activity at human-equivalent dose (HED) as low as 146 to 293 mg/day. Assuming similar PK between Crl:CD1(ICR) mice treated in the Good Laboratory Practice (GLP) 14-day toxicology study (Study UV4-COV-036), linear PK, and lack of impact of viral infection on the PK in AG129 mice, UV-4 may have relevant pharmacological activity at a

CONFIDENTIAL
This material is the property of the Unither Virology. Do not disclose or use except as authorized in writing by the study sponsor.

C_{max} and total daily exposure [$AUC_{(0-24)}$] in the range of 4 to 8µg/mL and 18,000 to 37,000 ng•h/mL, respectively (dose-adjusted PK data from the 25 mg/kg/day dose).

Relevant safety pharmacology and toxicology data are provided in Table 1.

Table 1 Relevant Safety Pharmacology and Toxicology Data

Species and Study	Dose (mg/kg)	Finding	Day 1	Day 8	Day 14	Day 28 (Recovery)
Mouse 14-Day (GLP) study (UV4-COV-036)	25 TID	Decreased thymus weight (M)			↓ (M)	−
		Follicular epithelium vacuolation in thyroid			P	−
		Lymphoid depletion of the thymus (M)			P (M)	−
	100 TID (NOAEL)	Decrease in red cell mass (RBC Count, HGB, and HCT)			↓	−
		Decrease in thymus weight			↓	−
		Lymphoid depletion in thymus			P	−
		Follicular epithelium vacuolation in thyroid			P	−
		Neutrophilic infiltration and erosion/ulcer rectum (F)			P	−
Rat 7-Day non-GLP study (UV4-COV-0045)	10 TID	Reticulocyte count		↓		
		PLT		↓		
		WBC and lymphocyte count (F)		↑		
		AST		↑		
		ALT		↑		
		Decreased mucus cells in stomach		P		
	25 TID	Reticulocyte count		↓		
		PLT		↓		
		WBC and lymphocyte count (F)		↑		
		AST		↑		
		ALT		↑		
		Vacuoles in kidney (F)		P(F)		
		Erosion/ulcer, inflammation, decreased mucus cell in stomach, hyperplasia/hyperkeratosis		P		
	60 TID	Swollen paws/legs and perioral appearance and red skin, paws and/or feet	P	−		
		Reticulocyte count		↓		
		PLT		↓		
		WBC and lymphocyte count (F)		↑		
		AST		↑		
		ALT		↑		
		Vacuoles in kidney		P		
		Erosion/ulcer (M), or decreased mucus cell in stomach		P		
		Extra medullary hematopoiesis decreased in spleen and liver		P		
		Decreased lymphocytes in mesenteric lymph node (F)		P(F)		
Dog 14-Day GLP study	2 TID	PLT			↓	−
		AST			↑	−
		Decreased thymus weight			↓	−

Species and Study	Dose (mg/kg)	Finding	Day 1	Day 8	Day 14	Day 28 (Recovery)
(UV4-COV-0035)		Lymphocyte depletion			↓	−
	10 TID (NOAEL)	Nonformed feces	−	P	P	−
		PLT			↓	−
		Reticulocyte count			↓	−
		aPTT			↑	−
		AST			↑	−
		ALT			↑	−
		Inverted AST/ALT ratio			↑	−
		Decreased thymus weight			↓	↓(M)<br>−(F)
		Lymphoid depletion in thymus, spleen, lymph nodes and GALT			P	−
Dog CV Single dose GLP-study (UV4-COV-0023)	10	No clinically relevant effects	−			
	50	Unformed or liquid feces	P			
		Arterial pulse pressure	↓			
		Diastolic blood pressure	↑			
		Systolic blood pressure	↓			

aPTT = activated partial thromboplastin time; ALT = alanine aminotransferase; AST = aspartate aminotransferase; GALT = gut-associated lymphoid tissue; HGB = hemoglobin; HCT = hematocrit; NOAEL = no observed adverse effect level; PLT = platelet; RBC = red blood cell; TID = three times a day; P = present; − = not present; ↑ = increase in relation to normal reference range/from baseline; ↓ = decrease in relation to normal reference range/from baseline; Shaded area = data not applicable; M=male; F-Female

As a conservative approach, the unit dose administered rather than the total daily dose was used for starting dose determination. Based on body surface area (BSA) scaling to obtain the HED, the dog is the more sensitive of the 2 GLP toxicology species. Multiple-dose toxicology studies in GLP mice (Study UV4-COV-036) and non-GLP rat (Study UV4-COV-0045) showed similar results in both species, with the exception of a potential histamine-like clinical response noted as swollen paws/legs and perioral appearance and red skin observed at higher doses. This appears to be a species-specific response to UV-4B.

Based on the dog NOAEL [10 mg/kg/dose TID] and the appropriate conversion recommended in the FDA guidance (e.g., 60 kg body weight for humans), the HED for the NOAEL of UV-4B in dog is 333 mg TID. Considering that the lowest dose tested in dogs (2 mg/kg TID or expressed as HED, 67 mg TID) was associated with decreases in PLT count (mild), increases in aPTT (very mild), increases in AST (up to 3x BL), and histopathological changes, a 100-fold safety factor was applied to the NOAEL. This equates to a 20-fold safety factor applied to HED of the lowest dose tested in dogs. A starting dose based on 1/100 of the HED at the NOAEL (unit dose) in the dog equates to approximately 3 mg.

The selection of the proposed subsequent UV-4B doses is based on the total daily dose administered in the GLP toxicology studies. It employs an initial rapid dose escalation through

CONFIDENTIAL
This material is the property of the Unither Virology. Do not disclose or use except as authorized in writing by the study sponsor.

the initial 3 dose escalation steps to reach approximately one-fifth of the HED for the dog NOAEL (daily dose) by Cohort 5 with subsequent decreases in the dose-escalation algorithm. The dose escalation scheme proposed is included in the protocol (see Table 2) . A comprehensive review of all safety data was conducted at the completion of Cohort 3 by the SMC. In compliance with FDA restriction, no subjects were dosed above 100mg (or above a mean exposure of 3,000 ng•h/mL) until approval was granted by the FDA after their review of safety data at the completion of Cohort 3.The increase in dose for Cohort 2 will not exceed 3 fold. In this study, the starting dose is set to one one-hundredth of the HED at the dog NOAEL (10 mg/kg unit dose) and one twentieth of the low dose (2 mg/kg unit dose) tested in the dog 14-day toxicology study. Setting the PK exposure cut-off for the second dose (Cohort 2) at one-fourth of the exposure at the 2 mg/kg TID dose (or 6 mg/kg/day) equates to a relative target exposure expected at approximately one twentieth of the HED at the dog NOAEL.

The ceiling dose was set as HED at the NOAEL in dogs (i.e.1000 mg/day). This is expected to correlate to a total exposure (mean of cohort) of approximately 32,820 ng•h/mL based on the AUC $_{(0-24)}$ at the NOAEL in dogs. An alternative calculation for maximum dose could be based on C_{max}. Since the findings in the GLP dog toxicology study are thought to correlate with total exposure rather than C_{max}, the exposure calculation for C_{max} was instead based on the acute response to UV-4B observed in the non-GLP rat PK study (Study UV4-COV-0044). In the non-GLP toxicity (repeat dose) study performed in rats, the first treatment at the highest dose tested (60 mg/kg) induced swollen paws/legs and perioral appearance and red skin. These findings were not present in studies in either dogs or mice and appear to be a rat-specific phenomenon at doses at or above 60 mg/kg. Similar findings were seen in rats following dosing of miglustat, a close analog of UV-4B (Zavesca® [miglustat] pharmacology/toxicology review package February 2003), but no similar findings were seen in humans receiving miglustat at doses of up to 1000 mg TID (FDA Advisory Briefing Book for Miglustat [OGT 918, Zavesca®] in Niemann-Pick Type C Disease New Drug Application 021-348/S-007, Endocrinologic and Metabolic Drugs Advisory Committee [EMDAC], 1 December 2009). The events in rats after dosing with UV-4B did not appear to adversely affect the animals and disappeared even with continued TID dosing. Pharmacokinetic data were not collected in the rat non-GLP toxicology study. A 60 mg/kg/dose in rats had an HED of 580 mg for a 60 kg human. Based on the lack of correlation of the histamine-like response to miglustat seen in rats but not in humans, and the toxicity grading scale for testing of UV-4B which includes assessment of findings related to histamine in humans (i.e. urticaria, tachycardia, hypotension; see Appendix B), this calculated 580 mg dose does not present more adverse effects than the 1000 mg dose ,which has been set as the maximum dose for this study.

CONFIDENTIAL
This material is the property of the Unither Virology. Do not disclose or use except as authorized in writing by the study sponsor.

Table 2 Provisional Doses – Highest Provisional Dose Escalation

Cohort	Escalation Scheme	Dose (mg)
1	Starting dose	3
2	Increase approximately 3x	10
3	Increase 3x	30
SMC review of all safety data		
4	Increase 3x	90
FDA review of all safety data		
5	Increase 2x	180
6	Increase 2x	360
7	Increase 2x	720
8 [b]	Increase <1. 5x	1000

Notes: HED = human-equivalent dose; TID = three times daily
[a] Dose escalation in Cohort 2 is set to not exceed approximately one-fourth of the exposure observed at the 2 mg/kg TID dose in dogs.
[b] The ceiling dose has been set as HED at the NOAEL in dogs (i.e. 1000 mg/day).

Human PK data are available for Cohorts 1 through 7 (doses of 3 mg through 720 mg). Using these data, total exposure [$AUC_{(0\text{-}inf)}$] at the highest planned dose (1000 mg) is predicted to be 45,278 ng•h/mL.

2.3 Potential Risks and Benefits

2.3.1 Potential Risks

At the present time, UV-4B has only been given to a small number of human subjects and therefore potential and theoretical toxicity risks of UV-4B are derived from findings in those 56 subjects, animal toxicology studies, and from the understanding of the mechanism of action of UV-4B. There are currently three iminosugar compounds in clinical use. These are miglustat (Zavesca®) for the treatment of Gaucher's disease, and miglitol (Glyset®) for the treatment of

CONFIDENTIAL
This material is the property of the Unither Virology. Do not disclose or use except as authorized in writing by the study sponsor.

type II diabetes mellitus. In addition, Precose® (acarbose), α-glucosidase inhibitor, is approved for the treatment of type II diabetes mellitus and the iminosugar celgosivir (a pro-drug of castanospermine) has been tested in several clinical trials and is currently in development for the treatment of dengue. Together, these four therapies comprise a significant safety database for the evaluation of this iminosugar class of compounds. Relevant safety findings of these iminosugars are discussed below, together with the nonclinical findings for UV-4B. A comprehensive list of safety findings for different animal species is provided in Table 1.

UV-4B is a closely-related analog of miglustat (N-butyl-Deoxynojirimycin) differing in the length and presence of an ether in the N-alkyl sidechain. Miglustat reduces the biosynthesis of glucosylceramide from ceramide through the inhibition of the enzyme glucosylceramide synthase which prevents excessive accumulation of glucosylceramide, the basis for the pathogenesis of Gaucher's disease. Miglustat also inhibits α-glucosidase I and II, the disaccharidases sucrase and maltase, lactase (weak inhibition), and lysosomal and non-lysosomal glucocerebrosidase. The inhibition of disaccharidases is implicated as the cause of abdominal symptoms and osmotic diarrhea that can complicate miglustat treatment.

A review of publicly available safety data for the approved iminosugar compounds, as well as, celgosivir was performed and compiled with the effects of UV-4B observed in animal studies. The combined list of effects is summarized below and each finding is associated with halting criteria for this study. A complete list of known side effects is presented in Appendix C.

1. Gastrointestinal effects: Several iminosugars approved for human use may cause diarrhea, flatulence, and abdominal bloating and discomfort. The cause of these symptoms is believed to be due to the inhibition of intestinal disacharidases which leads to an osmotic diarrhea. Diarrhea is most severe in the early days of treatment and thus is relevant to the acute use scenario that would typify the treatment of Dengue. In a dog dose-range finding (DRF) study for UV-4B, animals receiving the highest dose (150 mg/kg/dose TID) for seven days had severe GI effects, and at doses of 10 mg/kg/dose TID diarrhea was still apparent; similar findings were observed in the 14-day dog study.
   Increased AST and ALT: UV-4B increased AST and ALT and produced increased AST/ALT ratio in both the dog and rat studies. In dogs this AST/ALT ratio change was seen after a single dose. A NOEL for the AST increases has not been identified in dog. At this time the source of increased AST and ALT is unclear; there is no evidence of impairment of actual hepatic function, and no histopathologic changes were observed in the liver. Similarly, creatine phosphokinase (CK) remained normal and there was no histologic evidence of muscle damage.

CONFIDENTIAL
This material is the property of the Unither Virology. Do not disclose or use except as authorized in writing by the study sponsor.

2. Miglustat does not appear to be associated with AST and ALT elevations in humans with Gaucher's disease, Niemann-Pick disease (NPC), and Fabry's disease (Zavesca SmPC), at doses of 100 mg TID or less. In a study designed to evaluate the effect of miglustat on sperm in healthy male subjects, no AST elevations were seen. However, the NOAEL of miglustat was 20 mg/kg which corresponds to an approximately 5 to 6-fold window to the clinical dose based on HED conversion using BSA.

   The safety of alpha-glucosidase inhibitors has been well studied, in one controlled trial of 212 obese patients treated for 36-months with acarbose, ALT elevations occurred in 9% of acarbose vs. 2% of placebo controls; all abnormal ALT values resolved rapidly with discontinuation of drug (Coniff et al 1994).

   A review focused on safety of 1108 patients treated with acarbose for an average of 6-months demonstrated that the most common adverse events were gastrointestinal pain, diarrhea and flatulence; rates of ALT elevations were no different between acarbose and placebo treated patients (Coniff et al 1997).

   Celgosivir, a pro-drug alpha-I glucosidase inhibitor was assessed for safety and tolerability at doses of 300 mg BID; the study was prematurely discontinued due to grade 3 transaminase, CK, and lactate dehydrogenase (LDH) elevations.

   Intensive monitoring of transaminases, hepatic function, CK and LDH isozymes, amylase, and lipase has been included in this study. Careful consideration of this issue has also been built into the stopping rules.

3. Hematologic and immunologic effects: UV-4B resulted in reduced PLT count, increased aPTT and reduced thymus weight. Lymphoid depletion as demonstrated by histopathology was observed at all doses in the dog. Lymphoid depletion and thymus involution were also seen for miglustat, but not at the NOAEL dose of 20 mg/kg. Miglustat also resulted in reduced PLT counts in studies in patients with NPC and human immunodeficiency virus (HIV). However, the effects were small and not considered clinically significant.

   The changes in PLT count and aPTT are unlikely to be of concern in healthy subjects. The lymphoid depletion is unlikely to be of clinical concern over a short dosing period; however, the possibility that it is a manifestation of cytokine production, for example stimulation of IL-4, needs to be considered. In this study, careful monitoring of PLT count and coagulation parameters (aPTT, prothrombin time [PT]) as well as lymphocytes has been implemented. In addition, brief physical examinations focused on the examination of skin and oropharyngeal mucosa for evidence of bleeding and/or bruising (oral [gums] and cutaneous), as well as queries for gingival bleeding during dental care, will be performed daily.

CONFIDENTIAL
This material is the property of the Unither Virology. Do not disclose or use except as authorized in writing by the study sponsor.

4. Cardiovascular effects: Decreased arterial pulse pressure (although limited with no adverse effect on QTc) was apparent in the dog at a dose of 50 mg/kg of UV-4B, approximately 30-fold the predicted minimum clinically efficacious dose. It is therefore possible that similar effects may be seen in humans at higher dose levels in clinical studies. The mechanism of the cardiovascular effects observed is unclear and further studies in animals, including evaluation of cardiac output, as well as *in vitro* electrophysiologic studies, may be required. Cardiovascular effects were seen in studies with miglustat in rat (myopathy) and monkey (myocarditis, prolonged QRS, and atrio-ventricular block). This, together with published studies, indicates that ceramide may inhibit L-type calcium channels. This study includes frequent monitoring of vital signs (inclusive of orthostatic changes), 12-lead ECGs, and 24-hour telemetry to monitor subjects for cardiovascular effects. A single episode of a14 beat run of asymptomatic monomorphic ventricular tachycardia was noted, via telemetry in one subject (Cohort 2) approximately 39 minutes after being dosed with 10 mg of UV-4B. Telemetry continued for a total of 12 hours and no further abnormalities or alterations were noted. This study is ongoing, further observations will be noted as appropriate.

5. Neurologic effects: Animal studies have raised concerns regarding the neurologic safety of miglustat. This concern is based on 1) data from the preclinical toxicology studies in dogs showing tremor and reduction or absence of corneal reflexes at doses of 105 mg/kg/day given over 4 weeks. Neuro-histologic changes were also noted in 3 animals: in monkeys receiving 750-2000 mg QD for 52 weeks, necrosis was of white matter in males; vascular mineralization of the brain and spinal cord; vacuolization of white matter; CNS histologic abnormalities in dog, rat, and monkey. 2) the importance of glycosphingolipids in the CNS, and 3) neurologic adverse effects seen in humans (including tremor and peripheral neuropathy). Tremors were seen in beagle dogs receiving a single dose of 400mg/kg of UV-4B. All subjects in the Phase I study will undergo daily physical exams which include assessment of neurologic function (e.g., mental status, motor system, and sensory system).

6. Histamine-like response: A possible histamine-like response was noted in rats treated with UV-4B (swollen paws/legs and perioral appearance and red skin). This response was observed following single oral doses or after the first or second daily dose in the multiple-dose toxicology program. The observed findings were transient, did not appear to adversely affect the animals, and were not observed after Day 2 repeated doses. These findings were not present in studies in dogs and mice at higher doses (and exposure) and appear to be a rat-specific phenomenon. Similar findings were seen in rats following dosing of miglustat but no similar findings were seen in humans receiving doses of up to 1000 mg TID. Subjects will continue to be closely monitored for histamine-like responses to UV-4B.

CONFIDENTIAL
This material is the property of the Unither Virology. Do not disclose or use except as authorized in writing by the study sponsor.

7. Reproductive toxicity:

   UV-4B has been evaluated for developmental and reproductive toxicity in fertility and early embryonic development in rats, embryo-fetal development in rats and rabbits, and pre- and postnatal development in rats. In male and female rats given UV-4B at 8, 20, and 50 mg/kg/day before mating and continuing through Gestation Day 13, a decreased pregnancy and fertility indices with decreased viable embryos was observed at 50 mg/kg/day. Males at all doses had decreased sperm motility and count but following a 21-week treatment-free period there was complete recovery in pregnancy and fertility indices However, decrease in testicular weight, sperm motility and count did not show complete recovery at all three doses.In a study designed to identify the upper limit of reproductive toxicity, a dose of 50 mg/kg/day for 12 dayswas associated with an increase in skeletal malformations seen in rat offspring. In a study designed to identify the upper limit of reproductive toxicity, a dose of 50 mg/kg/day for 13 days was associated with skeletal and visceral malformations in rabbit offspring. The rat and rabbit doses are equivalent to 486 and 966 mg/day, respectively, in humans.

In summary, findings that have been identified in the UV-4B toxicology studies so far include effects on the GI tract (including ulcers/erosions), lymphocytes and lymphoid tissue, reduction in PLT count, and increases in transaminases (AST>ALT). Findings were generally consistent across mouse, dog, and rat, apart from the isolated finding of swollen paws/legs and perioral appearance and red skin which was seen only in the rat.

Study DMID 13-0001 includes extensive safety monitoring as well as halting criteria that were developed in response to clinical events seen with other iminosugar based therapies, or events seen in animals treated with UV-4B.

2.3.2 Known Potential Benefits

There are no known benefits to subjects participating in this study.

CONFIDENTIAL
This material is the property of the Unither Virology. Do not disclose or use except as authorized in writing by the study sponsor.

3 OBJECTIVES

3.1 Study Objectives

3.1.1 Primary Objective

The primary objective of the study is to determine the safety and tolerability of a single-ascending oral dose of UV-4B in healthy subjects.

3.1.2 Secondary Objective

The secondary objective of the study is to determine pharmacokinetic parameters describing absorption and elimination following a single dose of UV-4B in healthy subjects.

3.2 Study Outcome Measures

3.2.1 Primary Outcome Measures

The primary outcome measures are:

- Evaluation and occurrence of AEs and serious AEs (SAEs)
- Determination of changes from baseline for vital signs, ECGs, and clinical laboratory tests

3.2.2 Secondary Outcome Measures

The secondary outcome measure is:

- UV-4 plasma and urine concentrations and PK parameters [C_{max}, t_{max}, $AUC_{(0\text{-last})}$, $AUC_{(0\text{-inf})}$, CL/F, V_z/F, $t_{1/2}$, A_e, f_e, and CL_r]

CONFIDENTIAL
This material is the property of the Unither Virology. Do not disclose or use except as authorized in writing by the study sponsor.

4 STUDY DESIGN

This is a Phase 1A, single-center, randomized, double-blind, placebo-controlled, parallel group, single ascending dose study of UV-4B solution administered orally in healthy male and female subjects. Eight cohorts of 8 subjects each (6 active, 2 placebo) are planned.

All safety and exposure data was submitted to the FDA for review after the completion of Cohort 3, which corresponded to the limit of not going above a mean exposure of 3,000 ng•h/mL set by the FDA. The study was paused during review of the data. Upon approval by the FDA the remaining cohorts were dosed according to the dosing schedule outlined in the protocol

Within each cohort, subjects will be randomized to receive a single oral dose of UV-4B or placebo under fasted conditions (fasting from at least 10 hours before dosing until 4 hours post-dose). Two subjects within each cohort (i.e. sentinel group: 1 active/1 placebo) will be dosed at least 48 hours prior to the remainder of the cohort. The PI will make the decision to continue subject dosing if no safety signals are present prior to dosing the remaining subjects within the cohort. Safety and available exposure data from all subjects in each cohort will be reviewed by the SRC (in a blinded fashion) after all subjects in a given cohort have completed evaluations on Day 4 and their lab values are available. If specific safety criteria are met, then subjects may be enrolled into the next higher dose cohort (Section 9.6.2).

Safety assessments will include telemetry, 12-lead ECG measurements, vital signs, physical examinations, clinical laboratory assessments (hematology, serum chemistry, coagulation, urinalysis, occult blood in stool), and adverse events. Blood samples and urine will be collected for PK for 48 hours post-dose until discharge on Day 3. Subjects will return to the clinic on Day 4 for additional safety assessments and blood draws for laboratory assessments. Subjects will return 5 to 7 days after discharge for a follow-up visit. [Note: A discharge on Day 3 is based on the assumption that the effective half-life of UV-4 does not exceed 16 hours, (i.e.,) equal to or greater than 87.5% of UV-4 will be eliminated at the time of discharge. This assumption is supported by the PK data from Cohorts 1 through 7 (doses of 3 mg to 720 mg)].

5 STUDY ENROLLMENT AND WITHDRAWAL

Only subjects who meet all of the inclusion and none of the exclusion criteria prior to dosing will be eligible for enrollment into this study. No exemptions are granted on inclusion/exclusion criteria in DMID-sponsored studies.

5.1 Subject Inclusion Criteria

Subjects may be entered in the study only if all of the following criteria are met:

1. Are capable of understanding and complying with the requirements of the study and have signed the informed consent form (ICF);
2. Healthy male and female subjects between 18 and 45 years of age, inclusive;
3. Females must have a negative urine pregnancy test at screening and serum pregnancy test on admission to the unit, must not be lactating, confirmed at screening and at check-in;
4. Females must meet one of the following criteria at screening:
    - Confirmed to be post-menopausal defined as amenorrhea for at least 12 months following cessation of all exogenous hormonal treatments and with follicle-stimulating hormone (FSH) levels in the laboratory-defined postmenopausal range or;
    - Have documentation of irreversible surgical sterilization by hysterectomy, bilateral oophorectomy, or bilateral salpingectomy, but not tubal ligation or occlusion or;
    - If of child bearing potential (does not meet the criteria stated above), starting at least 14 days prior to the first dose of study product and continuing for at least 3 months after the last dose, willing to use:
        a. hormonal contraception plus barrier contraception (condom or occlusive cap such as a diaphragm or surgical vault cap) AND spermicidal foam/gel/cream/suppository or;
        b. Intrauterine Device plus barrier contraception (condom or occlusive cap such as a diaphragm or surgical vault cap) AND spermicidal foam/gel/cream/suppository.
5. Body mass index between 18 and 30 kg/m^2, inclusive;
6. Minimum body weight of 60 kg;
7. Healthy male subjects should be willing to use barrier contraception during sexual intercourse, e.g., condoms, even if they have had a vasectomy, their partners are postmenopausal, surgically sterile, or are using accepted contraceptive methods as

defined in #5 above, from the first day of dosing until 3 months after the last dose of UV-4B;

8. Willing to adhere to the following prohibitions and restrictions during the course of the study to be eligible for participation: Strenuous exercise (i.e. long distance running > 5km/day, weight lifting, or any physical activity to which the subject is not accustomed) is to be avoided while confined to the Clinical Unit and for at least 72 hours prior to each study drug administration (and the Follow-up visit).
9. Nonsmokers (refrained from any tobacco usage, including smokeless tobacco, nicotine patches, etc., for 3 month prior to the administration of the study product); subjects must have cotinine levels below those measured for smokers (<200 ng/mL).

5.2 Subject Exclusion Criteria

Subjects presenting with any of the following must not be included into the study:

1. History of allergy to drug in the iminosugar class;
2. Treatment with any investigational products or therapies within 30 days (or 5 half-lives, whichever is greater) prior to the first day of dosing;
3. Currently has, or has a history of, disease or dysfunction of the pulmonary, cardiovascular, endocrine, hematologic, neurological, immune, GI, genitourinary, or other body system, that is likely to affect the safety of the subject;
4. Creatinine clearance < 90 mL/min (based on Cockcroft-Gault equation);
5. Proteinuria greater than 30 mg/dL;
6. Grade 1 or higher abnormalities in vital signs to include deviations in temperature, resting heart rate, blood pressure, and respiratory rate (see Appendix B). A second set of values were added to Appendix B for subjects who present with asymptomatic bradycardia at baseline at time of screening (<60 bpm);
7. Abnormalities of physical exam suggestive of conditions that would pose an increased risk to the subject;
8. Abnormal ECGs as read by the automated reader in the ECG machine, however; abnormal readings for the following benign conditions will be acceptable for the study: Sinus bradycardia, sinus arrhythmia, early repolarization, nonspecific ST-T wave pattern or changes, right axis deviation (axis 100 degrees or less), first degree AV block (PR interval less than 210ms), nonspecific intraventricular conduction delay (QSR less than 120ms), indeterminate axis, and short PR interval (where no delta wave).

If an abnormal ECG finding is previously recorded, medically insignificant, and attributed to other causes (i.e. body habitus), PI may elect to enroll patient.

9. Laboratory values outside of normal range at screening or check in on Day -1, however; the following list are not considered exclusionary if values fall outside of normal range:

Low Chemistry Values	High Chemistry Values	UA	Hematology Values	Low Coagulation Values
Alkaline Phosphatase<br>AST<br>ALT<br>GGT<br>LDH<br>Creatinine<br>Phosphorus<br>Bicarbonate<br>Uric Acid<br>CPK<br>Chloride<br>BUN<br>Bilirubin (total, indirect, direct)<br>Lipase<br>Amylase	Chloride<br>Phosphorus<br>CPK less than 400<br>Bicarbonate (CO2)	High or low specific gravity<br>High or Low pH<br>Trace protein, when specific gravity is higher than 1.015<br><br>Mucus<br>Crystals<br>Ketones when blood sugar is normal<br>Hyaline casts<br>Blood (unless greater than 3 RBC/hpf and or CPK greater than 400)<br><br>Nitrite (unless greater than 10WBC/hpf)<br><br>Leukocyte Esterase<br>Epithelial Cells<br>Bacteria < many<br>*Urine Spermatozoa	Basophils<br>Eosinophils<br>Lymphocytes<br>Monocytes<br>Neutrophils<br><br>(Differential percentages, absolute values and rbc indices are not by themselves exclusionary; clinical correlation is recommended).	PT<br>aPTT

*when urine spermatozoa are present, urine protein should be repeated if result is elevated.

10. Any known or expected risk of bleeding, such as, but not limited to:

- laboratory evidence of active bleeding, such as positive fecal occult blood , or positive urinary blood that is more than trace positive for hemoglobin (Day -1);
- history of peptic ulcer, GI bleeding (including hematemesis, melena, or rectal bleeding) or bleeding from hemorrhoids;

CONFIDENTIAL
This material is the property of the Unither Virology. Do not disclose or use except as authorized in writing by the study sponsor.

- history of minor bleeding episodes such as, rectal bleeding (spots of blood on toilet paper), and gingival bleeding within 3 months before the first dose;
- any family history (suspected or documented) of coagulopathy;
- females with a history of dysfunctional uterine bleeding, including history of menorrhagia (heavy menstrual bleeding), metrorrhagia, or polymenorrhea;
- use of anticoagulants (i.e. warfarin or low molecular weight heparin), coagulants, anti-PLT (i.e. clopidogrel) 30 days prior to dosing;

11. Has scheduled any surgical procedure during study participation;
12. History of alcohol and/or drug abuse within 1 year prior to dosing, as judged by the PI and/or has a positive urine drug screen for substances of abuse including at a minimum marijuana, cocaine, methamphetamine, opiates, phencyclidine, barbiturates, benzodiazepines, tricyclic antidepressants, methadone, MDMA (ecstasy), oxycodone, and amphetamines, at screening or check-in. Breath tests will be performed for alcohol;
13. Has donated plasma or blood or intends to within 30 days prior to the first day of dosing;
14. Has received treatment with any medication, either prescription or nonprescription, including dietary supplements or herbal medications, within 14 days prior to dosing and is unable to refrain from any medication during the study period. Exceptions are acetaminophen (not more than 2 g/day), vitamin products at recommended daily doses or hormonal birth control;
15. Has received any known hepatic or renal clearance altering agents (e.g., erythromycin, cimetidine, barbiturates, phenothiazines, or herbal/plant-derived preparations such as St. John's Wort) at any time during a period of 30 days prior to the administration of the study product;
16. Has a positive serology test for HIV antibodies, hepatitis B surface antigen, or hepatitis C virus antibody at screening;
17. Any subject with relevant food allergies (i.e. eggs or other components of standard clinic meals) or is unwilling to comply with diet restrictions;
18. Psychological and/or emotional problems, which would render the informed consent invalid, or limit the ability of the subject to comply with the study requirements.
19. Concurrent enrollment in any other clinical trial within 30 days; or
20. Subject is judged by the PI or Sponsor to be inappropriate for the study.

CONFIDENTIAL
This material is the property of the Unither Virology. Do not disclose or use except as authorized in writing by the study sponsor.

5.3 Treatment Assignment Procedures

5.3.1 Randomization Procedures

A randomization schedule will be prepared by Quintiles Inc. and will be provided to the pharmacy staff at the clinic prior to the start of the study. Each cohort will consist of a sentinel group, i.e., two subjects who are randomized to 1 active/1 placebo and the 6 additional subjects who will be randomized to 5 active/1 placebo.

Randomization numbers will be assigned to subjects sequentially using appropriate blocking, following review of all eligibility criteria prior to initiation of any study product administration, and will start with 1001. Table 3 indicates the planned randomization numbers for this study.

Table 3 Randomization Numbers

Cohort	Randomization Numbers
1	1001-1008
2	2001-2008
3	3001-3008
4	4001-4008
5	5001-5008
6	6001-6008
7	7001-7008
8	8001-8008

Randomization numbers for replacement subjects will increment the number of the withdrawing subject being replaced by 100 (e.g., Subject 1001 would be replaced by Subject 1101).

5.3.2 Masking Procedures

Masking will be carried out by use of a commercial, sugar-free taste-masking agent (OraSweet-SF, Paddock Laboratories, Minneapolis, MN). Prior to dosing, the site pharmacy will solubilize an appropriate weight of UV-4B in water for injection. The weight of UV-4B to be solubilized will be determined based on the number of subjects in that cohort and the planned dose as per Table 2. An additional intermediate dilution may be included for the lower dose cohorts. Appropriate volume of either water

CONFIDENTIAL
This material is the property of the Unither Virology. Do not disclose or use except as authorized in writing by the study sponsor.

(placebo) or the solubilized drug substance solution will be transferred into an oral dosing cup. Ten mL of the masking agent will be added, and the total volume made up to 30 mL with potable water.

5.3.3 Reasons for Withdrawal

Subjects will be informed that they have the right to withdraw from the study at any time for any reason without prejudice to their medical care. Data collected for withdrawn subjects will be evaluated for safety and PK (data permitting).

Subjects may be withdrawn from the study for any of the following reasons:

1. Withdraws consent;
2. Noncompliance with study procedures and/or requirements;
3. Death;
4. Pregnancy;
5. In the opinion of the site PI and/or Sponsor, does not meet eligibility criteria upon admission to unit;
6. Administrative reasons/other.

In all cases of withdrawal, the reason for withdrawal will be recorded. Subjects who are withdrawn from the study will be asked to complete safety assessments including follow up of any AEs prior to termination from the study. Subjects withdrawn for reasons other than AEs, after successful inclusion in the study (randomization and/or dosing), will be replaced.

5.3.4 Blinding Procedures

Study subjects, the principal investigator and study site personnel will remain blinded to all randomization assignments throughout the study. The medical monitor and Unither Virology and Quintiles personnel who are in regular contact with the study site and/or involved with documentation associated with the study will remain blinded to all subject randomization assignments.

Selected individuals not involved in the conduct of the study, including members of the SMC may have access to unblinded data as needed for safety review or other data review.

Pharmacist will remain unblinded during the course of the study.

CONFIDENTIAL
This material is the property of the Unither Virology. Do not disclose or use except as authorized in writing by the study sponsor.

5.3.5 Emergency Unblinding

Emergency unblinding of treatment assignment for a subject may be necessary due to a medical emergency, or any other significant medical event. Should an SAE or other circumstance require the blind to be broken to ensure a subject's safety, the Principal Investigator (PI) should immediately notify Unither Virology (within 24 hours) to discuss the case and reason for unblinding (a written narrative must follow within 48 hours of the event).

Unither Virology Medical Monitor:
Michael Callahan, MD, DTM&H, MSPH
President and Chief Medical Officer
Phone: 202-779-3976
Fax: 202-567-3107
24-hour pager: 617-724-5800 (pager #18307)
Email: Mcallahan@unither.com

If emergency unblinding is required for a medical emergency:

- The PI will make the decision to unblind the treatment assignment for AE's involving individual subjects;
- An unblinding memo with the specific details for breaking the blind, including the rationale for unblinding and specific information regarding the subjects that are to be unblinded;
- The document will be sent out for approval and signature to the principal investigator, medical monitor and or sponsor representative, as appropriate;
- The original signed memo will be kept in the Quintiles investigator site file and a copy of the signed document will be sent to the sponsor;
- Clinical Study Director or delegate will send the signed copy of the unblinding memo to the Quintiles unblinded pharmacist, who will send the randomization treatment to the Principal Investigator;
- The PI will review the unblinding information, and if necessary notify the Medical Monitor and/or Sponsor if the stopping criteria have been met;
- The CPM will notify the study team that unblinding has occurred and will provide a copy of the unblinding memo as appropriate;
- The Clinical study Director will notify the IRB that a request has been sent to the pharmacist to unblind the treatment assignment of a subject. Once available, the results of the unblind will be communicated to the Institutional Review Board (IRB).

CONFIDENTIAL
This material is the property of the Unither Virology. Do not disclose or use except as authorized in writing by the study sponsor.

The treatment assignment is not to be provided to site personnel, including the PI at any time during the conduct of the study, except in the case of an emergency.

Any information regarding a subject being unblinded will be documented in the subjects study records.

5.3.6 Termination of Study

Unither Virology, DMID, and the FDA may terminate the study in the interest of subject safety and welfare. In addition, DMID reserves the right to terminate the study at any time for any other reason. If DMID terminates the study, the PI, IRB, SMC, Unither Virology, and the FDA will be informed, activities will be closed, and a study report will be prepared.

CONFIDENTIAL
This material is the property of the Unither Virology. Do not disclose or use except as authorized in writing by the study sponsor.

6 STUDY INVESTIGATIONAL PRODUCT

6.1 Study Product Description

6.1.1 Acquisition

Study product will be shipped to the clinic upon request by Unither Virology.

6.1.2 Formulation, Packaging, and Labeling

UV-4B for solubilization, 5 g, clinical trial material for the Phase 1 clinical program is manufactured, packaged, labeled, and released by SAI Life Sciences Ltd (SAI). Current GMP activities are carried out at SAI's Unit-IV site, Plot No.s 80-A, 80-B, 81-A & 82, Kolhar Industrial Area, Bidar district, Karnataka, India. The FDA registration number for this site is 3006398900.

Clinical trial material is approximately 5 g of UV-4B drug substance, packaged in containers which match the configuration used for the ongoing and planned UV-4B stability programs. UV-4B is filled directly into clear polypropylene bags without any excipients or processing steps. The bag is filled with inert gas (argon) and closed with a nylon twist tie. This bag is then placed into a black polypropylene bag, the outer bag is filled with inert gas, and twist tie is applied. The double-bagged UV-4B is placed in a high-density polyethylene (HDPE) outer container, filled with inert gas, and the HDPE cap is applied. Each container will be labeled for single use in the Phase 1 Pharmacy.

The sterile water and the OraSweet-SF will be obtained by the Phase 1 Pharmacy.

6.1.3 Product Storage and Stability

Stability studies of UV-4B batches have indicated that there are increases in water content at the International Conference on Harmonization (ICH) accelerated stability storage condition. Aside from moisture uptake, there are no other significant and consistent trends in the stability data reported to date (12 months at 25°C/60% relative humidity and 2-8°C conditions, 6 months at 40°C/75% relative humidity condition). For the study, containers of UV-4B will be shipped and stored under 2-8°C refrigerated conditions under locked and limited access.

CONFIDENTIAL
This material is the property of the Unither Virology. Do not disclose or use except as authorized in writing by the study sponsor.

6.2 Dosage, Preparation, and Administration of Study Investigational Product

At time of use, bulk drug substance will be removed from the single-use container and weighed at the clinic. An amount appropriate to the number of subjects being dosed and the dose to be administered will be solubilized in water for injection in a Class A glass 25 mL or 50 mL volumetric flask with a Teflon cap. Gentle mixing by inversion will be used to solubilize the drug substance and generate a UV-4B stock solution. An appropriate volume of UV-4B stock solution will be removed using a syringe, and transferred into an oral dosing cup with 10 mL of taste-masking agent (OraSweet-SF, Paddock Laboratories, Minneapolis, MN). If required, additional potable water will be added to increase the volume to 30 mL. For initial cohorts on the lowest doses, an intermediate dilution in water may be required to simplify preparation of the dilute solution required for dosing. The water for injection and any glassware or plasticware used for solubilization of the UV-4B and dilution and dosing of the drug substance, will be single-use and sourced by the Quintiles Phase 1 unit. Placebo will be 10 mL of taste masking agent diluted to 30 mL with potable water.

Detailed dose preparation instructions will be provided in the dosing plan.

Subjects will be dosed after a 10-hour fast and will continue to fast until 4 hours after dosing and all required post-dose assessments are completed. Consumption of water will be restricted, other than what is required for study product dilution and rinsing for 1 hour before to 2 hours after dosing.

The date and time of UV-4B administration will be recorded in the subjects' electronic Case Report Form (eCRF).

6.3 Accountability Procedures for the Study Investigational Product

The Research Pharmacist (or designee) will keep a record of the dates and amounts of study product received (including packing slips), the amount dispensed to study subjects, any amount destroyed, and the amount unused. Product accountability will be recorded by the Research Pharmacist on a Product Disposition Record or equivalent document with a separate accountability record for study product and placebo. Upon completion of the study, and the final product accountability, all unused study product will be returned or destroyed per Unither Virology requirements and instructions once the relevant accountability records and monitoring visit report have been submitted to Unither Virology.

6.4 Assessment of Subject Compliance with Study Investigational Product

Study product is administered to the study subjects during the in-clinic phase by appropriately trained and designated clinic personnel. Dose administration information such as method, date/time, complete, or incomplete dosing will be documented in the source documentation and in the eCRF for each subject by the study personnel.

6.5 Concomitant Medications/Treatments

All medications taken by a subject for the 30 days prior to enrollment will be documented in the study record. All concomitant medications that are administered during the study must be used for the treatment of an AE and must be recorded in the eCRF for each subject and in the subject’s source documents. All medications (other than acetaminophen) must be authorized by the clinic PI (or designee).

CONFIDENTIAL
This material is the property of the Unither Virology. Do not disclose or use except as authorized in writing by the study sponsor.

7 STUDY SCHEDULE

7.1 Day -28 to Day -2 (Screening)

After providing written informed consent and Health Insurance Portability and Accountability Act (HIPAA) authorization, each subject will be assigned a screening number and undergo an eligibility screening. The following will be obtained during the screening period (within 28 days prior to administration of the study product):

- Review inclusion and exclusion criteria;
- Obtain medical history including current medical diagnoses, major surgical procedures, and medication history including prescription and nonprescription medication (i.e. dietary supplements or herbal medications taken within 30 days prior to Day 1;
- Obtain history of alcohol use, abuse, or dependence;
- Record demographics including age, gender, ethnicity and race;
- Complete a physical examination including vital signs (systolic and diastolic blood pressure, pulse rate, respiration rate, and oral temperature after being supine for 10 minutes), orthostatic blood pressure and pulse rate (taken after 2 minutes in standing position), height, weight, and calculation of BMI. If abnormal, these measurements may be repeated once;
- Obtain blood samples for clinical chemistry, hematology, coagulation tests, urinalysis, urine pregnancy test (if female), FSH (if post-menopausal), HIV, hepatitis B surface antigen, and hepatitis C antibody;
- Obtain urine sample for drugs of abuse and cotinine; (see Section 8.2.1);
- Obtain breath test for alcohol;
- Obtain 12-lead ECG;
- Distribute a fecal occult blood card with instructions for use and return of card to the clinic on or before Day -1;
- Review concomitant medication usage.

7.2 Enrollment/Baseline

7.2.1 Day -1 (Admission to Clinic; 24 hours prior to dosing)

Eligible subjects will return to the clinic on Day -1 for the following procedures. All physical exams, blood tests and ECGs after subjects have been dosed are obtained at approximately the same time each morning (within 2 hours).

- Review inclusion/exclusion criteria. If screening criteria are satisfied, the subject may be admitted to the clinic.
- Confirm that subject adhered to fasting requirements of at least 10 hours before dosing;
- Confirm that subject adhered to dietary restrictions for previous 72 hours;
- Complete an abbreviated physical examination (general appearance, heart, lungs, skin, and abdomen) and brief neurological examination (mental status, motor system, and sensory system);
- Obtain vital signs (blood pressure, pulse rate, after being supine for 10 minutes, and orthostatic blood pressure and pulse rate (taken after 2 minutes in the standing position), respiratory rate and oral temperature;
- Obtain height and weight;
- Obtain urine sample for drugs of abuse and cotinine;
- Obtain breath test for alcohol;
- Obtain serum pregnancy test (if female);
- Obtain baseline clinical chemistry, hematology, coagulation (including quantitative platelet function test with PFA100), and urinalysis;
- Obtain fecal occult blood assessment from kits sent home at screening (if not already assessed). Must have at least 1 negative and no positive values;
- Obtain 2nd 12-lead ECG (Day -1) on the morning of admission. All remaining in-clinic ECGs are obtained at the same time each day for Day +1 (dosing), Day +2, Day +3, Day +4; Day 9±1 ECG is obtained at time of clinic visit (within a 2 hour window of the previous ECG);
- Telemetry for at least 4 hours while awake;
- Start pre-dose pooled urine collection for PK analysis from -12 to 0 hours;
- Sham dose consisting of masking solution in 240 mL (total volume) of diluent will be administered on Day -1; subjects must be able to tolerate solution and rinse for enrollment into the study;
- Review concomitant medication usage.

7.3 Treatment Period

The specific order of evaluations conducted during the treatment period will be defined by the Phase 1 Clinical Trial System Chronologies.

CONFIDENTIAL
This material is the property of the Unither Virology. Do not disclose or use except as authorized in writing by the study sponsor.

7.3.1 Day 1 (Dosing)

Prior to the administration of study product, the following procedures will be performed:

- Perform brief physical examination focused on the examination of skin and oropharyngeal mucosa for evidence of bleeding and/or bruising (oral [gums] and evidence of hematoma, ecchymosis or petechia) brief neurological examination (mental status, motor system, and sensory system);
- Obtain vital signs (blood pressure, pulse rate, after being supine for 10 minutes, and orthostatic blood pressure and pulse rate (taken after 2 minutes in the standing position), respiratory rate and oral temperature;
- Complete pre-dose pooled urine collection for PK analysis from -12 to 0 hours (final void). Initiate collection of urine for new time period;
- Obtain a triplicate 12-lead ECG;
- Obtain baseline blood sample for PK analysis (t=0), to be taken within 1 hour prior to dosing;
- Telemetry starting at -0.5 hours on Day 1 through at least 12 hours post-dose;
- Randomization.

All subjects will be administered the assigned study product or placebo. The total volume of the study product solution plus the rinse(s) and water will be 240 mL.

The following procedures will be instituted to reduce incomplete or interrupted dosing of subjects:

- In the event of partial dosing, either due to dosing error, or the subject coughing, gagging or spilling solution, the subject will only be followed for safety measurements.
- If subject vomits within 30 minutes of dose, an additional naïve subject will be dosed in the same study arm. The goal of each cohort is to dose 8 subjects, but in the event of incomplete dosing, no more than 10 subjects can be randomized.

<u>Following the administration of study product, the following procedures will be performed:</u>

- Obtain blood sample for PK analysis at 0.5, 1, 1.5, 2, 2.5, 3 hours (±15 minutes), 4, 6, 9, 12, and 18, 24, 36 and 48 hours (± 30 minutes) post-dose;
- Obtain pooled urine collection for PK analysis from 0 to 6, 6 to 12, and 12 to 24 hours; and 24 to 48 hours;
- Obtain clinical chemistry, hematology, and coagulation (including quantitative platelet function test with PFA100) subset at 6 and 12 hours (± 30 minutes) post-dose;

- Obtain vital signs (blood pressure and pulse rate including oral temperature and respiratory rate) at 1, 2, 3 hours (±15 minutes), 4, 6, 9, 12, and 24 hours (±30 minutes) and after ECG recording. An additional earlier assessment may be included after Cohort 1, if t_{max} is ≤1 hour. Vital signs should be taken after being supine for 10 minutes, and orthostatic blood pressure and pulse rate (taken after 2 minutes in the standing position);
- Obtain single 12-lead ECGs at the times specified in Appendix D;
- Telemetry through at least 12 hours post-dose;
- Obtain fecal occult blood assessment starting approximately 6 hours post-dose on every stool sample. Stool volume and frequency will be recorded;
- Perform brief physical examination focused on the examination of skin and oropharyngeal mucosa for evidence of bleeding and/or bruising (oral [gums] and cutaneous);
- Review concomitant medication usage;
- Completed AE assessment (including SAEs) will be recorded on AE source forms throughout the study and placed in the subject's clinic records.

Subjects will receive standardized meals as follows on Day 1: lunch 4 hours post-dosing and dinner 9 hours post-dosing (after completion of procedures scheduled at those times); subjects will also receive a light evening snack at approximately 9:00 pm A drink will also be provided with each of the meals. No breakfast will be served on Day 1.

7.3.2 Day 2 In-Clinic Follow-up

The following procedures will be performed:

- Brief physical examination focused on the examination of skin and oropharyngeal mucosa for evidence of bleeding and/or bruising (oral [gums] and cutaneous); symptom directed/targeted examination based on subject symptoms and brief neurological examination (mental status, motor system, and sensory system);
- Obtain vital signs (blood pressure, pulse rate), after being supine for 10 minutes, and orthostatic blood pressure and pulse rate (taken after 2 minutes in the standing position), respiratory rate and oral temperature at 24 hours post-dose;
- Obtain clinical chemistry, hematology, coagulation (including quantitative platelet function test with PFA100), and urinalysis;
- Obtain fecal occult blood assessment on every stool sample or less frequent as per discretion of the PI. Stool volume and frequency will be recorded;
- Obtain 12-lead ECG at 24 hours post-dose;

- Obtain blood sample for PK analysis at 24 and 36 hours post-dose;
- Begin pooled urine collection for PK analysis from 24 (Day 2) to 48 hours (Day 3);
- Review concomitant medication usage;
- Completed AE assessment (including SAEs) will be recorded on AE source forms throughout the study and placed in the subject's clinic records. Data from the AE source form will be entered in the EDC system. All AEs will be monitored until they are resolved or determined by the site PI to be medically stable.

7.3.3 Day 3 Discharge from Clinic

The following procedures will be performed:

- Abbreviated physical examination (general appearance, heart, lungs, skin, and abdomen), and brief neurological examination (mental status, motor system, and sensory system). Also perform brief physical examination focused on the examination of skin and oropharyngeal mucosa for evidence of for bleeding and/or bruising (oral [gums] and cutaneous);
- Obtain weight;
- Obtain vital signs (blood pressure, pulse rate), after being supine for 10 minutes, and orthostatic blood pressure and pulse rate (taken after 2 minutes in the standing position), respiratory rate and oral temperature; at 48 hours post-dose, and weight;
- Obtain clinical chemistry, hematology, coagulation (quantitative platelet function test with PFA100 will only be performed if changes are observed at the prior assessment), and urinalysis;
- Obtain fecal occult blood assessment prior to discharge if stool available prior to discharge. Subjects may receive a fecal occult blood card per discretion of the PI with instructions for use and return of card to the clinic at a subsequent visit. Stool volume and frequency will be recorded;
- Obtain 12-lead ECG at 48 hours post-dose;
- Obtain blood sample for PK analysis at 48 hours post-dose;
- Complete pooled urine collection for PK analysis from 24 (Day 2) to 48 hours (Day 3);
- Review concomitant medication usage;
- Completed AE assessment (including SAEs) will be recorded on AE source forms throughout the study and placed in the subject's clinic records. Data from the AE source form will be entered in the EDC system. All AEs will be monitored until they are resolved or determined by the site PI to be medically stable;
- Discharge from the clinic, with instructions to return on Day 4.

CONFIDENTIAL
This material is the property of the Unither Virology. Do not disclose or use except as authorized in writing by the study sponsor.

7.3.4 Day 4 (Outpatient Visit)

The following procedures will be performed:

- Abbreviated physical examination (general appearance, heart, lungs, skin, and abdomen) and brief neurological examination (mental status, motor system, and sensory system). Also perform brief physical examination focused on the examination of skin and oropharyngeal mucosa for evidence of for bleeding and/or bruising (oral [gums] and cutaneous);
- Obtain vital signs (blood pressure, pulse rate, after being supine for 10 minutes, respiratory rate and oral temperature;
- Obtain clinical chemistry, hematology, coagulation (quantitative platelet function test with PFA100 will only be performed if changes at previous assessment), and urinalysis;
- Obtain 12-lead ECG;
- Obtain blood sample for PK analysis at 72 hours post-dose. Note: this is an optional sample that may be collected based on SRC decision, if warranted by the PK results from prior cohorts;
- Review concomitant medication usage;
- Completed AE assessment (including SAEs) will be recorded on AE source forms throughout the study and placed in the subject's clinic records. Data from the AE source form will be entered in the EDC system. All AEs will be monitored until they are resolved or determined by the site PI to be medically stable;
- Provide instructions to return on Day 9±1.

7.4 Day 9 ±1 Follow-up and Final Study Visit

The following procedures will be performed:

- Complete a physical examination including vital signs (SBP and DBP, pulse rate, respiration rate, and oral temperature after being supine for 10 minutes), and body weight;
- Obtain serum pregnancy test (females only);
- Obtain clinical chemistry, hematology, coagulation (quantitative platelet function test with PFA100 will only be performed if changes are observed at the prior assessment), and urinalysis;
- Obtain vital signs (blood pressure, pulse rate, after being supine for 10 minutes, respiratory rate and oral temperature);
- Obtain 12-lead ECG;

CONFIDENTIAL
This material is the property of the Unither Virology. Do not disclose or use except as authorized in writing by the study sponsor.

- Fecal occult blood assessment at follow-up will be performed as per discretion of the PI, if positive will continue to follow;
- Review concomitant medication usage;
- Completed AE assessment (including SAEs) will be recorded on AE source forms throughout the study and placed in the subject's clinic records. Data from the AE source form will be entered in the EDC system. All AEs will be monitored until they are resolved or determined by the site PI to be medically stable.

7.5 Early Termination Visit

The clinic will make every effort to perform all assessments outlined for the follow-up visit (Study Day 9±1) if a subject is terminated prematurely from the study. All information collected will be documented in the subjects study records including eCRF.

7.6 Unscheduled Visit

Data from unscheduled visits or procedures (if any) will be captured in the EDC system and presented in data listings. These data will be clearly identified as unscheduled observations.

CONFIDENTIAL
This material is the property of the Unither Virology. Do not disclose or use except as authorized in writing by the study sponsor.

8 STUDY PROCEDURES/EVALUATIONS

8.1 Clinical Evaluations

A medical history including a current medication history will be obtained by interview and medical records if available during screening. Additionally, demographic data including gender, date of birth, race, and ethnic origin will be collected.

A history of alcohol abuse or dependence will be obtained at screening.

A complete physical examination, including vital signs, examination of the skin; head, eyes, ears, nose, and throat; lymph nodes; heart; lungs; abdomen; extremities; and joints will be performed at screening and follow-up. This examination will also include an assessment for stigmata of hepatorenal insufficiency, anemia, coagulopathy, addiction, etc. Breast and genital examinations will not be performed. The complete physical examination will include weight (without shoes and wearing lightest possible clothing). The complete physical examination at screening will also include height and calculation of the BMI and 12 lead ECG. An abbreviated physical examination (general appearance, heart, lungs, skin, and abdomen) and brief neurological examination (mental status, motor system, and sensory system) will be done daily. Additionally, brief examinations focused on skin and mouth for bleeding and/or bruising (oral [gums] and cutaneous) will be performed daily. A targeted physical examination (i.e. hematologic/bleeding and neurological) will be conducted in response to any AE, as determined by the site PI and the grading of the AE will then be determined.

8.2 Laboratory Evaluations

8.2.1 Clinical Laboratory Evaluations

Laboratory assessments will be done at screening, check-in, Days 1, 2, 3, 4, and 9 (±1)unless otherwise noted. The following specific tests will be performed as per Appendix D and Table 4:

Screening:

- Serology will include hepatitis B surface antigen, antibodies to hepatitis C, and antibodies to HIV.

<u>Screening and check in on Day -1:</u>

- Urine: Toxicology screen to include at a minimum marijuana, cocaine, methamphetamine, opiates, phencyclidine, barbiturates, benzodiazepines, tricyclic antidepressants, methadone, ecstasy, oxycodone, and amphetamines;

- Serum: Comprehensive metabolic profile (chem 17), complete hepatic panel to include LDH, GGT, AST, ALT and direct and indirect bilirubin, cotinine and blood alcohol levels and serum fertility panel to include HCG to exclude pregnancy and FSH to confirm menopausal/amenorrheic status (females only);

- Hematologic/coagulation: Complete blood count with differential and quantitative PLTs. Coagulation studies will include INR, PT, aPTT. Samples for select hematology (PLT count), clinical chemistry (AST and ALT) and quantitative platelet function test with PFA100 will also be measured on Day 1 at 6 and 12 hours post dose.

Day -1 and Days 1, 2 and 3:

- Platelet function tests will be performed using industry standard PFA100 to support quantitative platelet assays obtained using automated coulter counter or equivalent industry standard. Whole blood is to be drawn into a sodium citrate tube with a volume of at least 4.5 mL. The unprocessed whole blood sample is to be kept and transported at an ambient room temperature.

Day -1, Days 1, 2 and 3 and at follow up on Day 9 (±1):

- Fecal Occult Blood Assessment: At screening, subjects will be given a fecal occult blood card with instructions for use and return of card to the clinic on or before Day-1. This sample will serve as the pre-dose assessment in case the subject does not produce a stool sample prior to dose administration on Day 1. If any fecal occult blood test is positive during the study period, subject(s) will be evaluated and a test will be performed on all subsequent stool specimens until negative. If fecal occult blood remains positive for one week, subjects will be referred for GI evaluation.

All laboratory assessments will be done by the clinical site's certified laboratory utilizing that clinical laboratory's normal ranges. Laboratory toxicity grading will be determined by use of the FDA Guidance for Industry *Toxicity Grading Scale for Healthy Adult and Adolescent Volunteers Enrolled in Preventive Vaccine Clinical Trials* September 2007 and the *Division of Microbiology and Infectious Diseases (DMID) Adult Toxicity Table*

CONFIDENTIAL
This material is the property of the Unither Virology. Do not disclose or use except as authorized in writing by the study sponsor.

November 2007 or as modified for this study and as appropriate for the administration of UV-4B (Appendix B).

Table 4 Safety Laboratory Tests

Hematology	Chemistry	Coagulation	Urinalysis
Red blood cell count	Alkaline phosphatase	INR[b]	pH
Red blood cell distribution width	ALT[b]	PT[b]	Specific gravity
White blood cell count	AST[b]	aPTT[b]	Glucose
Basophils	Total bilirubin	Platelet function [b]	Ketones
Eosinophils	Indirect Bilirubin		
Lymphocytes	Gamma-glutamyltransferase		Leukocyte esterase
Monocytes	Lactate dehydrogenase[a]		Nitrites
Neutrophils	Blood urea nitrogen		Occult blood
Hematocrit	Creatinine		Protein
Hemoglobin	Uric acid		RBCs/hpf
Mean corpuscular hemoglobin	Sodium		WBCs/hpf
Mean corpuscular hemoglobin concentration	Chloride		Bacteria
Reticulocyte count	Carbon dioxide		Casts
MCV	Potassium		Epithelial cells
Platelet count[b]	Creatine phosphokinase[a], [b]		Mucous threads
	Albumin		Crystals
	Calcium		**Fecal:**
	Magnesium		Occult blood
	Glucose		
	Phosphorous		
	Protein (total)		
	Amylase		
	Lipase		

ALT = alanine aminotransferase; aPTT = activated partial thromboplastin; AST = aspartate aminotransferase; hpf = high-power field; INR = international normalized ratio; MCV = mean corpuscular volume; PT = prothrombin time; RBC = red blood cell; WBC = white blood cell.

[a] If either lactate dehydrogenase or creatine phosphokinase is elevated, iso-enzyme levels will be determined using investigative assays.

[b] Samples for select hematology (PLT count), serum chemistry (AST and ALT), and coagulation (INR, PT, aPTT, and quantitative platelet function test with PFA100) assessments will be collected on Day 1 at 6 and 12 hours post-dose. Quantitative platelet function test with PFA100 assessments will also be

CONFIDENTIAL
This material is the property of the Unither Virology. Do not disclose or use except as authorized in writing by the study sponsor.

collected on Day 2 at 24 hours post-dose and on subsequent days (if changes are observed at the previous assessment).

8.2.2 Special Assays or Procedures

Blood samples for UV-4 PK analysis will be collected in tubes containing K2EDTA as the anticoagulant at the times specified in Appendix D. The sampling times may be modified based on PK results from prior cohorts. A 72-hour sample may be added if the PK data from previous cohorts indicate that UV-4 half-life is sufficiently long to allow quantification of UV-4 over the extended collection interval. The actual date and time of collection of each sample will be recorded on the appropriate section of the eCRF.

Pooled urine samples for PK analysis will be collected at pre-dose (-12 to 0 hr) and from 0 to 6, 6 to 12, 12 to 24, and 24 to 48 hours post-dose.

The total volume of blood that will be drawn from each subject in this study is described in Table 5.

Table 5 Volume of Blood to be Drawn from Each Subject

Type	Laboratory Test	Sample Volume (mL)	Number of Samples	Total Volume (mL)
Safety	Clinical chemistry	8.5	6	51
	ALT and AST only	3.5	2	7
	Hematology	2	6	12
	Coagulation	20.7	6	124.2
	aPTT, PT	2.7	2	5.4
	Platelet count	2	2	4
	Platelet function test	4.5	2	9[b]
	Serology	8.5	1	8.5
	future assays[b]	2	1	2
Pharmacokinetic[a]		2	16	32
When using an indwelling catheter 1.0 mL of blood will be removed prior to sample collection		1	15	15
**Total**		**57.4**	**59**	**270.1**

[a] Includes the optional 72-hour sample.

[b] Blood samples will be stored to allow for the option to test for additional parameters including cytokine analysis.

The total volume of blood drawn per subject during this study will not exceed 450 mL, i.e., the amount taken during a normal blood donation.

CONFIDENTIAL
This material is the property of the Unither Virology. Do not disclose or use except as authorized in writing by the study sponsor.

8.2.3 Specimen Preparation, Handling, and Shipping

8.2.3.1 Instructions for Specimen Preparation, Handling, and Storage

The collected PK blood samples (2 mL) will be placed on ice after blood draw, and within 1 hour centrifuged at room temperature at approximately 3000 revolutions per minute for 15 minutes. Plasma will then be harvested and split into 4 aliquots of approximately 250 µL and stored frozen at -20°C until shipped.

Urine samples (approximately 10 mL) for determination of UV-4 concentrations in urine will be taken from the total volume provided during each collection period. The total volume of each urine collection will be recorded along with the start/stop time for each urine collection interval.

Analysis of plasma and urine for UV-4 will be performed by the qualified bioanalytical laboratory, Quintiles Bioservices, Inc., Ithaca, New York 14950. Instructions for the proper preparation, handling, storage and shipping of specimens will be provided separately in a study-specific laboratory manual (i.e., Biopacket).

8.2.3.2 Specimen Shipment

Details regarding shipment of plasma and urine samples can be found in the laboratory manual.

8.3 Diet, Exercise, and Study Restrictions

Enrolled subjects will be advised to abstain from strenuous exercise starting 96 hours (4 days) prior to dosing on Day 1 and continuing through the in-subject stay. In addition, enrolled subjects will be advised to stop consumption of caffeine-containing beverages (e.g., coffee, tea, cola, energy drinks, and cacao [chocolate]) for 72 hours before dosing and refrain from using them during the in-subject stay(s) of the study. Alcohol consumption will not be allowed during the entire study (from 72 hours before dosing through follow-up).

Subjects will be instructed to maintain a diet that will assist in preventing a false positive reading for fecal occult blood. Such a diet excludes: red meat (no beef, lamb, or liver in any form) and vegetables high in peroxidase (turnips, radishes, and horseradish). Vitamin C is limited to ≤ 250 mg/day. Subjects will be given a diet guideline at screening. Subjects will be advised that if accepted for the study they should follow the dietary guidelines for fecal occult blood 72 hours prior to check-in.

Enrolled subjects will remain in the study unit from the time of admission on Day -1 until discharge from the unit on Day 3. During that period of time they will consume a normal house

CONFIDENTIAL
This material is the property of the Unither Virology. Do not disclose or use except as authorized in writing by the study sponsor.

diet (defined as a standard diet that doesn't allow for substitutions and is received by all subjects) served at standard times (breakfast, lunch, and dinner) and will have access to snacks as permitted. Additional food and drinks that are not permitted 72 hours prior to and during the in-subject treatment period of the study are grapefruit or grapefruit juice, Seville oranges or orange marmalade.

Prior to dosing on Day 1, subjects will not eat any food from 10 hours before dosing to 4 hours after dosing until all required post-dose assessments are completed. Consumption of water will not be restricted except for 1 hour before to 2 hours after dosing (except for approximately 240 mL (total volume) allocated for study product solution plus rinse and water). Lunch, dinner, and an evening snack will be provided.

During the follow-up period (Day 9±1), it is recommended that subjects continue eating a balanced diet, and avoid consumption of caffeine-containing beverages.

CONFIDENTIAL
This material is the property of the Unither Virology. Do not disclose or use except as authorized in writing by the study sponsor.

9 ASSESSMENT OF SAFETY

9.1 Specification of Safety Parameters

Safety is a primary outcome measure.

9.1.1 Adverse Events

Adverse events will be evaluated from time of dose administration through the follow-up visit. Adverse events that have not resolved at the follow-up visit will be followed until resolution or until medically stable.

9.1.2 Vital Signs

Vital signs (after being supine for 10 minutes) including SBP and DBP (mmHg), pulse rate (beats/minute), respiratory rate (breaths/minute), and oral temperature (°C) will be obtained and recorded according to the time points in the schedule of events (Appendix D). Systolic and diastolic blood pressure and pulse rate will also be obtained after 2 minutes standing and recorded according to the time points in the schedule of events (Appendix A). If a vital sign measurement is observed to be abnormal, it may be repeated up to a total of 2 times at 5 to 10 minutes intervals. If a normal value is obtained, no further repeat measurements are required. If the value is still abnormal after 2 repeats, the least abnormal value will be utilized for grading the AE. The AE will be considered ongoing until a future measurement shows resolution. The subject may be referred to a primary care provider if the abnormality does not resolve and, according to the study clinicians, requires medical care. The grading of vital sign AEs will be according to toxicity grading criteria listed in Appendix B.

9.1.3 Electrocardiograms

A 12 lead ECG will be performed at approximately the same time each day. Subjects will be placed in a supine position for at least 10 minutes prior to recording an ECG. Triplicate 12 lead ECGs will be collected at the pre-dose on Day 1 (baseline) and a single ECG will be collected at other scheduled times. The screening and Day -1 ECG will be used by the PI to determine subject's eligibility for enrolment. The Day 1 ECG will be reviewed to determine if subjects are safe and appropriate to dose and will be used to determine baseline for waveform and QTc.

CONFIDENTIAL
This material is the property of the Unither Virology. Do not disclose or use except as authorized in writing by the study sponsor.

ECGs will be reviewed by PI or designee. The machine interpretation will be reviewed for accuracy and incorrect findings will be lined out on the tracing and not recorded in the CRF (and correct reading substituted if necessary). ECGs with the following, even when reported by the ECG machine as “abnormal”, will be considered acceptable for study and will not be considered abnormal for study purposes unless the PI or physician delegate believes they represent a new medical condition when compared to baseline ECGs. In such cases an adverse event will be documented and the subject will be followed for safety evaluations as appropriate. The following pre-existing conditions identified at enrollment will not be considered adverse events:

- sinus bradycardia;
- sinus arrhythmia;
- early repolarization;
- nonspecific ST-T wave pattern or changes, mild or moderate right axis deviation;
- first degree AV block (PR interval less than 210ms is acceptable);
- nonspecific intraventricular conduction delay (QRSD, or average of triplicate QRSD; less than 120ms is acceptable);
- short PR interval (where no delta wave), and indeterminate axis.

Automated 12 lead ECG readings describe findings using a variety of terms based on the algorithm. As such, the above terms are not intended to be presented verbatim as machine readings vary slightly between different models and manufacturers. Only similar terms expressing identical concepts written on ECG tracing by the machine will be considered as equivalent to those mentioned above and acceptable for study. The sponsor may review all or individual screening or Day -1 ECGs (or may delegate task to medical monitor) to determine if meets intention of entry criteria goal of enrolling healthy normal study subjects.

Three ECG tracings are made pre-dose. At least 2 of the 3 tracings should be acceptable/normal per (correct) machine reading and all three must be consistent with otherwise healthy normal individuals.

If a post dose ECG demonstrates a prolonged QTc interval (>500ms) or an increase of at least 60ms from baseline average, 2 more tracings should be captured within 10 minutes. The averaged QTc value of these 3 will be used for decision making relative to the halting rules described in Section 9.5.

In the event of a prolongation of QTc post dosing, the site will start telemetry monitoring of the subject and capture triplicate 12 lead ECG (3 tracings within a 5 minute period) every 30 minutes until the average for the triplicates has returned within 60ms of the baseline value at which time telemetry maybe discontinued. Persistent (1 hour or more) elevation in QTc

CONFIDENTIAL
This material is the property of the Unither Virology. Do not disclose or use except as authorized in writing by the study sponsor.

(>525ms) should prompt immediate consideration of transfer to appropriate emergency department for further evaluation, monitoring, and/or treatment.

In the event of a change in ECG wave forms consistent with a change in cardiac function or conduction when compared to baseline tracings, or in the event of a subject with possible cardiac related symptoms, repeat ECG should be obtained every 30 minutes until wave form change reverts to baseline appearance, or symptoms resolve, or are determined not to be cardiac in origin. Subjects with cardiac related symptoms associated with ECG changes will be transferred to appropriate emergency department for further evaluation and treatment.

9.1.4 Telemetry

A 12-lead real-time telemetry ECG will be displayed for at least 4 hours on Day -1 while awake and again starting at -0.5 hours on Day 1 through at least 12 hours post-dose. Any clinically relevant changes from baseline (Day -1, 4-hour recording) will be documented by a 12-lead ECG or an appropriate rhythm strip. These findings will be recorded in the subject's eCRF. Telemetry will be monitored by the PI, research nurse, or designee.

9.1.5 Physical Examination

A complete physical examination, including examination of the skin, head, eyes, ears, nose, and throat; lymph nodes, heart; lungs; abdomen; extremities; and joints will be performed at screening and Day 9 (±1) the follow-up visit. Breast, genital, and rectal examinations will not be performed. The complete physical examination at screening will also include height, weight and calculation of the BMI.

An abbreviated physical examination (general appearance, heart, lungs, skin, and abdomen) and abbreviated neurological examination (mental status, motor system, and sensory system) will be done (Day -1 through 3), and on Day 4.

Brief physical examinations focused on examination of skin and oropharyngeal mucosa for evidence of for bleeding and/or bruising (oral [gums] and cutaneous) will be performed on each day while admitted to the clinic (Day -1 through 3) and on Day 4.

Targeted physical examinations will be done at other study time points if an AE is reported or if the PI or designee feels that it is in the best interest of the study subject.

CONFIDENTIAL
This material is the property of the Unither Virology. Do not disclose or use except as authorized in writing by the study sponsor.

9.1.6 Clinical Laboratory Testing

Laboratory testing will be utilized to evaluate safety on an ongoing basis through the treatment and follow-up periods according to Section 8.2. Laboratory reports are sent from Physicians Reference Laboratory to the clinic daily and are filed in the subject charts. Any values outside of the laboratory defined range will be flagged as such by Physicians Reference Laboratory.

9.1.7 Evaluation of Hypersensitivity Reactions

Examples of hypersensitivity reactions include erythema of oropharyngeal and conjuctival membranes, rhinitis, coryza, breathing difficulties including wheezing, dyspnea and stridor, and dermatologic findings to include diffuse pruritus, macular rashes and or vesciculation. Laboratory abnormalities suggestive of hypersensitivity reaction and requiring clinical corroboration include peripheral eosinophilia and urine eosinophils.

Any evidence of hypersensitivity reaction during study requires cessation of dosing pending physician evaluation and clinical corroboration. If hypersensitivity reaction follows dosing, AE or SAE will be recorded in accordance with physical monitoring criteria or laboratory abnormalities noted in Appendix B.

The Phase 1 treatment facility is capable of managing all types of hypersensitivity reactions from HS type I-IV with bedside epinephrine, diphenhydramine, terbutaline and albuterol nebulizers and oral and/or systemic steroids. Quintiles will follow their Standard Operating Procedures (SOPs) for treatment of hypersensitivity reactions. Please see general anaphylaxis protocol (Appendix E).

9.2 Methods and Timing for Assessing, Recording, and Analyzing Safety Parameters

9.2.1 Adverse Events

ICH E6 defines an AE as any untoward medical occurrence in a patient or clinical investigation subject administered a pharmaceutical product regardless of its causal relationship to the study treatment. An AE can therefore be any unfavorable and unintended sign (including an abnormal laboratory finding), symptom, or disease temporally associated with the use of a medicinal (investigational) product. The occurrence of an AE may come to the attention of study personnel during study visits

and interviews of a study recipient presenting for medical care, or upon review by a study monitor.

All AEs including local and systemic reactions not meeting the criteria for “SAEs” should be captured on the appropriate eCRF. Information to be collected includes event description, time of onset, clinician’s assessment of severity, relationship to study product (assessed only by those with the training and authority to make a diagnosis, which would include Medical Doctor, Physician’s Assistant, Nurse Practitioner, Doctor of Osteopathy, or Dentist), and time of resolution/stabilization of the event. All AEs occurring while on study must be documented appropriately regardless of relationship. All AEs will be followed to adequate resolution as determined by the PI.

Any medical condition that is present at the time that the subject is screened should be considered as baseline/pre-existing condition and not reported as an AE. However, if it deteriorates at any time during the study, it should be recorded as an AE.

All AEs must be graded for severity and relationship to study product. Adverse events (AEs) will be graded according to the FDA Guidance for Industry *Toxicity Grading Scale for Healthy Adult and Adolescent Volunteers Enrolled in Preventive Vaccine Clinical Trials September 2007* and the *Division of Microbiology and Infectious Diseases (DMID) Adult Toxicity Table November 2007* as modified for this study (see Appendix B).

For abnormalities NOT found in the toxicity tables the PI will make an assessment of intensity for each AE and SAE

reported during the study and will assign it to one of the following categories:

GRADE 1: An event that is easily tolerated by the subject, causing minimal discomfort and not interfering with everyday activities;

GRADE 2: An event that is sufficiently discomforting to interfere with normal everyday activities;

GRADE 3: An event that prevents normal everyday activities;

GRADE 4: An event that requires an emergency room visit or hospitalization

Relationship to Study Products: The clinician’s assessment of an AE's relationship to the test article is part of the documentation process, but it is not a factor in determining what is or is not reported in the study. If there is any doubt as to whether a clinical observation is an AE, the event will be reported. All AEs must have their relationship to study product assessed using the terms: related or not related. In a clinical trial, the

CONFIDENTIAL
This material is the property of the Unither Virology. Do not disclose or use except as authorized in writing by the study sponsor.

study product must always be suspect. To help assess, the following guidelines will be used.

Related – There is a reasonable possibility that the study product caused the AE. Reasonable possibility means that there is evidence to suggest a causal relationship between the study product and the adverse event. If an alternate etiology is not identified, the AE must be considered related to study product.

Not Related – There is not a reasonable possibility that the administration of the study product caused the event.

- Document AEs from time of dose administration through the final study visit.

9.2.2 Serious Adverse Events

Serious Adverse Event: An AE or suspected adverse reaction is considered "serious" if, in the view of either the PI or Sponsor, it results in any of the following outcomes:

- Results in death;
- Is life-threatening: an AE is considered "life-threatening" if, in the view of either the PI or sponsor, its occurrence places the patient or subject at immediate risk of death. It does not include an AE, had it occurred in a more severe form, might have caused death;
- Requires hospitalization or prolongation of existing hospitalization;
- Results in disability/incapacity;
- Is a congenital anomaly or birth defect; or
- Is considered an important medical event that may not result in death, be life threatening, or require hospitalizations may be considered serious when, based upon appropriate medical judgment they may jeopardize the subject and may require medical or surgical intervention to prevent one of the outcomes listed in this definition. Examples of such medical events include allergic bronchospasm requiring intensive treatment in an emergency room or at home, blood dyscrasias or convulsions that do not result in in-subject hospitalization, or the development of drug dependency or drug abuse.

All SAEs will be:

CONFIDENTIAL
This material is the property of the Unither Virology. Do not disclose or use except as authorized in writing by the study sponsor.

- Recorded on the appropriate SAE eCRF;
- Followed through to resolution by a study clinician;
- Reviewed and evaluated by a study clinician.

Dosing will be paused for any SAE until causality is fully assessed by the PI and SRC. Dosing will cease if the SAE is determined to be either drug-related or unknown, and may resume if the SAE is determined to be not drug-related by the PI and SRC.

- Document SAEs from time of dose administration through the final study visit.

9.2.3 Procedures to be followed in the Event of Abnormal Laboratory Test Values or Abnormal Clinical Findings

The collection of laboratory data is listed in Section 8.2.1. The normal reference ranges utilized in this study are shown in the Physicians Reference Laboratory Protocol Reference Ranges published by Quintiles updated on 03/20/2013. The grading of abnormal clinical findings are based on the FDA Guidance for Industry *Toxicity Grading Scale for Healthy Adult and Adolescent Volunteers Enrolled in Preventive Vaccine Clinical Trials* September 2007 and *Division Of Microbiology And Infectious Diseases (DMID) Adult Toxicity Table November 2007* or as modified for this study (see Appendix B).

Grade 1 to 3 laboratory or clinical abnormalities will be reported and followed as an AE or SAE, as appropriate. Attempts will be made to follow the subject closely to determine the outcome and duration of an event. Additional assessments including the repeat of laboratory tests and ECGs will be performed in an effort to monitor the subject as appropriate. If an event is ongoing at the time of study termination, permission will be requested from the subject to continue follow-up until the site PI or designee deems the event to be resolved, chronic, or the subject's condition to be medically stable. Abnormal ECGs, at the discretion of the PI or recommendation of the SRC will be reviewed by a cardiologist.

9.3 Reporting Procedures

Adverse events will be reported using the designated AE form. All SAEs , medication toxicities, deaths, or hospitalizations (other than elective) occurring following administration of study product will be recorded using the SAE Form and reported to the IRB and Unither Virology Pharmacovigilance (PVG), CROMS, NIAID's designated pharmacovigilance contractor and the Independent Safety Monitor (ISM), by fax in the mandatory time frame, including those events

CONFIDENTIAL
This material is the property of the Unither Virology. Do not disclose or use except as authorized in writing by the study sponsor.

deemed unrelated to the protocol. Each event will be described in detail along with start and stop dates, relationship to investigational product, action taken, and outcome. Adverse events will be assessed in terms of their seriousness, intensity, and relationship to study product. Events that resulted in planned hospital admissions or surgical procedures for an illness or disease that existed before the subject was enrolled in the study or before study product was given, are not to be considered AEs unless they cause the planned hospital admission or surgical procedure to occur at a time other than the planned date.

9.3.1 Serious Adverse Events

Any SAE, including death due to any cause, that occurs to any subject entered into treatment in this study whether or not considered related to the study product, must be reported within 24 hours of knowing of the event to the Sponsor or their representative.

All SAEs must be reported within 24 hours of the PI's knowledge of the event to the Unither Virology by telephone.

Following notification from the PI, Unither Virology will report any suspected adverse reaction that is both serious and unexpected. Unither Virology's Chief Medical Officer will notify FDA, PI (i.e., the PI to whom the Sponsor is providing drug under its INDs) and DMID in an IND safety report of potential serious risks from clinical trials or any other source, as soon as possible, but in no case later than 15 calendar days after the Sponsor determines that the information qualifies for reporting as specified in 21 CFR Part 312.32. Unither Virology will also notify FDA of any unexpected fatal or life-threatening suspected adverse reaction as soon as possible but in no case later than 7 calendar days after the Sponsor's initial receipt of the information. Relevant follow-up information to an IND safety report will be submitted as soon as the information is available. Upon request from FDA, Unither Virology will submit to FDA any additional data or information that the agency deems necessary, as soon as possible, but in no case later than 15 calendar days after receiving the request.

All SAEs designated as "not related" to study product(s), will be reported to the FDA at least annually in a summary format.

CONFIDENTIAL
This material is the property of the Unither Virology. Do not disclose or use except as authorized in writing by the study sponsor.

9.3.2 Reporting of Pregnancy

A pregnancy reporting form will be completed for any female study subject or for any female partner of a male study subject who becomes pregnant following their exposure to study product (Day 1) through 3 months after the last dose of study product. All pregnancies will also be reported as a protocol deviation. The site will maintain contact through at least monthly telephone calls with pregnant study subjects to obtain pregnancy outcome information. The pregnant subject will be followed by monthly telephone calls until 2 months after the birth of the baby or until the end of the pregnancy (in case pregnancy is terminated). Infants born to these study subjects will also be monitored for SAEs for up to 2 months after birth (information regarding SAEs will be captured on the pregnancy reporting form and the SAE form). Pregnancy reporting forms will be limited to collecting data on the following information:

- Prior maternal history including congenital abnormalities or pregnancy complications;
- Estimated date of conception;
- Estimated and actual date of delivery or pregnancy termination;
- Mode of delivery;
- Maternal complications;
- Neonatal complications (i.e. lethal or nonlethal congenital abnormality).

9.4 Type and Duration of Follow-up of Subjects after Adverse Events

The collection of laboratory data will be limited to those parameters listed in Section 8.2.1. Abnormal laboratory test values or abnormal clinical findings such as heart rate will be recorded using the FDA Guidance for Industry *Toxicity Grading Scale for Healthy Adult and Adolescent Volunteers Enrolled in Preventive Vaccine Clinical Trials* September 2007 (with the exception of sodium and potassium [see Section 9.2.1]). However, the grading is modified for sodium and potassium since there is significant overlap between laboratory normal values and Grade 1 and 2 toxicity criteria using this grading scale (Section 9.2.1). The grading of QTcF prolongation will be according to criteria described in Appendix B. All Grade 1, 2, or 3 laboratory or clinical abnormalities will be reported and followed as an AE, whereas those classified as Grade 4 laboratory or clinical abnormalities will be reported and followed in a similar manner as an SAE. Subjects with AEs will be followed closely to determine the outcome and duration of an event.

CONFIDENTIAL
This material is the property of the Unither Virology. Do not disclose or use except as authorized in writing by the study sponsor.

Additional assessments including the repeat of laboratory tests will be performed in an effort to monitor the subject, as appropriate. If an event is ongoing at the time of study termination, permission will be requested from the subject to continue follow-up until the site PI or designee deems the event to be resolved, chronic, or the subject's condition to be medically stable. All AEs will be monitored until they are resolved or are clearly determined to be due to a subject's stable or chronic condition or intercurrent illness (es).

9.5 Dose Escalation and Halting Rules

After each cohort the SRC will evaluate the safety and tolerability data to determine whether dose escalation can proceed as outlined in this protocol, or the study should be halted and the SMC consulted (see Section 9.6). The SRC will not escalate to the next dose level if any of the events described below have been observed, unless advised by the SMC that dose escalation is permitted.

If adverse events or other safety concerns prevent escalation to the next higher dose, the SMC will be consulted to recommend next steps for subject dosing.

General criteria:

- An SAE that is determined to be either drug-related or of unknown causality;
- Three or more subjects across cohorts, with the same Grade 2 event;
- Two or more subjects in any 1 cohort with a Grade 2 event associated with the same organ system (e.g. increased LFTs and increased total bilirubin);
- Any single grade 3 or above finding (with the exception of aPTT as explained below) ;
- Any other findings that, at the discretion of the SRC or PI, indicate that the study should be halted;
- Any symptoms across the cohorts (i.e. paraphasias; visual field changes, etc.)

QTc criteria:

Two or more subjects, within a dose level meet the criteria below:

- PR: >220ms
- QRS: >120ms
- QTcF > 450 ms for men and >470 for women; or

- Change from baseline (average of pre-dose triplicate ECGs): QTcF >60ms.

Decisions are to be based on an average QTcF value of triplicate ECGs. If an ECG demonstrates a prolonged QT interval, 2 more ECGs should be obtained over a 10 minute period. The averaged QTcF values of the 3 ECGs will then be used for decision making.

Laboratory criteria:

- Two or more subjects within a cohort have a Grade 3 increase in aPTT (confirmed on repeat) as defined by the FDA toxicity grading scale as modified for this study and as appropriate for the administration of UV-4B (Appendix B).

Maximum dose criteria:

- Maximum dose planned for this study is 1000 mg.

If any of the above events occur the study will be halted and the SMC will review the available safety data to determine if the study should proceed as planned. If none of the events described above have been observed, dose escalation will proceed according to the protocol requirements. The SMC met after completion of the 3rd cohort. All safety and exposure data was submitted to the FDA for review after the completion of the 3rd cohort. The study was paused during review of the data for Cohorts 1 through 3. Upon approval from the FDA the remaining cohorts were dosed according to the dosing schedule outlined in the protocol per Table 2.

9.6 Safety Oversight (Safety Review Committee, Independent Safety Monitor plus Safety Monitoring Committee)

9.6.1 Independent Safety Monitor (ISM)

The ISM is a physician located near the investigator site with relevant expertise whose primary responsibility is to provide independent safety monitoring in a timely fashion. This ISM is identified by the investigator site and approved by DMID. The ISM will review all SAEs as submitted by the clinical site and other AEs as needed and provide an independent written

assessment to the SRC and DMID for each SAE. The study site will have an ISM with experience in infectious diseases or internal medicine.

9.6.2 Safety Review Committee (SRC)

After each cohort the SRC will evaluate the safety and tolerability data to determine whether dose escalation can proceed as outlined in this protocol, or the study should be halted and the SMC consulted (Section 5.3.5).

The SRC will consist of the following core members:

- PI (Chair, voting member);
- Independent Safety Monitor (voting member);
- Unither Virology Medical Monitor or designee (voting member);
- DMID Medical Monitor or designee (voting member);
- Quintiles Project Manager (non-voting member);
- Quintiles Clinical Study Director (non-voting member);
- Subject Matter Expert may be consulted as required at the discretion of the SRC.

The PI who has the ultimate responsibility for the safety of the subjects will make the final decision to proceed with the next dose (either escalation, re-dose, reduce dose based on the recommendations of the SMC). The Unither Virology Medical Monitor and/or the DMID Medical Monitor also have the authority to determine whether to stop the study. However, progression to the next dose level (escalation, repeat, or reduction) can only occur if agreed by the PI.

The decisions of the SRC if no halting criteria are seen as per protocol criteria will be documented and provided to all the appropriate parties involved with study, including the pharmacist to enable study product preparation for the next scheduled dose administration day.

9.6.3 Safety Monitoring Committee (SMC)

This clinical trial will utilize an SMC, which is an independent group of experts that advises DMID. The primary responsibility of the SMC is to monitor subject safety. The SMC is external to DMID and composed of at least three voting members. The SMC will consist of members with appropriate expertise to contribute to the interpretation of the data from this trial. Its activities will be delineated in a SMC charter that will describe membership, responsibilities, and the scope and frequency of data reviews. The SMC will operate on a conflict-free basis

independently of the study team. DMID or the SMC may convene ad hoc meetings of the SMC according to protocol criteria or if there are concerns that arise during the study. All reviews by the SMC will be performed with blinded data, unless otherwise requested by the SMC chair. If unblinded data are requested, the SMC will review the unblinded data in a closed session.

There was one planned SMC review of all safety and tolerability data after completion of the 3rd cohort. However, the SMC will be consulted on an ad hoc basis when halting criteria are met, when recommendations are sought for clinical findings and to recommend dose escalation.

9.6.4 Food and Drug Administration

This clinical trial was paused after the completion of the 3rd cohort to allow review of all safety and exposure data by the FDA. Upon review and approval by the FDA the remaining cohorts have been dosed according to the dosing schedule outlined in the protocol per Table 2.

CONFIDENTIAL
This material is the property of the Unither Virology. Do not disclose or use except as authorized in writing by the study sponsor.

10 CLINICAL MONITORING

All clinical monitoring activities will be conducted in accordance with ICH/GCP, applicable Parts in Title 21/45 of the CFR, and DMID requirements for the clinical monitoring of the study conducted under Contract No. Phase 1 DMID HHSN272201100030C.

10.1 Site Monitoring Plan

Independent from the Quintiles Clinical Phase I Unit, a Unither Virology designated independent Clinical Research Associate (CRA) will be responsible for the clinical monitoring of the study at the Quintiles Phase I Unit. The CRAs operate independently from Unither Virology and the Quintiles Phase I Unit and abide by the independent monitoring institution SOPs.

The designated CRA will perform scheduled visits to verify that the clinical trial is conducted and data are generated, documented (recorded), and reported in compliance with the protocol, GCP, and the applicable regulatory requirements. A monitoring plan will be developed commensurate with the degree of potential risk to study subjects and the complexity of the study. Reports will be submitted to Unither Virology on monitoring activities. The site will provide direct access to all trial-related source data/documents, and reports for the purpose of monitoring and auditing by the Sponsor and their designees, and inspection by local and regulatory authorities and their designees. The Quintiles Phase I Unit Clinical Research Coordinator will work closely with the CRA and will attempt to address the issues that are raised. The site PI is ultimately responsible for ensuring that the monitor's findings are addressed. The review of study records will be performed in a manner to ensure that subject confidentiality is maintained.

Monitoring procedures outlined in the Unither Virology-approved site monitoring plan will be followed in order to comply with Section 10. Direct access to the on-site study documentation and medical records will be ensured.

CONFIDENTIAL
This material is the property of the Unither Virology. Do not disclose or use except as authorized in writing by the study sponsor.

11 STATISTICAL CONSIDERATIONS

11.1 Study Hypotheses

Not applicable.

11.2 Sample Size Considerations

The sample size chosen for this study is not based on statistical considerations. The number of subjects within each dose group was chosen based on historical experience with safety and tolerance trials. The sample size falls within the range of those used in other studies of this nature.

11.3 Planned Interim Analyses

Initiation of dosing in subsequent cohorts will occur only after assessment of safety and plasma PK parameters from the previous cohort have been completed.

11.3.1 Safety Review

A report with tables and listings of AEs will be provided to the SMC.

11.3.2 Efficacy Review

Not applicable.

11.3.3 Pharmacokinetic Review

Pharmacokinetic parameters will be analyzed as described in Section 11.4.6. The PK data will be reviewed after each cohort.

11.4 Final Analysis Plan

11.4.1 Populations for Analysis

The safety population will be defined as all subjects who receive study product or placebo. The PK population will consist of all subjects who receive active drug and have scheduled post-dose PK measurement without important protocol deviations, violations,

CONFIDENTIAL
This material is the property of the Unither Virology. Do not disclose or use except as authorized in writing by the study sponsor.

or events thought to significantly affect the PK of the drug. Examples of significant protocol deviations, violations, or events include, but may not be limited to vomiting following oral dosing occurring within the time frame of 2 times the median t_{max}, sample processing errors that lead to inaccurate bioanalytical results, and/or inaccurate dosing on the day of PK sampling. In the case of a significant protocol deviation, PK data collected during the affected treatment period will be excluded from the study results. If any subjects have incomplete data, they will not be included.

11.4.2 Randomization and Stratification

Subjects will be analyzed according to treatment administered rather than treatment randomized, as is standard for early clinical studies, due to the focus on safety and pharmacokinetics. No stratification is planned for this study.

11.4.3 Procedures for Handling Missing, Unused, and Spurious Data

All available scheduled data will be included in data listings and tabulations. In addition, unscheduled visit data will be included in data listings only, as appropriate. No imputation of values for missing data will be performed. Percentages of subjects with AEs or laboratory toxicities will be based on non-missing values. Data that are potentially spurious or erroneous will be examined under the auspices of standard data management operating procedures.

11.4.4 Statistical Plan

A formal statistical analysis plan for the analysis and presentation of data from this study will be prepared before database lock. The following is an overview of planned analyses.

11.4.5 Safety

There are no statistical rules for stopping the study; however, halting rules are given in Section 9.5. After completion of the 3rd cohort, a summary report will be provided to the SMC, including tables and listings of AEs for each cohort.

After completion of the 3rd cohort, a summary report of all safety and pharmacokinetic data was provided to the FDA for all available subjects by cohort for review. After review, FDA provided approval to proceed with additional dose cohorts.

CONFIDENTIAL
This material is the property of the Unither Virology. Do not disclose or use except as authorized in writing by the study sponsor.

Safety variables will be tabulated and presented for all subjects in the safety population, grouped by treatment. Placebo subjects will be pooled into a single treatment group for analysis. Exposure to study product and reasons for discontinuation of study treatment will be tabulated.

Safety evaluation of AEs will be based on the incidence, intensity, and type of AEs. Changes in the physical examination findings, vital signs, and clinical laboratory results will be documented on an AE form. Adverse events will be coded using the Medical Dictionary of Regulatory Activities (MedDRA) AE coding system for purposes of summarization. All AEs occurring in the study will be listed in by-subject data listings. Treatment-emergent events will be tabulated, where treatment-emergent is defined as any AE that occurs after administration of the first dose of study product. Treatment-emergent AEs will be tabulated by relatedness to study product and by maximum severity. Deaths, SAEs, and events resulting in study discontinuation will also be tabulated.

Observed values and change from baseline in clinical laboratory parameters will be summarized over the duration of the study. Shift tables will be produced for selected laboratory parameters. Observed values and changes in vital sign parameters and ECG intervals will be summarized over time in a similar fashion to laboratory parameters, and any abnormal values will be tabulated.

Summary statistics for continuous variables will include number of observations, mean, standard deviation (SD), median, minimum and maximum. Categorical variables will be summarized by the number and percent of subjects. No formal statistical analysis of safety outcomes is planned. Additional safety analyses may be determined at any time without prejudice, in order to most clearly enumerate rates of toxicities and to further define the safety profile of the study product.

CONFIDENTIAL
This material is the property of the Unither Virology. Do not disclose or use except as authorized in writing by the study sponsor.

11.4.6 Pharmacokinetics

Pharmacokinetic parameters will be calculated. Pharmacokinetic analysis procedures will follow Quintiles Inc. SOPs. Pharmacokinetic parameters will be calculated by noncompartmental techniques using WinNonlin Professional® Version 5.2 or higher. All calculations for final plasma parameter analysis will be based on actual sampling times. Interim PK analysis will be based on scheduled sampling times. The following single dose plasma parameters will be estimated from the plasma concentration-time data, as appropriate:

C_{max}	Observed maximum plasma concentration
t_{max}	Time to reach maximum plasma concentration
$AUC_{(0-inf)}$	Area under plasma concentration-time curve from time zero extrapolated to infinity
$AUC_{(0-8)}$	Area under plasma concentration-time curve from time zero to 8 hours
$AUC_{(0-last)}$	Area under plasma concentration-time curve from time zero to time of last quantifiable analyte concentration
λ_z	Terminal rate constant
$t_{1/2}$	Terminal half life
CL/F	Apparent systemic clearance
V_z/F	Apparent volume of distribution during the terminal phase

The following single-dose urine PK parameters will be estimated from the UV-4 urine concentration-time data collected, as appropriate:

A_e	Amount of UV-4 excreted in urine unchanged, calculated for each collection interval and cumulatively across intervals
f_e	Fraction of UV-4 excreted in urine unchanged, calculated for each collection interval and cumulatively across intervals
CL_r	Renal clearance

Additional UV-4 PK parameters may be calculated at the discretion of the pharmacokineticist.

Plasma concentrations and plasma and urine PK parameters will be summarized by treatment using descriptive statistics, as appropriate. Figures for the arithmetic mean (and SD) concentration-time data will be presented for all doses on both a linear and semi-logarithmic scale. Individual concentration-time data will be graphically presented on linear and semi-logarithmic scales. Scatter plots of individual and geometric mean PK parameters versus dose will be presented. Additional graphical presentations of PK data may be added at the discretion of the PK analyst.

Dose proportionality of C_{max}, $AUC_{(0\text{-last})}$, and $AUC_{(0\text{-inf})}$ will be assessed graphically and statistically using the power model approach with the logarithm of PK parameters $AUC_{(0\text{-inf})}$ and C_{max} as the dependent variables and the logarithm of the dose as the independent variable: ([$AUC_{(0\text{-inf})}$, $AUC_{(0\text{-last})}$, or C_{max}]=α*dose$^{\beta}$).

11.4.7 Deviation from Original Analysis Plan

Deviations from the formal statistical analysis plan will be noted in the final Clinical Study Report.

CONFIDENTIAL
This material is the property of the Unither Virology. Do not disclose or use except as authorized in writing by the study sponsor.

12 SOURCE DOCUMENTS AND ACCESS TO SOURCE DATA/DOCUMENTS

The PI and clinical site will permit trial-related monitoring, audits, IRB review, and regulatory inspections as requested by FDA, DMID, Unither Virology or their designee, including direct access to source data/documents (e.g., original medical records, laboratory reports, hospital documents, progress reports, signed ICFs in addition to eCRFs).

CONFIDENTIAL
This material is the property of the Unither Virology. Do not disclose or use except as authorized in writing by the study sponsor.

13 QUALITY CONTROL AND QUALITY ASSURANCE

Processes outlined in the DMID-approved Master Clinical Quality Management Plan (CQMP) will be implemented by the clinical site, Quintiles Phase 1 Unit, to ensure the integrity of the clinical data, safety and welfare of human subjects, and that the clinical trial is conducted in accordance with the approved protocol and with the applicable federal regulations and ICH guidelines. Findings from the CQMP activities will be submitted by written report to Unither Virology within five business days.

Unither Virology will audit the clinical site as needed during the course of the study.

CONFIDENTIAL
This material is the property of the Unither Virology. Do not disclose or use except as authorized in writing by the study sponsor.

14 ETHICS/PROTECTION OF HUMAN SUBJECTS

14.1 Ethical Standard

The PI will ensure that this study is conducted in full conformity with the principles set forth in The Belmont Report: Ethical Principles and Guidelines for the Protection of Human Subjects of Research of the US National Commission for the Protection of Human Subjects of Biomedical and Behavioral Research (April 18, 1979) and codified in 45 CFR Part 46 and/or the ICH E6; 62 Federal Regulations 25691 (1997).

14.2 Institutional Review Board

Midlands IRB holds a current US Federal-wide Assurance issued by the Office of Human Research Protections.

Prior to initiation of the study, the site PI will submit the study protocol, sample ICF, and any other documents that pertain to subject information, recruitment materials such as advertisements, to IRB. The site PI must also submit any other information that may be requested to the IRB for review and approval. The site PI will request that the IRB provide written approval of the study and will keep on file records of approval of all documents pertaining to this study. A letter confirming the approval must be forwarded to the CRA prior to initiation of this study. This letter will be forwarded to Unither Virology and DMID prior to the initiation of the study.

The PI must submit and, where necessary, obtain approval from the IRB for all subsequent protocol amendments and changes to the informed consent document. The PI should notify the IRB of deviations from the protocol or SAEs occurring at the site, as well as other AE reports received from Quintiles, in accordance with local procedures.

The PI will be responsible for obtaining annual IRB approval or renewal throughout the duration of the study.

14.3 Informed Consent Process

Informed consent is a process that is initiated prior to the individual's agreeing to participate in the study and continuing throughout the individual's study participation. Extensive discussion of risks of this therapy will be provided to the subjects. Consent forms describing in detail the study products, study procedures, and risks are given to the subject and written documentation of informed consent is required prior to administering study product. Consent forms will be IRB-approved and the subject will be asked to read and review the document. Upon reviewing the

CONFIDENTIAL
This material is the property of the Unither Virology. Do not disclose or use except as authorized in writing by the study sponsor.

document, the PI will explain the research study to the subject and answer any questions that may arise. The subjects will sign the informed consent document prior to any procedures being done specifically for the study. The subjects should have the opportunity to think about the study and discuss with others prior to agreeing to participate. The subjects may withdraw consent at any time throughout the course of the study. A copy of the informed consent document will be given to the subjects for their records. The rights and welfare of the subjects will be protected according to 21 CFR 312.60.

14.4 Exclusion of Women, Minorities, and Children (Special Populations)

Children under age 18 years will be excluded from participation because insufficient data are available in adults to judge potential risk in children, and as a Phase 1 trial, there is no known benefit. For these same reasons, this trial will not include other special classes of subjects, such as fetuses, neonates, prisoners, institutionalized individuals, or others who may be considered vulnerable populations.

Neither women nor minorities will be excluded from participation in this study. Women of non-childbearing potential may be included. Subjects will be recruited without regard to gender or race. It is expected that race will reflect that within the community but that exclusion of women of childbearing potential may lead to disproportional numbers of males.

14.5 Subject Confidentiality

Subject confidentiality is strictly held in trust by the PI, his/her staff, and the Sponsor(s) and their agents. This confidentiality is extended to cover testing of biological samples and genetic tests in addition to the clinical information relating to participating subjects.

The study protocol, documentation, data, and all other information generated will be held in strict confidence. No information concerning the study or the data will be released to any unauthorized third party without prior written approval of the Sponsor.

The study monitor or other authorized representatives of the Sponsor may inspect all documents and records required to be maintained by the PI, including but not limited to, medical records (office, clinic, or hospital) and pharmacy records for the subjects in this study. The clinical study site will permit access to such records.

CONFIDENTIAL
This material is the property of the Unither Virology. Do not disclose or use except as authorized in writing by the study sponsor.

14.6 Study Discontinuation

In the event that the study is discontinued, no further subjects will be dosed with UV-4B.

14.7 Future Use of Stored Specimens

Any plasma, blood, or urine left after all clinical laboratory testing is done will be discarded. Any plasma and/or urine left after PK analysis will be stored at Unither Virology or designee bioanalytical laboratory and used for future research. A separately collected blood sample will be stored at Unither Virology or designated bioanalytical laboratory to allow for the option to test for additional parameters including cytokine analysis. At the completion of the protocol, the disposition of samples and data will be per Unither Virology instructions. Any loss or unanticipated destruction of samples or data will be reported to the IRB. In compliance with subject confidentiality and privacy act (e.g. HIPAA); no genetic testing will be done on any stored specimens.

CONFIDENTIAL
This material is the property of the Unither Virology. Do not disclose or use except as authorized in writing by the study sponsor.

15 DATA HANDLING AND RECORD KEEPING

15.1 Data Management Responsibilities

Any source documents and laboratory reports must be reviewed by the clinical team and data management staff, who will ensure their accuracy and completeness. Adverse events must be graded based on the FDA Guidance for Industry Toxicity Grading Scale for Healthy Adult and Adolescent Volunteers Enrolled in Preventive Vaccine Clinical Trials September 2007, as modified for this study and as appropriate for the administration of UV-4B (Appendix B).

Electronic data capture is the responsibility of the clinical trial staff at the site under the supervision of the site PI. During the study, the PI must maintain complete and accurate documentation for the study.

15.2 Data Capture Methods

Clinical data (including AEs and concomitant medications) will be entered into a 21 CFR Part 11-compliant data management system provided by Quintiles. The data system includes password protection and internal quality checks, such as automatic verification range checks, to identify data that appear to be out of the specified ranges. Programmed edit specifications identify discrepancies in the data that may are addressed by the site.

15.3 Types of Data

Data for this study will include all data for safety and PK deemed necessary for the analysis per the protocol. Externally collected data are received and processed to present a complete dataset reconciled with data collected at the bedside.

15.4 Timing/Reports

Data collected at the bedside are available for review at the time of the collection. Data are collected, reviewed, and queries are issued by data management (per the specifications in the Data Management Plan). The independent CRA will monitor the data and may also create queries for site clarification. Adverse event data and concomitant medication data are coded by the MedDRA and World Health Organization dictionaries. Any SAEs will also be reported through the appropriate processes identified in the Data Management Plan. After the data are deemed “clean” (i.e., no further changes are required), the CRA will approve the subjects’ data by applying an e-signature in the EDC system. Data will then be considered “frozen” and no new data entered into the system. The data will be reviewed by appropriate team members and

the bioanalytical data will be received. Data for this study will include all data for safety and PK deemed necessary for the analysis per the protocol. Externally collected data will be received and processed to present a complete dataset reconciled with data collected at the bedside. The data will be moved to a status of “locked” and the data set transferred to the statistical programming team for creation of tables, figures, and listing review.

15.5 Study Records Retention

Study documents should be retained for a minimum of 2 years after the last approval of a marketing application in an ICH region, and until there are no pending or contemplated marketing applications in an ICH region, or at least 2 years have elapsed since the formal discontinuation of clinical development of the investigational product. These documents should be retained for a longer period, however, if required by local regulations. No records will be destroyed without the written consent of the Unither Virology. It is the responsibility of the Unither Virology to inform the PI when these documents no longer need to be retained upon notification from Quintiles.

15.6 Protocol Deviations

A protocol deviation is any noncompliance with the clinical trial protocol, GCP, or manual of procedures requirements. The noncompliance may be either on the part of the subject, the PI, or the study site staff. As a result of deviations, corrective actions are to be developed and documented by the site and implemented promptly with associated records provided to Unither Virology.

These practices are consistent with ICH E6:

4.5 Compliance with Protocol, Sections 4.5.1, 4.5.2, and 4.5.3;

5.1 Quality Assurance and Quality Control, Section 5.1.1

5.20 Noncompliance, Sections 5.20.1 and 5.20.2.

It is the responsibility of the site to identify and report deviations within 5 working days of identification of the protocol deviation, or within 5 working days of the scheduled protocol required activity. All deviations must be promptly reported to Unither Virology.

CONFIDENTIAL
This material is the property of the Unither Virology. Do not disclose or use except as authorized in writing by the study sponsor.

16 PUBLICATION POLICY

The data generated by this study are confidential information of the Sponsor. The Sponsor will make the results of the study publicly available as stipulated per the National Institute of Health Public Access Policy implements Division G, Title II, Section 218 of PL 110-161 (Consolidated Appropriations Act, 2008) which states:

SEC. 218. The Director of the National Institutes of Health shall require that all investigators funded by the NIH submit or have submitted for them to the National Library of Medicine's Pub Med Central an electronic version of their final, peer-reviewed manuscripts upon acceptance for publication, to be made publicly available no later than 12 months after the official date of publication: Provided, that the NIH shall implement the public access policy in a manner consistent with copyright law.

By signing the study protocol, the PI agrees that the results of the study may be used for the purposes of national and international registration, publication, and information for medical and pharmaceutical professionals by the Sponsor. If necessary, the authorities will be notified of the PI's name, address, qualifications, and extent of involvement.

If the PI, Unither Virology, or Quintiles, Inc. is to be included as an author of a publication manuscript prepared by the Sponsor, the Sponsor will allow the PI, Unither Virolgy, or Quintiles, Inc. 60 days for full review of the manuscript before publication.

CONFIDENTIAL
This material is the property of the Unither Virology. Do not disclose or use except as authorized in writing by the study sponsor.

17 LITERATURE REFERENCES

Bhatt S, Gething PW, Brady OJ, Messina JP, Farlow AW, Moyes CL, et al. The global distribution and burden of dengue. Nature. 2013;496:504-7.

Coniff R, Krol A. Acarbose: a review of US clinical experience. Clin Ther 1997; 19: 16-26.

Coniff RF, Shapiro JA, Seaton TB. Long-term efficacy and safety of acarbose in the treatment of obese subjects with non-insulin-dependent diabetes mellitus. Arch Intern Med 1994; 154: 2442-8.

Guzman MG, Halstead SB, Artsob H, Buchy P, Farrar J, Gubler DJ, et al. Dengue: a continuing global threat. Nat Rev Microbiol. 2010;8(12 Suppl):S7-16.

Simmons CP, Farrar JJ, Nguyen W, Wills B. Dengue. N Eng J Med. 2012;366:1423-32.

Food and Drug Administration [FDA] Advisory Briefing Book for Miglustat [OGT 918, Zavesca®] in Niemann-Pick Type C [NPC] Disease NDA 021-348/S-007, Endocrinologic and Metabolic Drugs Advisory Committee [EMDAC], 2009

SUPPLEMENTS/APPENDICES

Supplements and Protocol Appendices

- *Appendix A: Subject Flowdown*
- *Appendix B: UV-4B Toxicity Grading Criteria for Normal Human Subjects*
- *Appendix C: Iminosugar Clinical Signs*
- *Appendix D: Study of Events*
- *Appendix E: General Treatment Plan for an Anaphylactic Reaction*

Related Documents

- *Site Roster*
- *Manual of Procedures*
- *Repository Instructions*
- *Biosafety Precautions*
- *Laboratory Handling*

Other Documents

- *CRF copies*
- *Quality Management Plan*
- *Data Management Plan*
- *Clinical Monitoring Plan*

APPENDIX A: SUBJECT FLOWDOWN

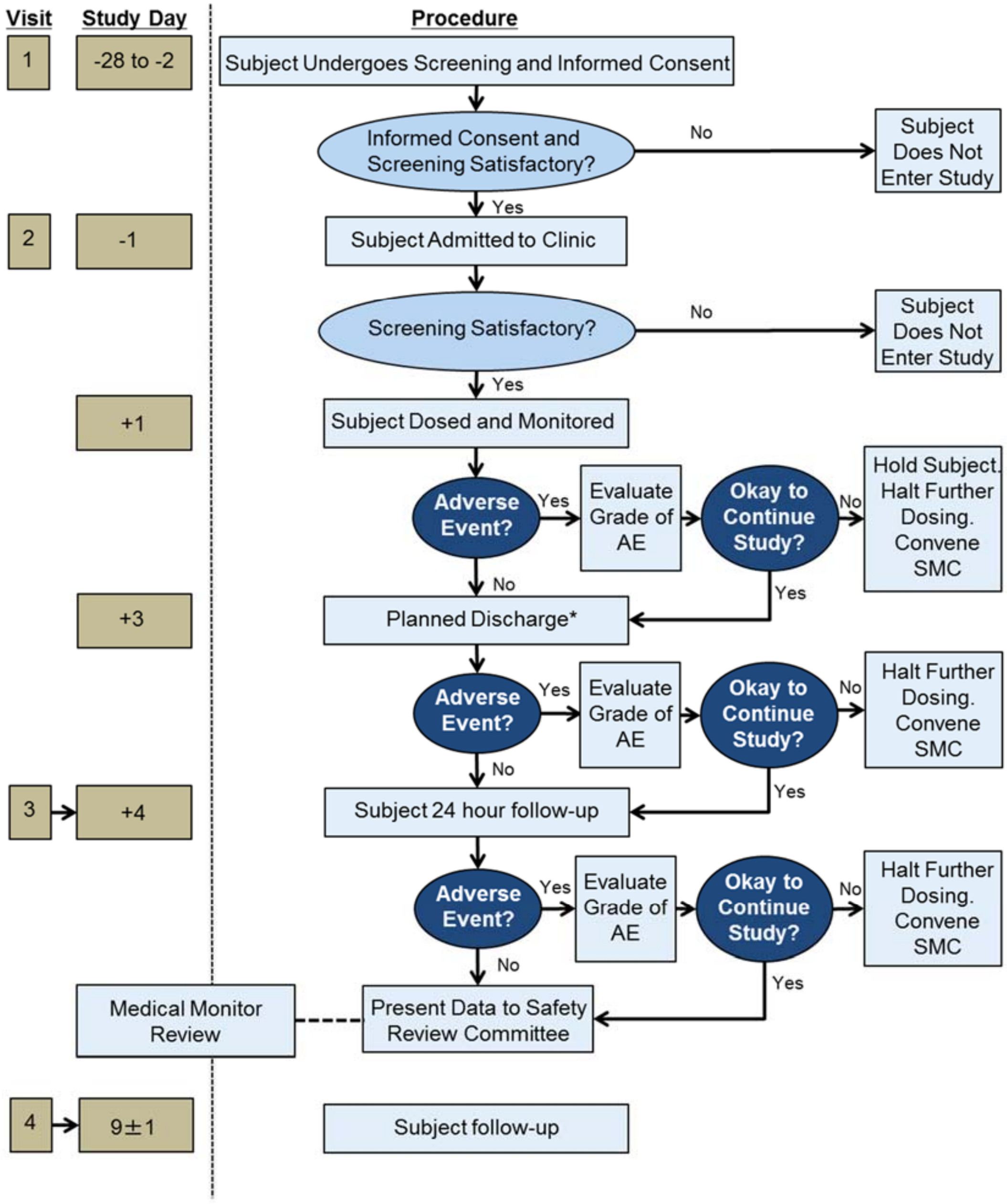

*In case of half-life longer than 16 hours, the in-subject portion of the treatment period may be extended longer than 3 days.

APPENDIX B: UV-4B TOXICITY GRADING CRITERIA FOR NORMAL HUMAN SUBJECTS

Vital Signs*	Mild (Grade 1)	Moderate (Grade 2)	Severe (Grade 3)	Potentially Life Threatening (Grade 4)
Fever (°C)**<br>(°F)**	38.0 – 38.4<br>100.4 – 101.1	38.5 – 38.9<br>101.2 – 102.0	39.0 – 40<br>102.1 – 104	>40<br>>104
Tachycardia – (beats per minute)	101 – 115	116 – 130	>130	ER visit or hospitalization for arrhythmia
Bradycardia – (beats per minute)	50-54 if baseline >60	45-49*** if baseline >60	<45 if baseline >60	ER visit or hospitalization for arrhythmia
	45-50 if baseline ≤ 60	40-44 if baseline ≤ 60	<40 if baseline ≤ 60	
Hypertension (systolic) – mmHg	141 – 150	151 – 155	>155	ER visit or hospitalization for malignant hypertension
Hypertension (diastolic) – mmHg	91 – 95	96 – 100	>100	ER visit or hospitalization for malignant hypertension
Hypotension (systolic) – mmHg	85 – 89	80 – 84***	<80	ER visit or hospitalization for hypotensive shock
Respiratory Rate – breaths per minute	17-20	21-25	>25	Intubation

* Subject should be at rest for all vital sign measurements.

**Oral temperature; no recent hot or cold beverages or smoking

*** Medical monitor to use clinical judgment when evaluating bradycardia and hypotension among some healthy subject populations, for example, conditioned athletes.

**Systemic (General)**	**Mild (Grade 1)**	**Moderate (Grade 2)**	**Severe (Grade 3)**	**Potentially Life Threatening (Grade 4)**
Nausea/vomiting	No to mild interference with activity or 1 to 2 episodes/24 hours	Moderate interference with activity or >2 episodes/24 hours	Prevents daily activity, requires outpatient intravenous hydration	Emergency room visit or hospitalization for hypotensive shock
Diarrhea	2 - 4 loose stools or < 400 gm/24 hours	5-6 stools or 400 – 800 gm/24 hours	7or more watery stools or> 800gms/24 hou rs or requires outpatient intravenous hydration	Emergency room visit or hospitalization
Headache	No interference with activity	Repeated use of non-narcotic pain reliever > 24 hours or some interference with activity	Significant; any use of narcotic pain reliever or prevents daily activity	Emergency room visit or hospitalization
Fatigue	No interference with activity	Some interference with activity	Significant; prevents daily activity	Emergency room visit or hospitalization
Myalgia	No interference with activity	Some interference with activity	Significant; prevents daily activity	Emergency room visit or hospitalization

**Systemic (General)**	**Mild (Grade 1)**	**Moderate (Grade 2)**	**Severe (Grade 3)**	**Potentially Life Threatening (Grade 4)**
Illness or clinical AE (as defined according to applicable regulations	No interference with activity	Some interference with activity, not requiring medical intervention	Prevents daily activity and requires medical intervention	Emergency room visit or hospitalization

**Serum***	**Mild (Grade 1)**	**Moderate (Grade 2)**	**Severe (Grade 3)**	**Potentially Life Threatening (Grade 4)**
Sodium – hyponatremia mEq/L	132 – 134	130 – 131	125 – 129	<125
Sodium – hypernatremia mEq/L	148 – 150	151 – 153	>154	Associated with seizures
Potassium – hyperkalemia mEq/L	5.3 – 5.4	5.5 – 5.6	>5.6	Associated with ECG changes
Potassium – hypokalemia mEq/L	3.0 – 3.5	2.5 – 2.9	2.0 – 2.4	≤1.9
Glucose – hypoglycemia mg/dL	65 – 69	55 – 64	45 – 54	<45
Glucose – hyperglycemia<br>fasting mg/dL<br>random mg/dL	100 – 110<br>111– 125	111 – 125<br>126 – 200	>125<br>>200	Insulin requirements or hyperosmolar coma
Blood urea nitrogen mg/dL	21 – 26	27 – 31	>31	Requires dialysis
Creatinine mg/dL	1.5 – 1.7	1.8 – 2.0	2.1 – 2.5	>2.5 or requires dialysis
Calcium – hypocalcemia mg/dL	8.0 – 8.8	7.5 – 7.9	7.0 – 7.4	<7.0
Calcium – hypercalcemia mg/dL	10.6 – 11.0	11.1 – 11.5	11.6 – 12.0	>12.0
Magnesium – hypomagnesemia mg/dL	1.3 – 1.7	1.1 – 1.2	0.9 – 1.0	<0.9
Phosphorous – hypophosphatemia mg/dL	2.3 – 2.5	2.0 – 2.2	1.6 – 1.9	<1.6
Creatine phosphokinase – mg/dL	2.0 – 5.0 x ***ULN	5.1 – 10.0 x ***ULN	10.1 – 20 x ***ULN	>20 x ***ULN
Albumin – hypoalbuminemia g/dL	2.8 – 3.3	2.5 – 2.7	<2.5	--
Total protein – hypoproteinemia g/dL	5.5 – 5.9	5.0 – 5.4	<5.0	--
Alkaline phosphate – increase by factor	2.0 – 2.9 x ULN	3.0 – 5.0 x ULN	>5.0 – 10.0 x ULN	>10.0 x ULN
Liver function tests – ALT, AST increase by factor	1.1 – 2.5 x ULN	2.6 – 5.0 x ULN	5.1 x 10 x ULN	>10 x ULN
Bilirubin – when accompanied by any increase in liver function test increase by factor	1.1 – 1.25 x ULN	1.26 – 1.5 x ULN	1.51 – 1.75 x ULN	>1.75 x ULN

Serum*	Mild (Grade 1)	Moderate (Grade 2)	Severe (Grade 3)	Potentially Life Threatening (Grade 4)
Bilirubin – when liver function test is normal; increase by factor	1.1 – 1.5 x ULN	1.6 – 2.0 x ULN	2.1 – 3.0 x ULN	>3.0 x ULN
Cholesterol	201 – 210	211 – 225	>226	--
Pancreatic enzymes – amylase, and lipase	1.5 – 2.0 x ULN	2.1 – 3.0 x ULN	3.1 – 5.0 x ULN	>5.0 x ULN

AE = adverse event; ALT = alanine aminotransferase; AST = aspartate aminotransferase

* The laboratory values provided in the tables serve as guidelines and are dependent upon institutional normal parameters. Institutional normal references should be provided to demonstrate that they are appropriate.

*** ULN is the upper limit of the normal range.

Hematology *	Mild (Grade 1)	Moderate (Grade 2)	Severe (Grade 3)	Potentially Life Threatening (Grade 4)
Hemoglobin (Female) - gm/dL	10.5-11.5	9.5-10.4	8.0 – 9.4	< 8.0
Hemoglobin (Female) change from baseline value -	Any decrease – 1.5	1.6 – 2.0	2.1 – 5.0	> 5.0
Hemoglobin (Male) - gm/dL	12.5 – 13.5	10.5 – 12.4	8.5 – 10.4	< 8.5
Hemoglobin (Male) change from baseline value –	Any decrease – 1.5	1.6 – 2.0	2.1 – 5.0	> 5.0
WBC Increase - cell/mm^3	10,800- 15,000	15,001 – 20,000	20,001 – 25, 000	> 25,000
WBC Decrease - cell/mm^3	2,500 – 3,500	1,500 – 2,499	1,000 – 1,499	< 1,000
Lymphocytes Decrease - cell/mm^3	750 – 1,000	500 – 749	250 – 499	< 250
Neutrophils Decrease – cell/mm^3	1500-2000	1000-1,499	500-999	< 500
Eosinophils - cell/mm^3	650 – 1500	1501 – 5000	> 5000	Hypereosinophilic
Platelets /mm3	75,000 – 99,000	50,000 – 74,999	20,000 – 49,999	<20,000
PT – increase by factor (prothrombin time)	1.0 – 1.10 x ULN**	1.11 – 1.20 x ULN	1.21 – 1.25 x ULN	> 1.25 ULN
PTT – increase by factor (partial thromboplastin time)	1.0 – 1.2 x ULN	1.21 – 1.4 x ULN	1.41 – 1.5 x ULN	> 1.5 x ULN
Fibrinogen increase - mg/dL	400-500	501-600	>600	--
Fibrinogen decrease - mg/dL	150 – 200	125 – 149	100 – 124	< 100 or associated with gross bleeding or disseminated intravascular coagulation (DIC)

* The laboratory values provided in the tables serve as guidelines and are dependent upon institutional normal parameters. Institutional normal reference ranges should be provided to demonstrate that they are appropriate.

** ULN is the upper limit of the normal range.

**Urine ***	**Mild (Grade 1)**	**Moderate (Grade 2)**	**Severe (Grade 3)**	**Potentially Life Threatening**
Protein	Trace	1+	2+	Hospitalization or dialysis
Glucose	Trace	1+	2+	Hospitalization for hyperglycemia
Blood (microscopic) – red blood cells per high power field (rbc/hpf)	1-10	11 – 50	> 50 and/or gross blood	Hospitalization or packed red blood cells (PRBC)

* The laboratory values provided in the tables serve as guidelines and are dependent upon institutional normal parameters. Institutional normal reference ranges should be provided to demonstrate that they are appropriate.

APPENDIX C: IMINOSUGAR CLINICAL SIGNS

Species	Drug	Finding[a]
Human	Zavesca®	Gastrointestinal<br>diarrhea<br>flatulence<br>abdominal pain<br>nausea<br>vomiting<br>bloating<br>dyspepsia
		Metabolic and Nutritional Disorders<br>weight decrease
		Central and Peripheral Nervous System<br>headache<br>tremor<br>dizziness<br>leg cramps<br>paresthesia<br>migraine
		Vision Disorders<br>visual disturbance
		Musculoskeletal Disorders<br>cramps
		Platelet, Bleeding, and Clotting Disorders<br>thrombocytopenia
		Reproductive Disorders, Female<br>menstrual disorder
	Glyset®	Gastrointestinal<br>diarrhea<br>flatulence<br>abdominal pain<br>ileus, including paralytic ileus<br>nausea<br>abdominal distension<br>pneumatosis cystoides intestinalis
		Dermatologic<br>skin rash
		Abnormal Laboratory Findings<br>low serum iron<br>reductions in hemoglobin or hematologic indices
	Precose®<br>(acarbose)	Gastrointestinal<br>diarrhea<br>flatulence<br>abdominal pain and/or distension<br>ileus, including paralytic ileus

Species	Drug	Finding[a]
		nausea<br>loss of appetite<br>clay-colored stools<br>unusual bleeding (nose, mouth, vagina, or rectum<br>pneumatosis cystoides intestinalis
		Abnormal Laboratory Findings<br>low serum iron<br>reductions in hemoglobin or hematologic indices
		Dermatologic<br>purple or red pinpoint spots under the skin<br>mild rash<br>itching
Mouse	UV-4B	Gastrointestinal<br>neutrophilic infiltration and erosion/ulcer in rectum F100)<br>fecal abnormalities (>250)
		Clinical Chemistry<br>increased AST (men) ≥600 and women 600)
		Hematologic and Immunologic Effects<br>decrease in red cell mass, RBC count, HGB, and HCT (100)<br>decrease in thymus weight (men)= ≥25 and women 100)<br>lymphoid depletion in thymus (F ≥25 and M 100)
		Neurologic Effects<br>no effect in FOB
		Cardiovascular Effects<br>none observed
Rat		Gastrointestinal<br>erosion/ulcer, inflammation, decreased mucus in stomach, hyperplasia/hyperkeratosis (≥25)
		Clinical Chemistry<br>increased AST and ALT (≥10)
		Hematologic and Immunologic Effects<br>decreased PLT and reticulocyte count (≥10)<br>increased WBC and lymphocyte count (men ≥10)<br>extramedullary hematopoiesis decreased in spleen and liver (60)
		Neurologic Effects<br>swollen paws/legs and perioral appearance and red skin, paws, and/or feet (women <60)
		Cardiovascular Effects<br>none observed
Dog	UV-4B	Gastrointestinal<br>salivation and vomitus (60)<br>nonformed feces (≥10)
		Clinical Chemistry<br>increased AST (≥2)<br>increased ALT (≥2)

Species	Drug	Finding[a]
		inverted AST/ALT ratio (10)
		Hematologic and Immunologic Effects<br>decreased PLTs (2)<br>decreased reticulocytes (10)<br>decreased thymus weight (≥2)<br>increased aPTT (≥10)<br>lymphocyte depletion in thymus, spleen, lymph nodes, and GALT (≥2)
		Neurologic Effects<br>none observed
		Cardiovascular<br>decreased arterial pulse pressure (>50 mg/kg)<br>increased diastolic pressure (>50 mg/kg)<br>decreased systolic pressure (>50 mg/kg)

ALT = alanine aminotransferase; aPTT = activated partial thromboplastin time; AST = aspartate aminotransferase; FOB = functional observational battery; GALT = gut-associated lymphoid tissue; HGB = hemoglobin; HCT = hematocrit; RBC = red blood cell; WBC = white blood cell

[a] For mouse, rat, and dog studies, dose presented as mg/kg/dose, TID.

APPENDIX D: SCHEDULE OF EVENTS

Visit number	Visit 1 Screening period	Visit 2 Residential period				Visit 3	Visit 4 Follow-up
		Admission to Clinic	Dosing	In Clinic	Discharge from Clinic	Out-patient	
**Activity\Day**	**Day -28 to -2**	**Day -1**	**Day 1**	**Day 2**	**Day 3**	**Day 4**	**Day 9 ± 1**
Informed consent	X						
Inclusion/exclusion criteria	X	X					
Demographics	X						
Medical/surgical history	X						
Physical examination	X	X[a]	X[a]	X[a]	X[a]	X[a]	X
Height and calculation of BMI	X						
Body weight	X	X			X		X
Vital signs (supine)[b]	X	X	X[c]	X[c]	X[c]	X	X
Orthostatic blood pressure & pulse rate[d]	X	X	X[c]	X[c]	X[c]		
Serology	X						
Urine [drugs of abuse & cotinine /alcohol test]	X	X					
Breath test for alcohol	X	X					
Blood alcohol	X	X					
Serum pregnancy test[e]		X					X
Urine pregnancy test [e]	X						
FSH[f]	X						
Clinical chemistry, hematology, coagulation, & urinalysis	X	X		X	X	X	X
Hematology, clinical chemistry, & coagulation [subset]			X[g]				
Platelet function test[g]		X	X	X	X[h]		
Collection of Stool	X	X	X	X	X		
Fecal occult blood assessment		X	X[i]	X	X		X[i]
ECG[j]	X	X	X	X	X	X	X
Telemetry[k]		X	X				
Randomization[l]			X				

Visit number	Visit 1 Screening period	Visit 2 Residential period				Visit 3	Visit 4 Follow-up
		Admission to Clinic	Dosing	In Clinic	Discharge from Clinic	Out-patient	
**Activity\Day**	**Day -28 to -2**	**Day -1**	**Day 1**	**Day 2**	**Day 3**	**Day 4**	**Day 9 ± 1**
Administration of the investigational product			X				
PK blood sampling			X[m]	X[m]	X[m]	X[m]	
PK pooled urine collections		X[n]	X[n]	X[n]	X[n]		
Sham Dosing		X					
Check-in to the clinic		X					
Discharge from the clinic					X		
Concomitant medication	X	X	X	X	X	X	X
AEs			X	X	X	X	X

[a] Abbreviated physical examination (general appearance, heart, lungs, skin and abdomen), and brief neurological examination (mental status, motor system, and sensory system) will be done at check-in, discharge, and on Day 4. In addition, brief physical examinations focused on the examination of skin and oropharyngeal mucosa for evidence of bleeding and/or bruising (oral [gums] and cutaneous) will be performed daily. Symptom directed/targeted physical examinations will be done at other study time points based on subject symptoms.

[b] Vital signs (blood pressure and pulse rate including oral temperature and respiratory) taken after 10 minutes supine rest; should be taken after ECG recordings (1 hour window for pre-dose procedures).

[c] At pre-dose, 1, 2, 3, 4, 6, 9, 12 24, and 48 hours after the administration of the investigational product. An additional earlier assessment may be included, if t_{max} is ≤1 hour.

[d] Blood pressure and pulse rate taken after 2 minutes in standing position.

[e] Females only.

[f] Postmenopausal females only.

[g] A subset hematology (PLT count), serum chemistry (AST, ALT), and coagulation (INR, PT, aPTT) assessments will be collected at 6 and 12 hours post-dose. A quantitative platelet function test with PFA100 will also be assessed at 6 and 12 hours post-dose. (Baseline value should not be considered for eligibility)

[h] A quantitative platelet function test with PFA100 will only be assessed beyond 24 hours, if changes are observed at the prior assessment.

[i] Fecal occult assessments will be performed starting approximately 6 hours post-dose on every stool sample or less frequent as per discretion of the PI. Fecal occult assessment at follow-up will be performed as per discretion of the PI.

[j] Triplicate 12-lead ECGs at baseline (Day 1 pre-dose); single 12-lead ECGs obtained at the same time each day for all in-clinic days. [ECGs will be repeated for confirmation of any stopping criteria and at the PI's discretion].

[k] Telemetry for at least 4 hours on Day -1 while awake and starting at -0.5 hours on Day 1 through at least 12 hours post-dose.

[l] Pre-dose on Day 1

[m] Blood samples for PK analysis will be collected at pre-dose (0 hr) and at 0.5, 1, 1.5, 2, 2.5, 3, 4, 6, 9, 12, 18, 24, 36, 48 hours post-dose; (1 hour window for pre-dose procedures). The sampling times may be modified based on PK results from prior cohorts.

[n] Pooled urine samples for PK analysis will be collected at pre-dose (-12 to 0 hr) and from 0 to 6, 6 to 12, 12 to 24, and 24 to 48 hours post-dose.

AE: adverse event; BMI: body mass index; CRU: clinical research unit; ECG: electrocardiogram; FSH: follicle stimulating hormone; PK: pharmacokinetic

Appendix E: General Treatment Plan for an Anaphylactic Reaction

The CRO has medical emergency drills for study providers which includes emergency assessment and management of anaphylaxis.

General: Call 911 regardless of on-scene care and patient response; Anaphylaxis requires close follow-up in the emergency room setting and if complicated, investigation and follow-up by an allergy specialist.

Assess patients: Use the Airway, Breathing, Circulation, Disability, and Exposure (ABCDE*), approach to assessment and response.

Call clinical trials unit supervisor, page the study physician and ask for help early. Patients having an anaphylactic reaction require the following:

Establish airway by position, by using an Oropharyngeal Airway (OPA) or intubation.

1. High flow oxygen monitored using pulse oximetry:
2. IV fluid challenge (weight based)
3. IM epinephrine 1:1000 with massage at injection sites.
4. Blood pressure monitoring
5. ECG
6. Epinephrine *(give IM unless experienced with IV dosing)*
7. IM doses of 1:1000 epinephrine (repeat after 5 min if no better)
   - Adult 500 micrograms IM (0.5 mL)
8. IV epinephrine to be given only by experienced specialists
   Titrate: Adults 50 micrograms;

Further management:
Transport by advanced life support (ALS) to local emergency department. All anaphylaxis cases require evaluation. Medical Monitors will be paged.
