# Supplementary material for: Randomized single oral dose phase 1 study of safety, tolerability, and pharmacokinetics of Iminosugar UV-4 Hydrochloride (UV-4B) in healthy subjects
Source: PLoS Negl Trop Dis. 2022 Aug 8;16(8):e0010636. doi: 10.1371/journal.pntd.0010636 (PMC9387934; doi:10.1371/journal.pntd.0010636)
Supplement: S3 File — (DOCX) [file pntd.0010636.s006.docx]

Callahan UV4 FIH Data Availability Statement

The authors have submitted participant and pharmacokinetic (PK) data in compliance with the PLOS data policy. Demographic information on study participants is included in the text (Table 1) and is available under the ClinicalTrials.gov Identifier NCT02061358. Tabulated plasma and urinary PK parameters are included as Supplementary Information (SI) -1 and -2. SI-3 includes the values behind the means, standard deviations and other measures reported, including the values used to build graphs. Per standard practice, raw data (LC-MS/MS mass chromatogram areas for samples and standard curves) have been processed to generate concentration data which are provided as Tables. The Protocol (final version v7.0) is provided as SI-4.
