# Supplementary material for: Randomized single oral dose phase 1 study of safety, tolerability, and pharmacokinetics of Iminosugar UV-4 Hydrochloride (UV-4B) in healthy subjects
Source: PLoS Negl Trop Dis. 2022 Aug 8;16(8):e0010636. doi: 10.1371/journal.pntd.0010636 (PMC9387934; doi:10.1371/journal.pntd.0010636)
Supplement: Attachment — Submitted filename: Response to Reviewers, for Resubmission 10May2022.pdf [file pntd.0010636.s007.pdf]

EMERGENT

10 May 2022

Kendall McKenzie
Managing Editor,
PLoS Neglected Tropical Diseases

Dear Editor,

We have received and addressed the thoughtful and useful comments from the editors and reviewers (email from Robert Reiner dated 17Feb2022) on our manuscript titled, "Randomized Single Oral Dose Phase 1 Study of Safety, Tolerability, and Pharmacokinetics of Iminosugar UV-4 Hydrochloride (UV-4B) in Healthy Subjects", by Michael Callahan et al. After the first resubmission of the revised manuscript, additional comments were received from the journal (email from Nithya Venkatesan dated 20Apr2022). We hope our revisions and resubmission meet reviewers' and journal expectations, and we would like you to consider the revised manuscript for publication. For convenience and reference, we have summarized our responses to reviewer and editor comments below.

1) **Reviewer #1 comments on Key Review Criteria Required for Acceptance**
"As a phase 1a study of a potential antiviral compound, this is well designed and conducted and meets ethical and regulatory requirements."

Author Response:
The authors thank our colleagues for review of the manuscript and positive comments regarding the study.

2) **Reviewer comments on Introduction:**
"The dose range tested was not justified by any published evidence of the bio-efficacy of this compound against dengue viruses in any animal model. This fact should be acknowledged and explained in the Introduction and Discussion. The authors should drastically shorten the introduction which should clearly state that this compound has been shown to have antiviral properties in vitro and is expected to exert its value in vivo in humans as an antiviral compound. The authors should understand and convey clearly to readers that in humans, dengue viruses cause pathophysiological responses that do respond to treatment. Dengue virus non-structural protein, NS1, is a direct endothelial toxin responsible for the potentially lethal leakage of fluid from the circulatory system that may lead to dengue shock syndrome and death. The use of the word "treatment" in dengue should be reserved for efforts that restore fluid volume or reverse endothelial damage. An antiviral compound may or may not restore endothelial integrity. Careful clinical evaluations will be required to establish this as a therapeutic outcome. Throughout this manuscript, the pharmacological action of UV-4B should be referred to as "antiviral.""

Author Response:
We have shortened the Introduction and ensured that the pharmacological action is referred to as antiviral throughout the manuscript. We agree that an antiviral compound is not likely to reverse endothelial damage or restore fluid loss directly. We have updated the text throughout the manuscript, removing references to “treatment” as requested, to clarify that UV-4 is an antiviral agent. We expect that pathophysiological responses would be reduced by action of an active antiviral drug administered soon after infection but this would have to be evaluated in later studies in infected patients. We have added this in the Discussion per reviewer request.
We have added a brief summary to justify the dose range, which was set both by Regulatory mandate and based on doses predicted to give plasma concentrations in the range known to be effective in animal models. This has been added to the Study Design section.

3) **Specific comments:**
Remove the word "designed" as it is redundant.

Author Response:
This word has been removed from lines 34 and 246.

4) **Reviewer comment on Conclusion:**
“As stated above, this compound should not be referred to as a "treatment" of potentially lethal human dengue virus infections. It is difficult for the reviewer to understand how the authors selected the dosage range for testing with no efficacy data available from an animal model”

Author Response:
We have clarified that treatment refers to anti-viral action against the infection. Cited references include several publications demonstrating impact of UV-4 in animal models of dengue infection. The upper end of the dose range was selected with this information in mind and has been briefly explained in the Study Design.

5) **Reviewer Editorial comment:**
“Several important suggestions have been made above. Satisfactory responses are crucial to the publication of an acceptable phase 1a study”

Author Response:
We hope that the corrections made to the text, as described above, are satisfactory to the reviewer and the editor.

6) **Editorial Directions:**
Figure Files:
While revising your submission, please upload your figure files to the Preflight Analysis and Conversion Engine (PACE) digital diagnostic tool, PACE helps ensure that figures meet PLOS requirements. To use PACE, you must first register as a user. Then, login and navigate to the UPLOAD tab, where you will find detailed instructions on how to use the tool. If you encounter any issues or have any questions when using PACE, please email us at figures@plos.org.

Author Response:
We will upload via PACE and as suggested will contact you if we encounter any issues.

7) **Data Requirements:**
Please note that, as a condition of publication, PLOS' data policy requires that you make available all data used to draw the conclusions outlined in your manuscript. Data must be deposited in an appropriate repository, included within the body of the manuscript, or uploaded as supporting information. This includes all numerical values that were used to generate graphs, histograms etc.

Author Response:
All data necessary to draw the conclusions has been included in the manuscript.

8) **Reproducibility:**
To enhance the reproducibility of your results, we recommend that you deposit your laboratory protocols in protocols.io, where a protocol can be assigned its own identifier (DOI) such that it can be cited independently in the future. Additionally, PLOS ONE offers an option to publish peer-reviewed clinical study protocols.

Author Response:
There are no laboratory protocols described in this manuscript.

9) **Editor Instruction:**
Please ensure that the CRediT author contributions listed for every co-author are completed accurately and in full. At this stage "all Authors" require contributions. Please ensure that the full contributions of each author are acknowledged in the ""Add/Edit/Remove Authors"" section of our submission form.

Author Response:
We have entered CRediT author contributions for all authors in the “Add/Edit/Remove Authors” section of the submission form. Because this information is entered into this form, we have removed the section for author contributions from the manuscript Word document.

10) Editor Instruction:
Please provide separate figure files in .tif or .eps format. You do not need to remove any figures embedded in your manuscript file, so long as you have provided separate figure files. We ask that files are kept under 10MB where possible.

Author Response:
We have generated separate .tif files for all figures. Each file was uploaded into, and evaluated and adjusted by the PACE system. PACE did not indicate any problems with the figures. The adjusted tif files were then downloaded from PACE and subsequently uploaded individually into the submission system.

11) Editor Instruction:
We do not publish any copyright or trademark symbols that usually accompany proprietary names, e.g., (R), (C), or TM (e.g., next to drug or reagent names). Therefore, please remove all instances of trademark/copyright symbols throughout the text…

Author Response:
We have removed all copyright and trademark symbols from the manuscript text. This includes symbols associated with Galafold™ on lines 83 and 427, Dengvaxia® on line no 59, Zavesca® on line no 82, Glyset®, Precose® on line no 84, Basen® on line no 85, Dengvaxia® on line no 97, OraSweet® on line no 184, Zavesca®, Glyset®, Precose® on line no 427,Basen® on line no 428, Zavesca®, and Glyset® on line no 435.

EMERGENT

12) Editor Instruction:

We have noticed that you have uploaded supporting information but you have not included a list of legends. Please add a full list of legends for all supporting information files (including figures, table, and data files) in the manuscript after the references list.

Author Response:

After the References section, we have added a section for “Supporting information”. Here we list the supporting information files, including the file number and a one-line title. Legends further describing the file content are described as optional by PLoSNTD, and have therefore not been included.

13) Editor Instruction:

Please amend your detailed Financial Disclosure statement. This is published with the article, therefore should be completed in full sentences, and contain the exact wording you wish to be published.

a. State what role the funders took in the study. If the funders had no role in your study, please state: “The funders had no role in study design, data collection and analysis, decision to publish, or preparation of the manuscript.”
b. If any authors received a salary from any of your funders, please state which authors and which funders.

Author Response:

We have modified the financial disclosure statement per the instructions stated above. We indicated that the funding agency (NIAID) assisted with study design and monitoring, and data analysis, but not with the decision to publish or preparation of the manuscript. We also indicated which authors received partial salary support from the NIAID contract funding this work. The financial disclosure statement was entered into the online form of the submission system; this text was not included in manuscript Word document.

We thank you for consideration of these revisions.

Sincerely,

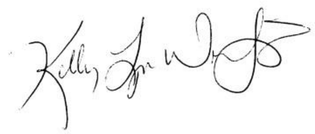

Kelly L. Warfield, Ph.D.
SVP, R&D

400 PROFESSIONAL DR, SUITE 400, GAITHERSBURG, MD 20879 | 240-631-3200
EMERGENTBIOSOLUTIONS.COM
