# Supplementary material for: Randomized single oral dose phase 1 study of safety, tolerability, and pharmacokinetics of Iminosugar UV-4 Hydrochloride (UV-4B) in healthy subjects
Source: PLoS Negl Trop Dis. 2022 Aug 8;16(8):e0010636. doi: 10.1371/journal.pntd.0010636 (PMC9387934; doi:10.1371/journal.pntd.0010636)
Supplement: Attachment — Submitted filename: Response to Reviewers, for Resubmission 26Jun2022.pdf [file pntd.0010636.s008.pdf]

EMERGENT

26 Jun 2022

Kendall McKenzie
Managing Editor,
PLoS Neglected Tropical Diseases

Dear Editor,

We have received and addressed the thoughtful and useful comments from the editors and reviewers (email from Robert C Reiner, dated 10Jun2022) on our manuscript titled, "Randomized Single Oral Dose Phase 1 Study of Safety, Tolerability, and Pharmacokinetics of Iminosugar UV-4 Hydrochloride (UV- 4B) in Healthy Subjects", by Michael Callahan et al. We hope our revisions and resubmission meet reviewers' and journal expectations, and we would like you to consider the revised manuscript for publication. For convenience and reference, we have summarized our responses to reviewer comments below.

1) Reviewer #1 comments on Key Review Criteria Required for Acceptance

"References 17 - 20 describing current concepts of dengue epidemiology, pathogenesis, dengue vaccine efficacy and safety could be more authoritative and accessible".

Reviewer #1 suggested additional useful references for consideration and inclusion in the manuscript.

Author Response:

The authors agree that addition of the suggested references will improve the manuscript. The suggested references have been added to the appropriate location in the manuscript (Introduction and Discussion).

The following references have been added:

1) Sridhar S, Luedtke A, Langevin E, Zhu M, Bonaparte M, Machabert T, et al. Effect of Dengue Serostatus on Dengue Vaccine Safety and Efficacy. N Engl J Med. 2018;379(4):327-40.
2) Glasner DR, Puerta-Guardo H, Beatty PR, Harris E. The Good, the Bad, and the Shocking: The Multiple Roles of Dengue Virus Nonstructural Protein 1 in Protection and Pathogenesis. Annual review of virology. 2018;5(1):227-53.
3) Halstead SB, Russell PK, Brandt WE. NS1, Dengue's Dagger. J Infect Dis. 2020;221:857-60.
4) Halstead SB. Is dengue vaccine protection possible? Clin Infect Dis. 2022 Jan 7;74(1):156-160.

EMERGENT

5) Halstead SB. Vaccine-Associated Enhanced Viral Disease: Implications for Viral Vaccine Development. BioDrugs : clinical immunotherapeutics, biopharmaceuticals and gene therapy. 2021;35(5):505-15.

We thank you for consideration of these revisions.

Sincerely,

Kelly L. Warfield, Ph.D.
SVP, R&D
